# Genomic diversity and ecological distribution of marine *Pseudoalteromonas* phages

**DOI:** 10.1007/s42995-022-00160-z

**Published:** 2023-01-20

**Authors:** Kaiyang Zheng, Yue Dong, Yantao Liang, Yundan Liu, Xinran Zhang, Wenjing Zhang, Ziyue Wang, Hongbing Shao, Yeong Yik Sung, Wen Jye Mok, Li Lian Wong, Andrew McMinn, Min Wang

**Affiliations:** 1 College of Marine Life Sciences, Institute of Evolution and Marine Biodiversity, and Frontiers Science Center for Deep Ocean Multispheres and Earth System, Ocean University of China, Qingdao, 266100 China; 2 UMT-OUC Joint Center for Marine Studies, Qingdao, 266003 China; 3 Institute of Marine Biotechnology, Universiti Malaysia Terengganu (UMT), 21030 Kuala Nerus, Malaysia; 4 Institute for Marine and Antarctic Studies, University of Tasmania, Hobart, Australia; 5 Haide College, Ocean University of China, Qingdao, 266100 China; 6 The Affiliated Hospital of Qingdao University, Qingdao, 266000 China

**Keywords:** *Pseudoalteromonas*, Bacteriophages, Genome, Classification, Ecology

## Abstract

*Pseudoalteromonas*, with a ubiquitous distribution, is one of the most abundant marine bacterial genera. It is especially abundant in the deep sea and polar seas, where it has been found to have a broad metabolic capacity and unique co-existence strategies with other organisms. However, only a few *Pseudoalteromonas* phages have so far been isolated and investigated and their genomic diversity and distribution patterns are still unclear. Here, the genomes, taxonomic features and distribution patterns of *Pseudoalteromonas* phages are systematically analyzed, based on the microbial and viral genomes and metagenome datasets. A total of 143 complete or nearly complete *Pseudoalteromonas*-associated phage genomes (PSAPGs) were identified, including 34 *Pseudoalteromonas* phage isolates, 24 proviruses, and 85 *Pseudoalteromonas*-associated uncultured viral genomes (UViGs); these were assigned to 47 viral clusters at the genus level. Many integrated proviruses (*n* = 24) and filamentous phages were detected (*n* = 32), suggesting the prevalence of viral lysogenic life cycle in *Pseudoalteromonas*. PSAPGs encoded 66 types of 249 potential auxiliary metabolic genes (AMGs) relating to peptidases and nucleotide metabolism. They may also participate in marine biogeochemical cycles through the manipulation of the metabolism of their hosts, especially in the phosphorus and sulfur cycles. Siphoviral and filamentous PSAPGs were the predominant viral lineages found in polar areas, while some myoviral and siphoviral PSAPGs encoding transposase were more abundant in the deep sea. This study has expanded our understanding of the taxonomy, phylogenetic and ecological scope of marine *Pseudoalteromonas* phages and deepens our knowledge of viral impacts on *Pseudoalteromonas*. It will provide a baseline for the study of interactions between phages and *Pseudoalteromonas* in the ocean.

**Supplementary Information:**

The online version contains supplementary material available at 10.1007/s42995-022-00160-z.

## Introduction

Viruses are the most abundant life entities in the ocean and play a vital role in biogeochemical several cycles (Ortmann and Suttle 2005; Xie et al. 2021). Bacteriophages are the most abundant marine viruses (Breitbart 2012), leading to approximately 20–40% of daily bacterial mortality (Suttle 1994). Viral infection and lysis not only contribute to the dissolved organic matter pool through the ‘viral shunt’ and ‘Microbial Carbon Pump’ (Ducklow et al. 2001; Jiao et al. 2010), but also enhance the carbon export from surface to deep waters through the ‘viral shuttle’ (Zimmerman et al. 2020). During the past few decades, phage isolation and metagenomic studies have greatly expanded our knowledge of marine viruses (Dion et al. 2020). However, most studies of marine phages were associated with some dominant marine microbes, such as Cyanobacteria, *Roseobacter* and SAR11 clade (Biller et al. 2015; Zhan and Chen 2019; Zhang et al. 2021a, b).

*Pseudoalteromonas* within the order Alteromonadales is one of the most ubiquitous copiotrophic, particle-associated or planktonic bacteria. It usually comprises around 2–3% and 14% of the total bacterial communities in the surface ocean and deep sea, respectively (Wei et al. 2021); it commonly accounts for up to 20% of particle-associated bacterial communities (Duhaime et al. 2017). Members of the *Pseudoalteromonas* genus are widely known for their special environmental adaption abilities, which help them to survive in extreme habitats, such as deep seas and polar areas (Liu et al. 2019; Medigue et al. 2005; Yong et al. 2009). Although *Pseudoalteromonas* is only found in marine environments, it can also adapt to a parasitic or symbiotic lifestyle with a variety of marine eukaryotic organisms, such as metazoa and algae (Enger et al. 1987; Holmstrom and Kjelleberg 1999). In addition, *Pseudoalteromonas* uses a special genome replication strategy (Yu et al. 2021) and has a broad metabolic capacity in nutrient utilization, including insoluble polysaccharides (Xu et al. 2021), d-amino acid (Yu et al. 2021). It can also produce a wide variety of biologically active natural products, such as, antimicrobial, antifouling and algicidal substances (Feher et al. 2010; Lovejoy et al. 1998; Zeng et al. 2015).

Compared with the phages infecting *Roseobacter*, Cyanobacteria and SAR11 bacteria, our knowledge of the genomic, phylogenetic and ecological characteristics of *Pseudoalteromonas* phages is still sparse. Currently, 34 *Pseudoalteromonas* phage isolates have been deposited in the NCBI dataset (June, 2022). *Pseudoalteromonas* phages can enhance the motility and chemotaxis of their infected host (Yu et al. 2015), and have been used to evaluate the definition of viral operational taxonomic units (vOTU) (Duhaime et al. 2017).

Here, we show the genomic, taxonomic, and ecological signatures of *Pseudoalteromonas* phages based on the phage isolates, integrated proviruses and UViGs from GenBank and IMG/VR v.3 datasets (Roux et al. 2021), providing an updated insight of *Pseudoalteromonas-*associated phages in the global ocean.

## Results

### General information on *Pseudoalteromonas* phage isolates and UViGs

**Fig. 1:**
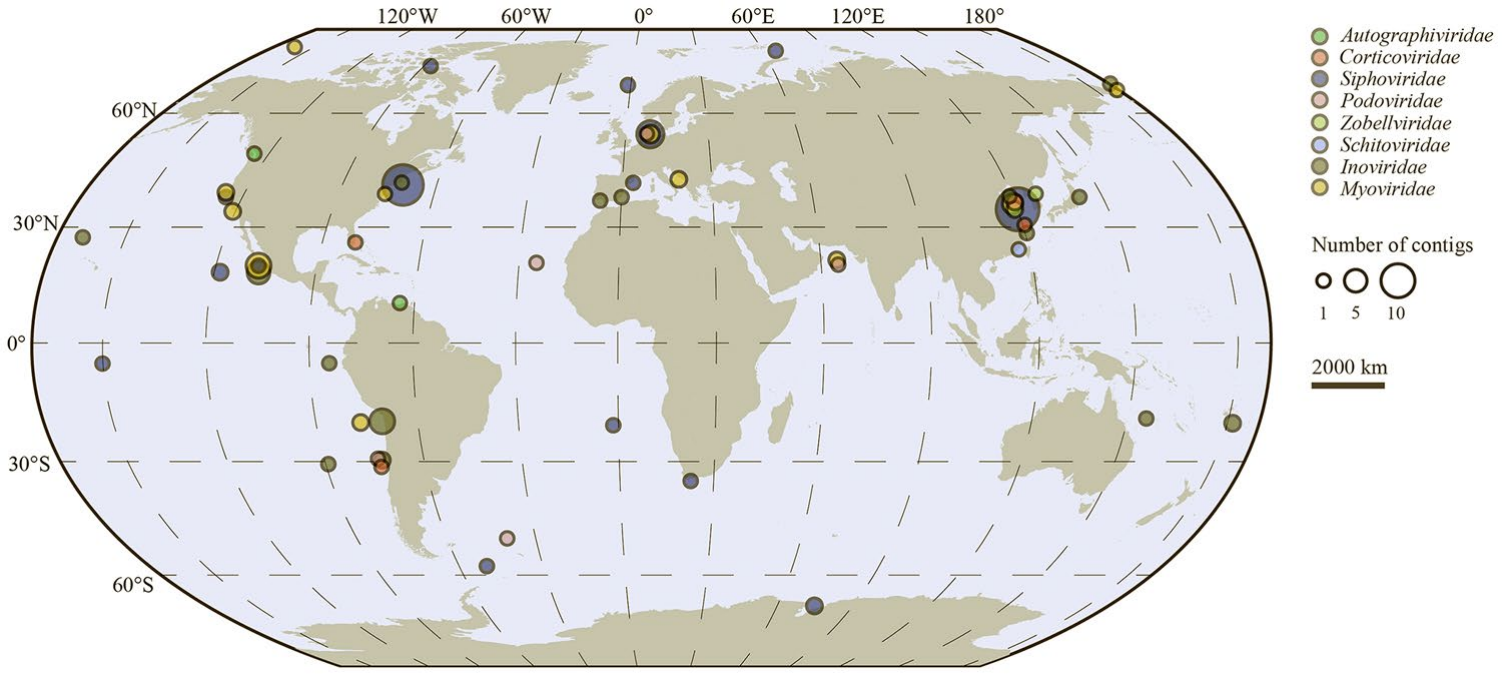
Geographical distribution of all *Pseudoalteromonas* phages. The distribution of 144 *Pseudoalteromonas* phages isolates (35) and uncultured contigs (109) in global marine environment. Each *Pseudoalteromonas*-detection location was represented by a circle proportional to the number of *Pseudoalteromonas* phages. The phages belonging to different viral families were presented by nodes with different colors

Thirty-five *Pseudoalteromonas* phage isolates, from different marine locations and habitats, including the coastal water of Qingdao (China), the Yellow Sea (China), the Bohai Sea (China), the coast water of South Korea, offshore of Helgoland (Germany), the coastal water of Chile, the coastal water of Spain, Atlantic Ocean, offshore of South Africa, and marine metazoans, were collected from the GenBank dataset (July 25, 2021) (Fig. 1 and Supplementary Table S1). Most of these *Pseudoalteromonas* phage isolates infect *Pseudoalteromonas marina* (*n* = 11), *Pseudoalteromonas atlantica* (*n* = 2) or *Pseudoalteromonas phenolica* (*n* = 2) (Supplementary Table S1).

A total of 283 UViGs and integrated proviruses were associated with *Pseudoalteromonas* in the IMG/VR v.3 dataset (Roux et al. 2021). These were assembled and predicted from viral and microbial genomes and metagenomes from pole to pole, and from coastal areas to open oceans (Fig. 1). In this study only UViGs labeled as ‘high-quality’ by CheckV (Nayfach et al. 2021), corresponding to greater than 90% genome completeness, were used for further analysis; this precluded the potential impacts of incomplete UViGs to the comparative- and eco-genomic assessments. A total of 85 *Pseudoalteromonas*-associated UViGs and 24 integrated proviruses were recovered after the quality control, hence the number of species-level *Pseudoalteromonas* phages increased by about four times.

The 144 *Pseudoalteromonas* phages, proviruses and associated UViGs were assigned to seven different viral families, including the five tailed families (59 in *Siphoviridae*, 35 in *Myoviridae*, eight in *Podoviridae*, two in *Zobellviridae*, two in *Autographiviridae* and one in *Schitoviridae*) and two non-tailed families (32 in *Inoviridae* and five in *Corticoviridae*) (Supplementary Table S1). One fragmented *Pseudoalteromonas* siphoviral isolate, B8b, was excluded from further analysis.

The 24 *Pseudoalteromonas* integrated proviruses, which included 15 filamentous phages within *Inoviridae*, six myoviruses, two corticoviruses and one siphovirus (Supplementary Table S1), were commonly integrated into the genomes of *Pseudoalteromonas flavipulchra*, *Pseudoalteromonas ruthenica*, and *Pseudoalteromonas rubra* (Supplementary Table S1), which still lack phage isolates. As chronic viral infection is commonly detected in *Pseudoalteromonas* (Duhaime et al. 2017) and this could enhance the motility and chemotaxis of its infected host (Yu et al. 2015), the prevalent viral integrations might play an important role in the survival of *Pseudoalteromonas* in extreme environments.

**Fig. 2:**
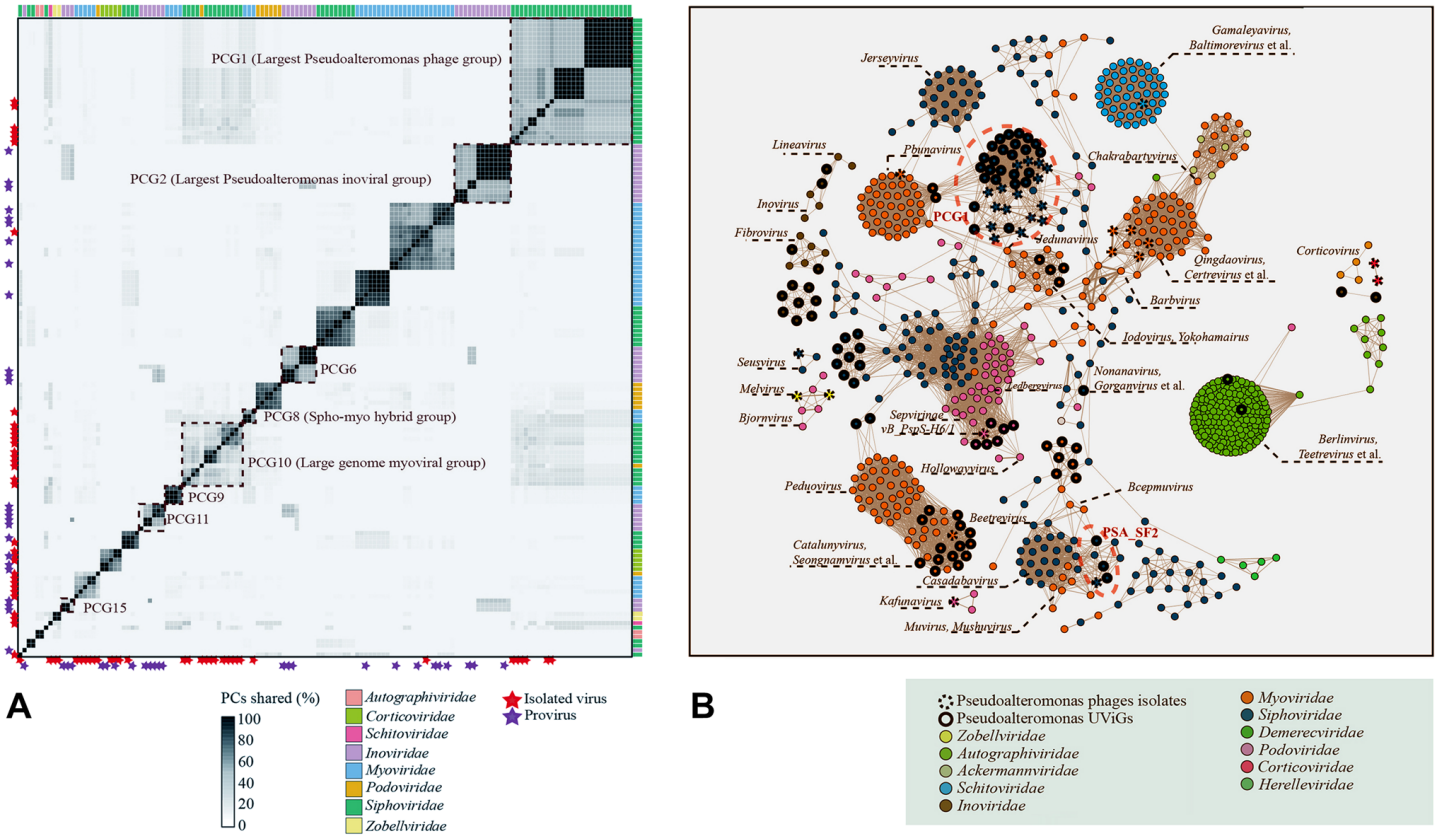
**A** Percentage of average shared protein clusters (PCs) between *Pseudoalteromonas* phages. Roughly, a total of 21 PCs-shared groups (sharing at least 15% PCs among members in each group) were clustered. The representative PCs-shared groups were enclosed by boxes with dash line. Seven different viral families were colored by different boxes on the rightward and top of the heatmap. Isolated viruses and integrated proviruses were labeled as stars with different colors that are located on the leftward and bottom of the heatmap. **B** Binary genome-content based network analysis of *Pseudoalteromonas* phages and reference viral genomes. Uncultured viral genomes (UViGs) and isolates are indicated by circles with a full line and dash line, respectively. The names of genera that have linkages to these *Pseudoalteromonas* phages were labeled in the figure

Based on the analysis of shared protein clusters (PCs), the 143 complete or nearly complete PSAPGs and associated UViGs were classified into 21 PCs-shared groups (PCGs) (Fig. 2A). PCG1 was the largest group, which included six isolates and 22 UViGs within Siphoviridae with an average of 31.1% shared PCs. Some overlap between different PCGs were detected (Fig. 2A), suggesting that horizontal gene transfer might occasionally occur between *Pseudoalteromonas* phage genomes or that these phages might have evolved from a common ancestor.

In further analysis of the taxonomy of *Pseudoalteromonas* phages, it was observed that identified PCGs were not always consistent with the results of the genome-content-based group, probably because the ClustONE algorithm of the latter may ignore some shared low molecular weight proteins in the protein–protein interaction network (Nepusz, et al. 2012). Although the genome-content-based network and the whole genome-based phylogeny are commonly used to assign viral taxon status rather than the PC-shared grouping (Bin Jang et al. 2019; Meier-Kolthoff and Goker 2017), the results of PC-shared clustering could provide an approximate estimate of the likely pangenome of *Pseudoalteromonas* phages, reflecting additional protein diversity and a genomic mosaic pattern in these phages.

### Taxonomic scope of *Pseudoalteromonas* phages in Caudovirales

The genome size of 59 *Pseudoalteromonas* siphoviruses ranged from 30,651 (H105/1) to 78,271 (KB12-38) bp, with the percentage of G + C content from 39.2% (UViG: Station85_MES_COMBINED_FINAL_NODE_626) to 45.8% (Provirus: Gammaproteobacteria_gi_409167369) (Supplementary Table S1). Many PCs were commonly detected among different PCGs within *Siphoviridae*, such as PCG1 and PCG9 (Fig. 2A). The PCG9 included the majority of siphoviral isolates. The genetic relationships between siphoviruses of PCG9 in the PCs-shared analysis were confirmed by the genome-content based network analysis, which formed a viral cluster (VC) with loose linkages (Fig. 2B).

Sixteen VCs of *Pseudoalteromonas* siphoviruses were identified by the genome-content based analysis (Supplementary Fig. S1). The largest VC contained 27 members, including five isolates (H103, TW1, vB_PspS-H40/1, XCL1123 and XC) and 22 UViGs, corresponding to the PCG1 in the PCs-shared analysis (Fig. 2A). Siphoviral isolates were only detected within eight VCs. The other eight VCs only contained UViGs (Fig. 2B) and might represent novel uncultured *Pseudoalteromonas* siphoviral clades, since they lacked any BLAST-based detectable similar genome in the GenBank database.

To investigate the taxonomic diversity of *Pseudoalteromonas* siphoviruses, the phage genomes and UViGs were classified and clustered based on the average nucleotide identity (ANI), the Virus Intergenomic Distance Calculator (VIRIDIC) (Moraru et al. 2020), and the Virus Classification and Tree building Online Resource (VICTOR) (Meier-Kolthoff and Goker 2017), This approach attempted to link these results to the viral taxonomy information in the International Committee on Taxonomy of Viruses (ICTV) (Meier-Kolthoff and Goker 2017; Moraru et al. 2020). All *Pseudoalteromonas* siphoviruses were classified into 16 genus-leveled VCs, belonging to five subfamily-leveled VCs (Supplementary Figs. S1 and S2), which was similar to the results of the genome-content based network analysis (Fig. 2B).

The genotypes of the 16 genus-level *Pseudoalteromonas* siphovirus VCs were highly diverse (Supplementary Figs. S1 and S3). PSA_SF5 was the largest subfamily-level VC, containing nine genus-level VCs, which correspond to PCG1 in the PC-shared clustering (Fig. 2A). Nearly all siphoviral isolates are included in PSA_SF5, except two isolates (HS6 and KB12-38). HS6 and KB12-38 might represent two subfamily-level VCs of *Pseudoalteromonas* siphoviruses (Supplementary Fig. S1). HS6 and three UViGs represent a genus-level VC (PSA_SF2) of *Pseudoalteromonas* siphoviruses, which are closely related to many temperate myoviruses in the genome-content based network (Fig. 2B). KB12-38 and nine UViGs were assigned to PSA_SF4 (Supplementary Fig. S1), which contained three different genus-level VCs. KB12-38 was the only phage isolate of PSA_SF4 and contained the largest genome, comprising 87 ORFs. The other two subfamily-level VCs only contained UViGs. Siphoviruses might be the most diverse *Pseudoalteromonas* phage lineage based on the current data because of their wide taxonomic scope compared with other families.

The genome size of the 35 *Pseudoalteromonas* myoviruses ranged from 32,382 (UViG: UViG_3300026207_000002) to 142,204 (J2-1) bp, with a percentage of G + C content from 34.6% (PH357) to 45.8% (UViG: Station23_DCM_ALL_assembly_NODE_71) (Table S1). *Pseudoalteromonas* myoviruses were grouped into five different VCs in the genome-content based network analysis (Fig. 2B), which was in accordance with the subfamily-level VCs in the whole-genome phylogenic tree, the ANI clustering and the VIRIDIC analysis (Supplementary Figs. S4 and S5). Seven isolates (C5a, Maelstrom, J2-1, PH357, H101, HM1 and SL20) were grouped into six genus-level VCs in both the genome-content based network and the whole-genome phylogenic tree (Supplementary Figs. 2B and S4), which was consistent with the ANI clustering and the VIRIDIC analysis (Supplementary Fig. S5).

All myoviruses with relatively large genomes were phage isolates (J2-1, PH357, H101, SL20 and HM1), corresponding to PCG10 in PC-shared clustering (Fig. 2A) and belonging to four different genus-level VCs (*Qingdaovirus*, PSA_MG5, PSA_MG6 and PSA_MG7) within PSA_MF4 (Supplementary Fig. S4). The percentage of shared PCs among the myoviruses with a large genome size was relatively high and ranged from 46.0 to 86.9% (Fig. 2A), suggesting their close genomic relationships. Four myoviruses (PH357, H101, SL20 and HM1) were clustered together and showed monophyly in the whole-genome phylogenic tree (Supplementary Fig. S4). HM1 and SL20 were assigned to PSA_MG7, and no genome homologs were detected from the nt database of GenBank, suggesting both myoviruses might be genomic orphans in the current database. J2-1 was classified into *Qingdaovirus* in 2019 (https://talk.ictvonline.org/taxonomy/p/taxonomy-history?taxnode_id=202007516). The results of the whole-genome phylogenic tree suggest that the five myoviruses with relatively large genomes might represent a *Pseudoalteromonas*-specific subfamily within *Myoviridae*; this was confirmed by the ANI clustering and the results of the VIRIDIC analysis (Supplementary Fig. S5).

Small *Pseudoalteromonas* myoviruses (such as C5a and Maelstrom, with genome sizes of less than 40 kbp) were all temperate phages and classified into six genus-level VCs within four subfamily-level VCs in the whole-genome phylogenic tree, ANI clustering and VICIDIC analysis (Supplementary Figs. S4 and S5). C5a is the founder of *Catalunyavirus*, which is a genus within the subfamily *Peduovirinae* (Fig. S6) (2020). The myoviruses with relatively small genomes were interconnected with some siphoviruses in the shared PCs analysis (PCG 8) and the genome-content based network analysis (Fig. 2A and B). For instance, in the genome-content based network analysis, Maelstrom and *Shewanella* phage 1/41 were clustered with *Pbunavirus* within *Myoviridae*, and closely related to some siphoviruses, especially with *Jerseyvirus* (Fig. 2B).

PSA_MG4 and PSA_MG8 are two genus-level VCs that only include UViGs. PSA_MG4 is a Mu-like genus containing provirus UViG_3300005658_000006 (Supplementary Table S1) and the other six UViGs (Supplementary Fig. S4). PSA_MG8 was clustered together with eight phage isolates in the whole-genome phylogenic tree, implying their close genomic relationship.

The 13 *Pseudoalteromonas* podoviruses with a short-tailed morphology include five isolates (pYD6-A, HP1, vB_PspS_H6/1, RIO-1 and PH1) and eight UViGs, and these were assigned to four different families, including *Podoviridae* (*n* = 8), *Zobellviridae* (*n* = 2), *Autographiviridae* (*n* = 2) and *Schitoviridae* (*n* = 1). Three defined viral genera have been assigned to six *Pseudoalteromonas* phages, including *Melvirus*, *Kafunavirus* and *Matsuvirus*. The genome size of the phages ranged from 36,753 (vB_PspS-H6/1) to 76,802 (pYD6-A) and the percentage of G + C content ranged from 38.7% (pYD6-A) to 48.7% (UViG_3300027861_000083, Supplementary Table S1). Short-tailed *Pseudoalteromonas* phages were clustered into five VCs in the genome-content based network analysis (Fig. 2B). As *Zobellviridae* and *Schitoviridae* are two recently proposed viral families from *Podoviridae* and contain only 11 assigned members in the ICTV dataset, the whole-genome phylogenic tree of two zobellviruses and one schitovirus were constructed together with podoviruses.

The pYD6-A was clustered into a VC containing eight different genera (*Gamaleyavirus*, *Baltimorevirus*, *Litunavirus*, *Jwalphvirus*, *Ithacavirus*, *Johnsonvirus*, *Equatrovirus* and *Luzseptimavirus*) in the genome-content based network analysis (Fig. 2B), which all belong to *Schitoviridae*, a novel viral family with *Escherichia* virus N4 as the archetype (2020) (Wittmann et al. 2020). Currently, pYD6-A is the only reported *Pseudoalteromonas* schitovirus (Fig. 2B).

vB_PspS-H6/1 and six UViGs were clustered into a VC in the genome-content based network analysis, and confirmed as a subfamily-level VC by the whole-genome phylogenic analysis and VICTOR (Fig. 2B, Supplementary Figs.S7 and S9). Two genus-level VCs were assigned for these *Pseudoalteromonas* podoviruses within PSA_PF1 based on the results of the VICTOR, VIRIDIC and ANI clustering (Supplementary Figs. S7 and S8).

No T7-like *Pseudoalteromonas* phage has been isolated so far, but two T7-like UViGs were associated with *Pseudoalteromonas* through a genomic similarity analysis between viruses and hosts (Roux et al. 2021). The genome size and G + C content of two UViGs are 37,635 (UViG_3300027861_000083) and 37,679 (UViG_3300009132_000015) kbp, and 48.7% and 48.3%, respectively (Supplementary Table S1). In the genome-content-based network analysis. Both UViGs were grouped with the other 70 identified autographiviral genera in the genome-content-based network (Fig. 2B) and were classified into PSA_AG1, within the subfamily *Studiervirinae* by VICTOR (Supplementary Fig. S10).

### Taxonomic scope of non-tailed *Pseudoalteromonas* phages

A total of 37 non-tailed phage genomes were associated with *Pseudoalteromonas*, including 32 filamentous phages within *Inoviridae* (one isolate, 16 UViGs and 15 integrated proviruses) and five corticoviruses (three isolates and two integrated proviruses).

Filamentous phages are extremely diverse and widespread in the entire prokaryotic world (Roux et al. 2019). Although only one *Pseudoalteromonas* inoviral isolate has been reported, chronic infections of filamentous phages might be prevalent in *Pseudoalteromonas*. They might enhance the motility and chemotaxis of their infected host and assist them to adapt to extreme environments such as the deep sea and polar areas (Yu et al. 2015; Wei et al. 2021). In this study, about half of the *Pseudoalteromonas* filamentous phages are integrated proviruses (*n* = 15). The genome size and G + C content of the 32 filamentous phages ranged from 5,991 (UViG_3300002511_000057) to 15,442 (UViG_2551306111_000001) bp, and from 34.6% (UViG_2568526121_000001) to 44.4% (UViG_2671180867_000001), respectively. Five groups of *Pseudoalteromonas* filamentous phages were observed in the PCs-shared analysis, including PCG2, PCG6, PCG11, PCG15 and PCG20 (Fig. 2A). Fourteen filamentous phages were clustered into a VC containing four sub-VCs in the genome-based content network analysis, while the other 18 filamentous phages are singletons (Fig. 2B). Fourteen putative genus-level VCs were assigned by VIRIDIC and ANI clustering and all belonged to the subfamily-level VC, PSA_IF1, which is consistent with the results of the whole-genome phylogenic tree (Supplementary Figs. S11, S12 and S13).

*Corticoviridae* have only rarely been. reported in public databases; only five corticoviruses have been reported in the GenBank dataset (July 25, 2021) and most of them (*n* = 3) infect *Pseudoalteromonas*. In addition, two integrated proviruses were associated with *Pseudoalteromonas* (Supplementary Table S1). As non-tailed virus-like particles accounted for 51–92% of viruses in global surface oceans, the diversity of these rare non-tailed dsDNA viruses, including *Corticoviridae* and *Autolykiviridae*, remains cryptic (Brum et al. 2013; Kauffman et al. 2018).

### Auxiliary metabolic genes (AMGs) in PSAPGs

**Fig. 3:**
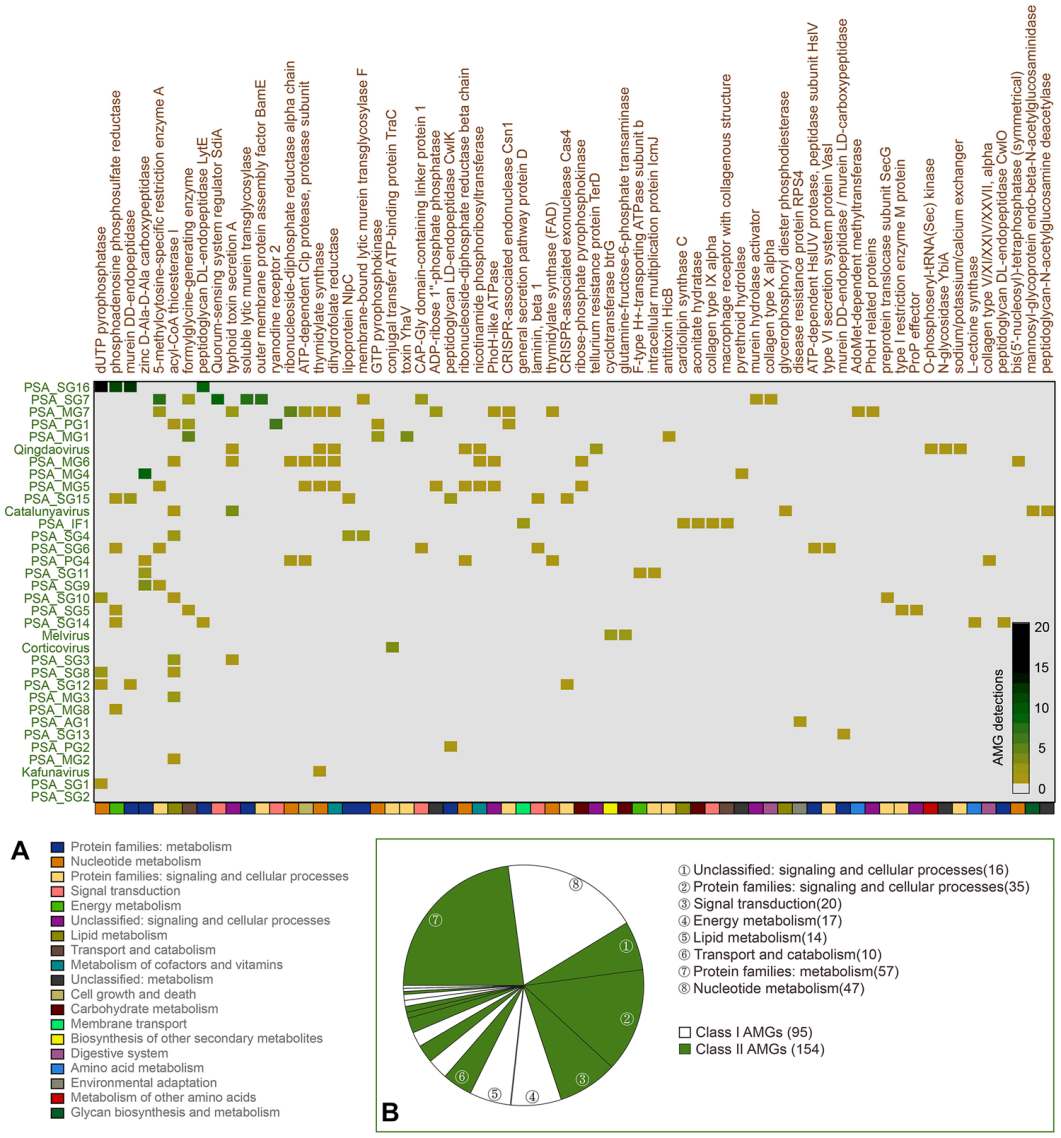
**A** Potential auxiliary metabolism genes (AMGs) encoded by *Pseudoalteromonas* phages from 34 genera and proposed genera, including 239 AMGs belonging to 66 metabolism types. The class I and class II AMGs were identified based on their annotated functions. Different metabolism classifications of AMGs were indicated by different color above the heatmap. The legend of metabolism classifications was shown below the heatmap and was sorted by the number of detections in 19 metabolism classifications. **B** The number of Class I and Class II AMGs was indicated by the pie chart. Top the numbers of detections in 8 metabolism classifications were indicated beside fans

A total of 249 potential AMGs were identified from PSAPGs and classified into 66 different types based on annotations against the KEGG Orthologs (KO) database, including 95 class I AMGs and 154 class II AMGs (Fig. 3 and Supplementary Table S2). The class I AMGs encode proteins with metabolic functions that are included in the KEGG metabolism database, the class II AMGs have a peripheral role in metabolism and are absent from KEGG metabolic pathways (e.g., genes involved in membrane protein component or material transporters) (Hurwitz and U'Ren 2016). Potential AMGs associated with nucleotide metabolism (*n* = 47) and protein families-metabolism (*n* = 57) were the most abundant class I and class II AMGs, respectively. For genus-level VCs of PSAPGs, most AMGs were detected from PSA_SG16, whereas no AMGs were detected from PSA_SG2. Nearly half of AMGs (*n* = 29) are specific to single genus-level VCs. Seven genus-level VCs contain only one AMG, including *Kafunavirus*, PSA_MG8, PSA_AG1, PSA_SG13, PSA_PG2, PSA_MG2, and PSA_SG1. The dUTP pyrophosphatase was the most abundant AMG (*n* = 26) in PSAPGs and only detected in siphoviral VCs.

**Fig. 4:**
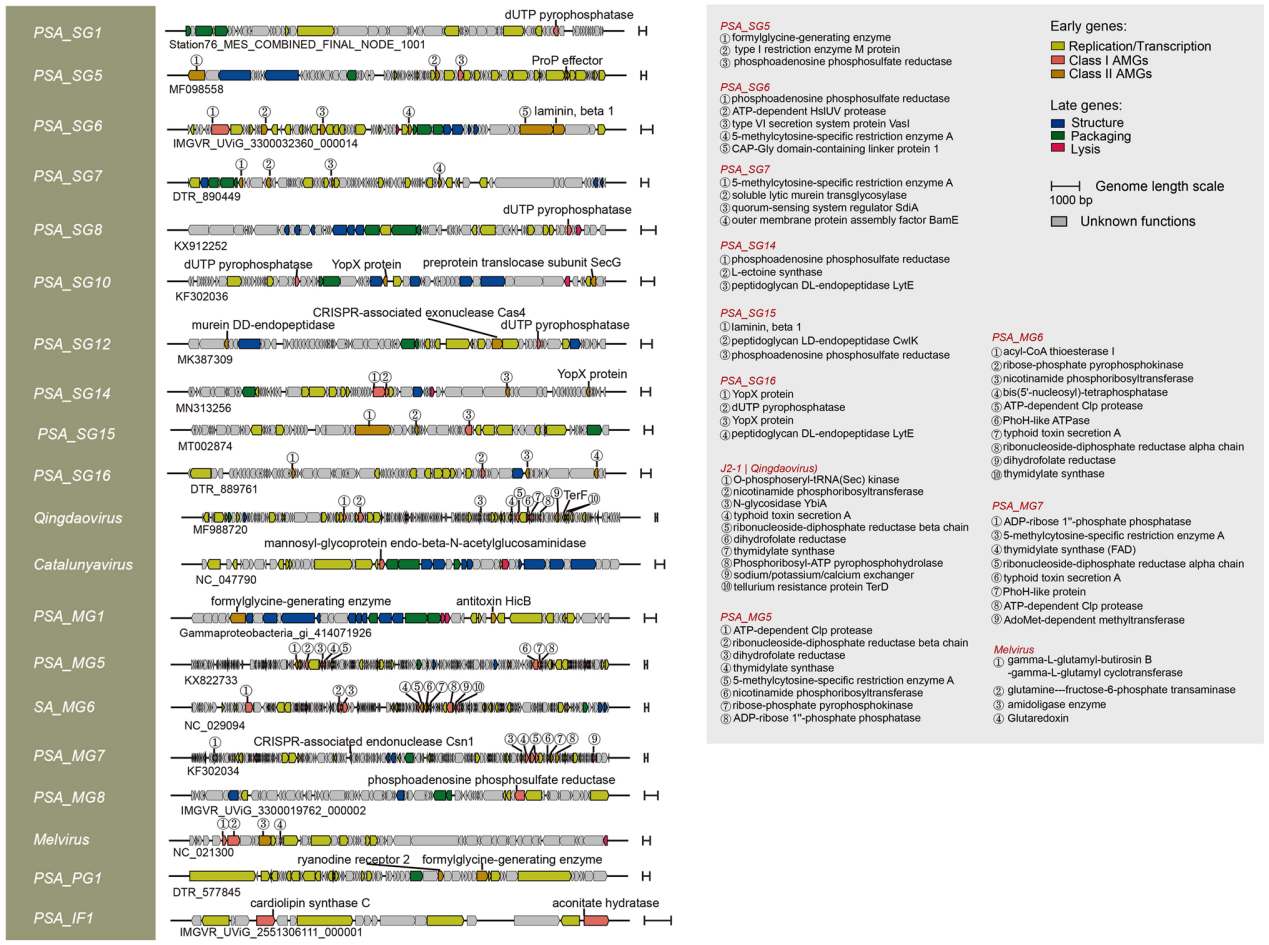
The genome map of 20 representative *Pseudoalteromonas* phage genomes. Six functional modules, including three viral early gene modules (replication/transcription, and all AMGs) and three late viral gene modules (structure, packaging and lysis), were distinguished by diverse colors. The names of potential AMGs were labeled beside corresponding ORFs. The scales of genome length (1000 bp) were indicated on the rightward of each genome

Viral infection can reprogram host metabolism in multiple pathways through the expression of AMGs (Zimmerman et al. 2020). Most class I AMGs (*n* = 47) in PSAPGs were associated with nucleotide metabolism, suggesting that *Pseudoalteromonas* phages might accelerate the nucleotide biosynthesis of their hosts. In addition to the dUTP pyrophosphatase, six other AMGs associated with nucleotide metabolism were detected, including ribonucleoside-diphosphate reductase alpha/beta chains, thymidylate synthase, GTP pyrophosphokinase, thymidylate synthase (FAD) and bis(5′-nucleosyl)-tetraphosphatase (Fig. 3). Most of these participate in the pyrimidine metabolic pathway, while bis(5′-nucleosyl)-tetraphosphatase is involved in purine metabolism (Barnes and Culver 1982). *Pseudoalteromonas* phages might also assist their host cells to degrade glycan (mannosyl-glycoprotein endo-beta-*N*-acetylglucosaminidase) (Suzuki et al. 2002), catalyze the interconversion between nicotinamide and nicotinamide d-ribonucleotide (nicotinamide phosphoribosyltransferase) (Samal et al. 1994) and produce sulphite from 3′-Phosphoadenylyl sulfate (phosphoadenosine phosphosulfate reductase) (Bick et al. 2000). These class I AMGs are widespread in *Pseudoalteromonas* myoviruses, such as *Catalunyavirus* C5a, *Qingdaovirus* J2-1, PSA_MG5 PH357 and PSA_MG6 PH357, respectively (Fig. 4). *Melvirus* RIO-1 may help its host cell to synthesis neomycin, kanamycin and gentamicin biosynthesis through the viral-encoded gamma-l-glutamyl-butirosin B gamma-l-glutamyl cyclotransferase (Kudo and Eguchi 2009) (Fig. 4).

Class II AMGs encoded by PSAPGs may help *Pseudoalteromonas* adapt to different environments. Peptidases are the most abundant class II AMGs in PSAPGs. AMGs related to murein DD-endopeptidase and zinc d-Ala-d-Ala carboxypeptidase are prevalent and specific to PSA_SG16 and PSA_MG4, respectively. AMGs related to PhoH-like proteins were mainly encoded by *Pseudoalteromonas* myoviruses (Fig. 4). Cellular phosphate-starvation induced by viral-encoded PhoH-like protein was frequently related to variations in the bacterial community structure (Wang et al. 2021). The LuxR family transcriptional regulator SdiA was only detected in *Pseudoalteromonas* UViG (DTR_890449), providing evidence that *Pseudoalteromonas* phages may participate in a two-component system, regulating cellular carbon storage, biofilm formation and mediating quorum sensing (Ahmer et al. 1998).

PSAPGs can participate in marine biogeochemical cycles through metabolic reprogramming of host cells by the expression of AMGs (Zimmerman et al. 2020), especially in the phosphorus and sulfur cycles. Five types of AMGs may be involved in this process, including dUTP pyrophosphatase, nicotinamide phosphoribosyltransferase, ADP-ribose 1ʺ-phosphate phosphatase, phosphoadenosine phosphosulfate reductase and formylglycine-generating enzyme. The dUTP pyrophosphatase catalyzes the Mg^2+^-dependent hydrolysis of dUTP to dUMP, providing the substrate for thymidylate synthase with a diphosphate molecule as the end product (Larsson et al. 1996). dUTP pyrophosphatase was frequently detected in *Pseudoalteromonas* siphoviruses, suggesting that the release of inorganic phosphorus from *Pseudoalteromonas* resulting from viral infections should be common. Diphosphate can also be released from ADP-ribose 1ʺ-phosphate phosphatase by the catalysis of ADP-ribose 1ʺ-phosphate phosphatase (Mol et al. 1996), which was encoded by *Pseudoalteromonas* myoviruses, such as PSA_MG5 PH357 and PSA_MG7 HM1 (Fig. 4). Unlike either of the above two AMGs, catalysis of nicotinamide phosphoribosyltransferase requires diphosphate to produce nicotinamide and 5-phospho-alpha-d-ribose 1-diphosphate from nicotinamide d-ribonucleotide (Preiss and Handler 1957), hence viral-encoded nicotinamide phosphoribosyltransferase may manipulate host cells to absorb more phosphate from extracellular environments. For the inorganic sulfur cycle, two *Pseudoalteromonas* siphoviral isolates (PSA_SG14 XCL1123 and PSA_SG14 XC) and two *Pseudoalteromonas* UViGs encoded phosphoadenosine phosphosulfate reductase (Fig. 4), which may manipulate hosts to accelerate the enzymatic reaction in the production of thioredoxin (Berendt et al. 1995). In addition, *Qingdaovirus* J2-1, myoviral integrated proviruses Gammaproteobacteria_gi_414071926 and podoviral UViG DTR_577845 encoded the formylglycine-generating enzyme (Fig. 4), which can catalyze the production of sulfatase-3-oxo-l-alanine with a molecule of hydrogen sulfide and disulfide as by-products. The infection of related *Pseudoalteromonas* phages may manipulate their host cells to release inorganic sulfate to environment.

### Distribution patterns of different *Pseudoalteromonas* phages

**Fig. 5:**
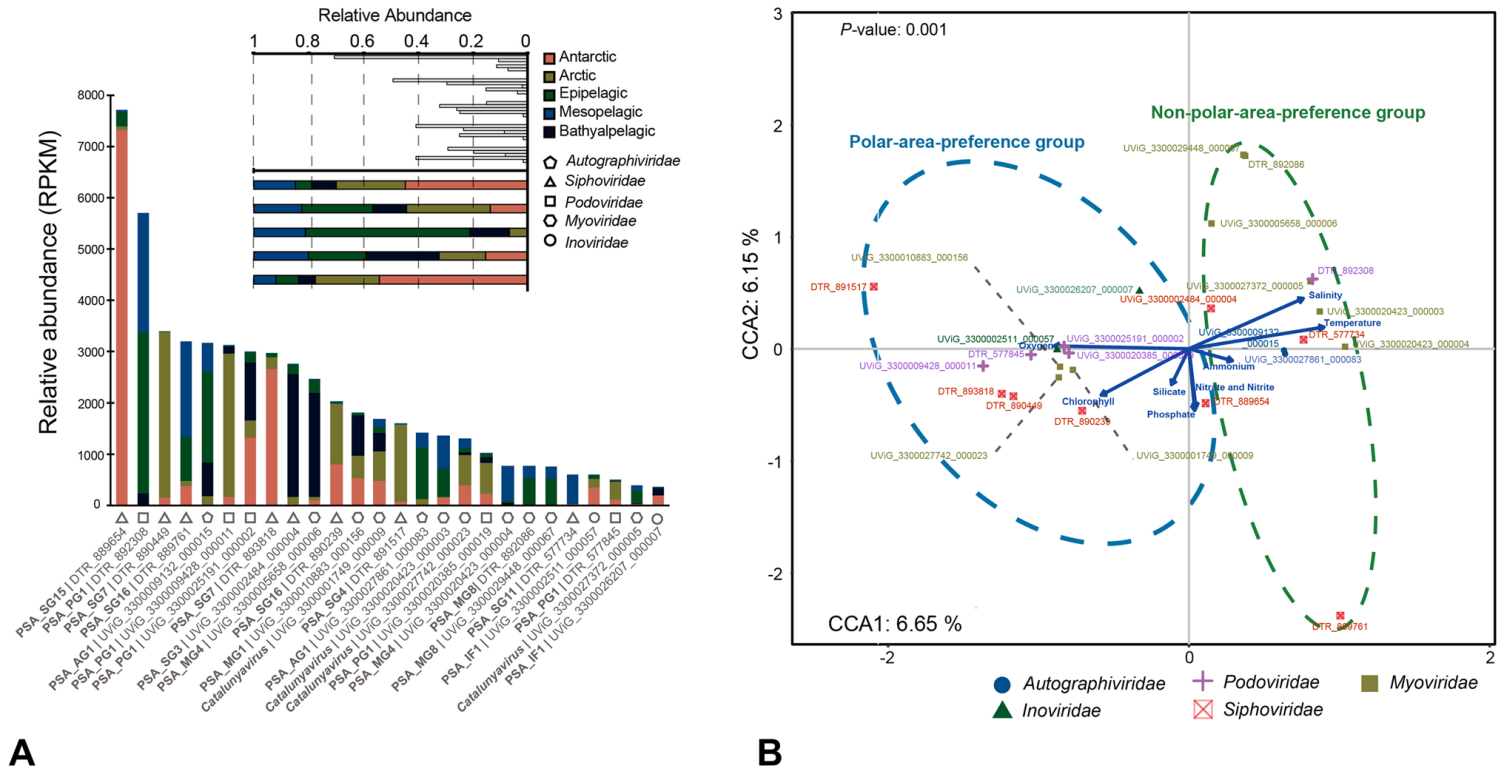
Relative abundance and distribution patterns of *Pseudoalteromonas* phages based on 154 viral metagenomes of Global Ocean Viromes (GOV 2.0) dataset. **A** The top 10 *Pseudoalteromonas* phages with high relative abundance in five ecological zones (epipelagic, mesopelagic, bathypelagic, Arctic and Antarctic). The divergence of distribution patterns for *Pseudoalteromonas* phages in five ecological zones and in five viral families were shown on the top leftward of the bar plot. **B** The CCA fitting module based on the relative abundance of 26 representatives *Pseudoalteromonas* phages and eight types of environmental factors. The bold arrows in blue represent eight types of environmental factors. Different phages were presented as nodes with diverse colors and shapes according to their viral families. The members of the polar-area-preference group and non-polar-area-preference group were highlighted by different colored ellipse boxes with dash lines, respectively. The *p* value of the global Monte-Carlo hypothesis test was indicated in the top leftward of the graph

Distribution patterns of *Pseudoalteromonas* phages and associated UViGs were divergent in the five major viral ecological zones defined by Global Ocean Viromes (GOV 2.0) dataset (Gregory et al. 2019), i.e., Arctic, Antarctic, bathypelagic, temperate and tropical epipelagic and mesopelagic. The relative abundance of the top ten *Pseudoalteromonas* phages genomes is shown in Supplementary Fig. S11. *Pseudoalteromonas* siphoviruses were mainly found in the two polar areas and the mesopelagic zone, while podoviruses and myoviruses were mainly detected in the epipelagic and bathypelagic zones (Fig. 5A). Several of the proposed genera were mainly found in extreme environments, such as in the Antarctic and Arctic (PSA_SG15, PSA_SG7, PSA_AG1, PSA_SG16 and PSA_SG4), and the bathypelagic zone (PSA_SG3 and PSA_MG4); this reinforces the pattern of dominance of *Pseudoalteromonas* and associated phages in polar areas and the deep sea (Wei et al. 2021; Yu et al. 2015).

The siphovirus UViG DTR_889654 within PSA_SG15, assembled from the tropical virome at Station 137 of GOV2.0 (N 14° 12′ W 116° 37′, North Pacific Ocean), was the most abundant *Pseudoalteromonas*-associated phage and was mostly found in Antarctica (Fig. 5A), suggesting an adaptation to cold environments. The podovirus UViG DTR_892308 was the second abundant *Pseudoalteromonas*-associated phage, being mostly found in the epipelagic and mesopelagic zones. This is similar to the distribution of the autographivirus UViG UViG_3300009132_000015 (Fig. 5A). The most abundant *Pseudoalteromonas* myovirus was UViG_3300005658_000006, which was mainly distributed in the bathypelagic zone. Another abundant *Pseudoalteromonas*-associated siphovirus, UViG_3300002484_000004, was also abundant in the bathypelagic zone. Notably, a Mu-like transposase was encoded by both UViG_3300005658_000006 and UViG_3300002484_000004, implying that transposase might be crucial for the life strategy of these *Pseudoalteromonas* phages in the deep sea.

**Fig. 6:**
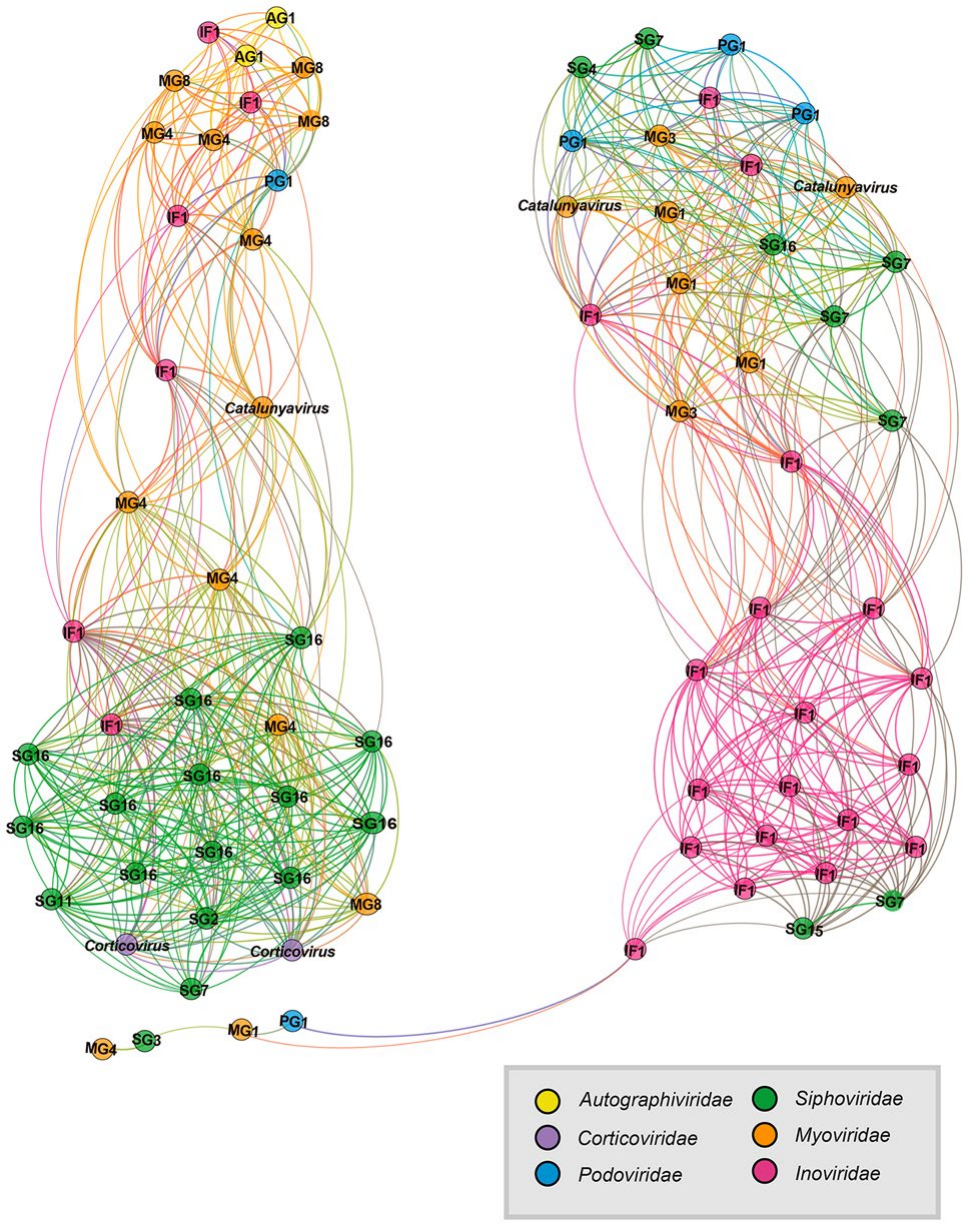
The co-occurrence network based on correlations of distribution patterns for *Pseudoalteromonas* phages. This network was visualized by Gephi under the layout of Force Atlas. Different viral families were indicated by nodes with different colors. The names of genera and proposed genera were indicated beside corresponding nodes

The distribution correlation and divergence within *Pseudoalteromonas* phages is revealed by the co-occurrence network analysis based on the relative abundance of the different viral families. Four sub-modules within two modules were generated based on the Pearson’s correlation coefficient of linkages. In the co-occurrence network, six viral families were generally interconnected, while siphoviruses and filamentous phages (*Inoviridae*) dominated in the two sub-modules, respectively (Fig. 6). At the VC level, both sub-modules were dominated by two single genus/subfamily-level VCs (PSA_SG16 and PSA_IF1), respectively. In addition, the correlation heatmap of relative abundance of *Pseudoalteromonas* phages was also constructed to reflect the distribution pattern divergence of *Pseudoalteromonas* phages. Four main modules with positive correlations were observed (Supplementary Fig. S14), which confirmed the results of the co-occurrence network analysis (Fig. 6). Notably, negative correlations were frequently observed between myoviruses and other viral families. These results demonstrate the special distribution patterns of different viral families from *Pseudoalteromonas* phages in the global ocean for the first time, and revealed their dominance in the deep sea and polar areas.

The distribution patterns of 26 representative *Pseudoalteromonas* phages are likely to be affected by multiple environmental factors. In general, *Pseudoalteromonas* phages could be divided into two groups in the CCA map, those with a polar-area-preference and those with a non-polar-area-preference (Fig. 5B), although different phages are specifically correlated with particular environmental factors. The polar-area-preference group showed a higher relative abundance in polar areas than in other oceanic ecological areas, while the non-polar-area-preference group was not detected in polar areas. The distribution of the polar-area-preference group was mainly related to the concentration of dissolved oxygen, silicate, phosphate, nitrate/nitrite and chlorophyll. The non-polar-area-preference group was mainly related to the concentration of salinity, temperature, and ammonium. The distribution of DTR_889654, the most abundant *Pseudoalteromonas* phage in the Southern Ocean, was mostly related to nitrate/nitrite and phosphate. The ecological distribution of the most abundant *Pseudoalteromonas* podoviruses DTR_892308 was highly correlated with salinity and temperature, which was similar to the *Pseudoalteromonas* autographivirus UViG_3300009132_000015. The distribution of the abundant *Pseudoalteromonas* myovirus UViG_3300005658_000006 and *Pseudoalteromonas* siphovirus UViG_3300002484_000004, which were mainly distributed in bathypelagic zone, were correlated with salinity and temperature (Fig. 5A).

## Discussion

*Pseudoalteromonas* species are widely distributed and abundant in marine environments, especially in the deep sea and polar areas. Dozens of phages infecting *Pseudoalteromonas* have been isolated and characterized, but their genomic diversity and ecological distributions have not been systematically studied. Here, we present a systematic study of the genomic, taxonomic, and ecological characteristics of *Pseudoalteromonas* phages using phage isolates, integrated proviruses and UViGs from global viral and microbial genomes and metagenomes datasets. This input, based on the culture-dependent methods, greatly expands our current understanding of their taxonomy, genomic signatures and ecological importance. The virus-host interactions of *Pseudoalteromonas* need to be investigated further, especially for those from either extreme environments or from those with symbiotic associations with marine organisms. There is only one systematic ecological studies of *Pseudoalteromonas* phages (Duhaime et al. 2011). This study shows that even *Pseudoalteromonas* phages with a relatively high abundance might have not yet been isolated. Hence, these results might provide guidance for the future isolation of these phages. As discussed above, the bacterial niches in different environments can be influenced by viral infections. These impacts could thus be inferred from the distribution patterns of these phages combined with their genomic features (Hurwitz and U'Ren 2016). Notably, *Pseudoalteromonas* phages with abundant AMGs, such as *SG_6*, *Qingdaovirus* and *MG5-7* (carrying at least six AMGs in their genomes) (Fig. 3A), have a relatively low abundance in marine environments. This suggests that those *Pseudoalteromonas* phages with a high relative abundance may need a more compact genome to adapt to the oligotrophic environment, resulting in a loss of nonessential genes. Alternatively, *Pseudoalteromonas* phages distributed in oligotrophic and extreme environments are more likely to adopt a lysogenic infection strategy, which is consistent with previous studies (Howard-Varona et al. 2017). Of the 24 proviruses detected from the genome of *Pseudoalteromonas*, only five genomes could be detected in the GOV 2.0 dataset. The distribution patterns of these five *Pseudoalteromonas* phages (UViG_3300002484_000004, UViG_3300005658_000006, UViG_3300001749_000009, UViG_3300020423_000003 and UViG_3300026207_000007), were strongly associated with extreme environments such as polar areas and the deep sea (Fig. 5A). However, their genotypes were quite diverse being from four existing genera and one proposed genus within three families. Two UViGs belonging to *Catalunyavirus* showed obvious divergence in their distribution patterns, with UViG_3300020423_000003 being more distributed in the epipelagic and mesopelagic zones. However, both the original metagenome (20° 50′ 44.5ʺ N 63° 35′ 06.4ʺ E, central Arabian Sea in the Indian Ocean) and metagenomes having high relative abundances of UViG_3300020423_000003 were all from the open ocean, so UViG_3300020423_000003 might be closely associated with *Pseudoalteromonas* living in oligotrophic environments. For filamentous phages, the relative abundance of UViG_3300002511_000057 was rarely recoded outside the polar areas, which is consistent with the previous studies of filamentous bacteriophages in the Arctic (Yu et al. 2015). However, UViG_3300026207_000007 was mainly distributed below the epipelagic zone and had no detectable relative abundance in other environments. Therefore, this type of filamentous phage is probably mostly associated with the life strategy of *Pseudoalteromonas* in the deep sea. The impact and function of filamentous phages coupling with deep-sea *Pseudoalteromonas* on the environmental adaptation, however, is not clear. Temperate phages and integrated proviruses may thus be crucial for *Pseudoalteromonas* living in these extreme environments.

## Methods

### Information of IMG/VR v.3 database and acquisition of *Pseudoalteromonas* phage genomes

All genomic sequences of *Pseudoalteromonas* phages were retrieved from GenBank (*n* = 34) and IMG/VR v.3 (Roux et al. 2021). The IMG/VR database was established based on the Earth’s Virome protocol (Paez-Espino et al. 2017). The metagenomic assembled viral genomes were filtered in accordance with the protein clustering with viral reference sequences of prokaryotic Virus Orthologous Groups (Grazziotin et al. 2017). The results were confirmed by VirSorter (Roux et al. 2015), VirFinder (Ren et al. 2017), and viral hallmark genes of CheckV database (Nayfach et al. 2021) through custom random forest classifier. Putative integrated proviruses of IMG/VR were identified using BLASTn-based comparison against all published PSAPGs in the IMG database (e-value cutoff of 1.0 × 10^−50^ and percentage identity of 80%). Those UViGs with a cumulative alignment of at least 75% of contig length against an isolated microbial genome were considered integrated proviruses of *Pseudoalteromonas*. Multiple methods were also used to detect potential integrated proviruses, such as PhiSpy (Akhter et al. 2012), Phage_Finder (Fouts 2006), Prophinder (Lima-Mendez et al. 2008) and PHASTER (Arndt et al. 2016). In addition to using HostPhinder (Villarroel et al. 2016) to establish viral-host relationships, all predicted *Pseudoalteromonas* UViGs were identified through two methods, CRISPR spacers’ identity and tRNAs similarity. Related CRISPR spacers were identified from all PSAPGs in the IMG using the CRISPR Recognition Tool (Bland et al. 2007). Identified spacers of all PSAPGs in the IMG database were queried for exact sequence matches against all IMG/VR contigs using the BLASTn-short function with thresholds: e-value threshold of 1.0 × 10^−10^, percentage identity of 95%, using 1 as a maximum target sequence, and allowing only 1–2 SNPs at the 5′ end of the sequence. All tRNAs of these UViGs in IMG/VR was performed with ARAGORN v1.2 and were compared against all *Pseudoalteromonas* genomes and metagenomes in the IMG system using BLAST to select perfect hits (100% query cover with 100% sequence identity), these were regarded as the potential phages that infect *Pseudoalteromonas* (Paez-Espino et al. 2016). Based on the methodology described above, a total of 283 out of 2.4 million viral contigs were predicted to infect *Pseudoalteromonas*.

Only high-quality and reference genomes were selected based on the assessment result of CheckV (Nayfach et al. 2021) to avoid an analytical result bias caused by the presence of incomplete genomes. Nearly all *Pseudoalteromonas* phage isolates were retained except B8b, due to its incomplete genome (Lara et al. 2015). All 143 genomes of *Pseudoalteromonas* phages were included in this study, including 85 UViGs, 24 integrated proviruses and 34 isolates.

Gene calling was performed for all 143 genomes using GeneMarkerS in ‘phage’ mode (Besemer et al. 2001), generating 8,231 ORFs. All of these ORFs were annotated against the NR database (2021.06), Pfam-A database and KEGG Ortholog database, using Diamond BLASTp (Buchfink et al. 2015) (E-value cutoff of 1e^−5^, using 1 as the max target sequence), pfam_scan.pl and KOfam, respectively (Aramaki et al. 2020; Mistry et al. 2021).

### Protein cluster detection of *Pseudoalteromonas* phages

An all-to-all BLASTp was performed for all 8231 ORFs from 143 *Pseudoalteromonas* phages (E-value threshold of 1e^−10^, query cover of 50%, percentage identity of 30%, using 1 as the max target sequence) and the results were transferred to OrthoFinder 2 to detect and group PCs (Emms and Kelly 2015). A total of 6654 ORFs were clustered as 1231 PCs; 1577 of which are ORFans. The percentage of shared PCs was calculated from the average ratio of the number of PCs shared between two genomes and the total number of PCs containing both genomes. A matrix built from percentages of shared PCs was used to construct a heatmap of average PCs shared under the ‘complete’ clustering methods by R. The viral PC-shared groups (PCGs) were generated through the ‘complete’ clustering algorithm. At least 15% PCs were shared among members in each PCG, so 21 PCGs generated based on this criterion.

### Classification of *Pseudoalteromonas* phages

The taxonomic assignment and classification of the *Pseudoalteromonas* phages was performed by the three most used methods in taxonomic classification of ICTV, including vConTACT2, VICTOR and VIRIDIC. To investigate the pan-proteomic diversity of *Pseudoalteromonas* phages, the all-to-all BLASTp was performed for 8231 ORFs of 142 *Pseudoalteromonas* phages and 429,681 ORFs of 12,190 reference viral genomes published in GenBank (June 2021), using the same threshold as that of Orthofinder2 (setting 100,000 as the maximum target sequence). To map these *Pseudoalteromonas* phages into the background of the reference viral pangenome, the results of the all-to-all BLASTp were transferred to vConTACT2 (Bin Jang et al. 2019) to cluster viral contigs through ClusterONE. The binary profile generated by vConTACT2 was used to construct the genome-content based network (Supplementary Table S1), visualized by Gephi using Fruchterman Reingold layout (Bastian et al. 2009).

To identify the viral subfamily/genus of these *Pseudoalteromonas* phages, the whole-genome phylogenic trees at the nucleic acid level was built for the six viral families (*Siphoviridae*, *Myoviridae*, *Podoviridae*, *Autographiviridae* and *Inoviridae*) by VICTOR (Meier-Kolthoff and Goker 2017). VICTOR is able to offer taxonomic classification of phages at both genus- and family-level based on intergenomic distances calculated with the Genome BLAST Distance Phylogeny (GBDP) tool. The phylogenic branch distance cutoff was allowed to be set by VICTOR, generating a high agreement with the ICTV assignment of the virus genomes to taxa of distinct ranks. The *Zobellviridae* family was established based on the results of VICTOR. Although VIRIDIC was also used by ICTV to perform Caudoviral classification (Moraru et al. 2020), the whole-genome phylogenic trees were absent from this tool. To make viral classifications and reveal the phylogenic relationships among these phages, the classification of *Pseudoalteromonas* phages were performed by VICTOR. The phylogenic distance of reference sequences of ICTV, ANI clustering and results produced by VIRIDIC were combined to correct and confirm the results of VICTOR. The nucleotide sequences of all *Pseudoalteromonas* phages and corresponding reference of ICTV were used to calculate the phylogenic tree using parameters: 3 of word size, E-value filter of 0.1, distance threshold of genus = 0.749680 of, distance threshold of speciesm = 0.118980 and distance threshold of subfamily = 0.888940; these are in agreement with the ICTV proposal (Wittmann et al. 2020). These phylogenic trees were visualized with iTOL (Letunic and Bork 2021). The average nucleotide’s identity (ANI) was performed by fastANI (Jain et al. 2018). The viral taxonomic classifications produced by VICTOR were confirmed by VIRIDIC.

### Ecological distribution of *Pseudoalteromonas* phages

A total of 154 viral metagenomes from GOV2.0 were used to investigate the distribution of *Pseudoalteromonas* phages (Pesant et al. 2015). Reads per kilobase per million mapped reads values (RPKM) were used to represent relative abundances of these *Pseudoalteromonas* phages, as in previous reports (Li et al. 2021). Reads from each GOV2.0 metagenome were mapped to a 143 *Pseudoalteromonas* viral contig with BamM v1.7.3 ‘make’, and then coverage profiles were generated across samples performed by CoverM (v0.3.1) with parameters under genome mode: 0.1 of trim-min, 0.9 of trim-max, 0.95 of min-read-percent-identity, 0.75 of min-read-aligned-percent. The results based on all 154 metagenomes of GOV2.0 were classified and summed according to the five main environments identified by Tara Ocean, including epipelagic-, mesopelagic-, bathypelagic-, Antarctic- and Arctic-associated environment.

The different abundances and distribution of these phages from different GOV2.0 metagenomes are depicted in the relative abundance arrays. To investigate the potential correlations in these relative abundance arrays, and to further identify co-occurrence relationships, each array was normalized by *z*-scoring and the Pearson’s correlation coefficient (*R*-value) was calculated for each transient pair. The results were regarded as ‘correlated’ if the *R*-value was > 0.7 (positive correlation) or < − 0.7 (negative correlation); other *R*-values were replaced with a 0. The *R*-value of each pair was regarded as an edge weight to construct a co-occurrence network of distribution patterns. Visualization was by Gephi with a ‘Force Atlas’ layout. The co-occurrence grouping was visualized by a heatmap of *R* with ‘complete’ clustering algorithm.

A canonical correspondence analysis (CCA) was performed to evaluate the relationships between environmental factors and the relative abundances of *Pseudoalteromonas* phages. 108 out of 154 GOV2.0 sampling sites reported all environmental information (temperature, ammonium, nitrate and nitrite, phosphate, silicate, oxygen, salinity, and chlorophyll). Seven sites without detection of 26 representative genomes were removed. The results the other stations were used to perform the CCA. First, the relative abundances of the 26 representative genomes at each site were normalized according to their relative abundances. The relative environmental factor index matrix of 26 representative genomes and relative abundance matrix of these genomes in 101 sites were subjected to vegan (2.5.7) to perform CCA and visualize. The Monte-Carlo hypothesis testing (999 as the permutations) was used to evaluate the significance of this CCA.

## Supplementary Information

Below is the link to the electronic supplementary material.

Supplementary file1 (DOCX 7639 KB)

## Data Availability

All isolated viral genomes are available through the GenBank nucleotides database (https://www.ncbi.nlm.nih.gov/nuccore). All available metagenomic assemble uncultured viral genomes are available through the IMG/VR database (https://img.jgi.doe.gov/cgi-bin/vr/main.cgi), Global Ocean Viromes 2.0 datasets (https://datacommons.cyverse.org/browse/iplant/home/shared/iVirus/GOV2.0), CheckV database (https://portal.nersc.gov/CheckV/checkv-db-v1.0.tar.gz) and GenBank nucleotides database (https://www.ncbi.nlm.nih.gov/nuccore). The linkages to access corresponding sequences have been provided in Supplementary Table 1.

## Abbreviations

CCA: Canonical correspondence analysis
ICTV: International Committee on Taxonomy of Viruses
IMG/VR: Integrated Microbial Genomes and Microbiomes/Viral Resources
PSAPG: *Pseudoalteromonas*-Associated phage genome
PC: Protein cluster
PCG: PCs-shared group
PSA_AF: *Pseudoalteromonas* Autographiviral subfamily
PSA_AG: *Pseudoalteromonas* Autographiviral genus
PSA_IF: *Pseudoalteromonas* Inoviral subfamily
PSA_IG: *Pseudoalteromonas* Inoviral genus
PSA_MF: *Pseudoalteromonas* Myoviral subfamily
PSA_MG: *Pseudoalteromonas* Myoviral genus
PSA_PF: *Pseudoalteromonas* Podoviral subfamily
PSA_PG: *Pseudoalteromonas* Podoviral genus
PSA_SF: *Pseudoalteromonas* Siphoviral subfamily
PSA_SG: *Pseudoalteromonas* Siphoviral genus
UViG: Uncultured viral genome
VC: Viral cluster

## References

[CR1] Ahmer BM, van Reeuwijk J, Timmers CD, Valentine PJ, Heffron F (1998). *Salmonella typhimurium* encodes an SdiA homolog, a putative quorum sensor of the LuxR family, that regulates genes on the virulence plasmid. J Bacteriol.

[CR2] Akhter S, Aziz RK, Edwards RA (2012). PhiSpy: a novel algorithm for finding prophages in bacterial genomes that combines similarity- and composition-based strategies. Nucleic Acids Res.

[CR3] Aramaki T, Blanc-Mathieu R, Endo H, Ohkubo K, Kanehisa M, Goto S, Ogata H (2020). KofamKOALA: KEGG Ortholog assignment based on profile HMM and adaptive score threshold. Bioinformatics.

[CR4] Arndt D, Grant JR, Marcu A, Sajed T, Pon A, Liang Y, Wishart DS (2016). PHASTER: a better, faster version of the PHAST phage search tool. Nucleic Acids Res.

[CR5] Barnes LD, Culver CA (1982). Isolation and characterization of diadenosine 5',5"'-P1, P4-tetraphosphate pyrophosphohydrolase from *Physarum polycephalum*. Biochemistry.

[CR6] Bastian M, Heymann S, Jacomy M (2009) Gephi: an open source software for exploring and manipulating networks. In: Third international AAAI conference on weblogs and social media)

[CR8] Berendt U, Haverkamp T, Prior A, Schwenn JD (1995). Reaction mechanism of thioredoxin: 3'-phospho-adenylylsulfate reductase investigated by site-directed mutagenesis. Eur J Biochem.

[CR9] Besemer J, Lomsadze A, Borodovsky M (2001). GeneMarkS: a self-training method for prediction of gene starts in microbial genomes. Implications for finding sequence motifs in regulatory regions. Nucleic Acids Res.

[CR10] Bick JA, Dennis JJ, Zylstra GJ, Nowack J, Leustek T (2000). Identification of a new class of 5'-adenylylsulfate (APS) reductases from sulfate-assimilating bacteria. J Bacteriol.

[CR11] Biller SJ, Berube PM, Lindell D, Chisholm SW (2015). Prochlorococcus: the structure and function of collective diversity. Nat Rev Microbiol.

[CR12] Bin-Jang H, Bolduc B, Zablocki O, Kuhn JH, Roux S, Adriaenssens EM, Brister JR, Kropinski AM, Krupovic M, Lavigne R, Turner D, Sullivan MB (2019). Taxonomic assignment of uncultivated prokaryotic virus genomes is enabled by gene-sharing networks. Nat Biotechnol.

[CR13] Bland C, Ramsey TL, Sabree F, Lowe M, Brown K, Kyrpides NC, Hugenholtz P (2007). CRISPR recognition tool (CRT): a tool for automatic detection of clustered regularly interspaced palindromic repeats. BMC Bioinformatics.

[CR14] Breitbart M (2012). Marine viruses: truth or dare. Ann Rev Mar Sci.

[CR17] Brum JR, Schenck RO, Sullivan MB (2013). Global morphological analysis of marine viruses shows minimal regional variation and dominance of non-tailed viruses. ISME J.

[CR18] Buchfink B, Xie C, Huson DH (2015). Fast and sensitive protein alignment using DIAMOND. Nat Methods.

[CR20] Dion MB, Oechslin F, Moineau S (2020). Phage diversity, genomics and phylogeny. Nat Rev Microbiol.

[CR21] Ducklow HW, Steinberg DK, Buesseler KO (2001). Upper ocean carbon export and the biological pump. Oceanography.

[CR23] Duhaime MB, Wichels A, Waldmann J, Teeling H, Glockner FO (2011). Ecogenomics and genome landscapes of marine Pseudoalteromonas phage H105/1. ISME J.

[CR22] Duhaime MB, Solonenko N, Roux S, Verberkmoes NC, Wichels A, Sullivan MB (2017). Comparative omics and trait analyses of marine Pseudoalteromonas phages advance the phage OTU concept. Front Microbiol.

[CR24] Emms DM, Kelly S (2015). OrthoFinder: solving fundamental biases in whole genome comparisons dramatically improves orthogroup inference accuracy. Genome Biol.

[CR25] Enger Ø, Nygaard H, Solberg M, Schei G, Nielsen J, Dundas I (1987). Characterization of *Alteromonas denitrificans* sp. nov. Int J Syst Evol.

[CR26] Feher D, Barlow R, McAtee J, Hemscheidt TK (2010). Highly brominated antimicrobial metabolites from a marine *Pseudoalteromonas* sp. J Nat Prod.

[CR27] Fouts DE (2006). Phage_Finder: automated identification and classification of prophage regions in complete bacterial genome sequences. Nucleic Acids Res.

[CR28] Grazziotin AL, Koonin EV, Kristensen DM (2017). Prokaryotic Virus Orthologous Groups (pVOGs): a resource for comparative genomics and protein family annotation. Nucleic Acids Res.

[CR29] Gregory AC, Zayed AA, Conceicao-Neto N, Temperton B, Bolduc B, Alberti A, Ardyna M, Arkhipova K, Carmichael M, Cruaud C, Dimier C, Domı´nguez-Huerta G, Ferland J, Kandels S, Liu Y, Marec C, Pesant S, Picheral M, Pisarev S, Poulain J (2019). Marine DNA viral macro- and microdiversity from pole to pole. Cell.

[CR30] Holmstrom C, Kjelleberg S (1999). Marine *Pseudoalteromonas* species are associated with higher organisms and produce biologically active extracellular agents. FEMS Microbiol Ecol.

[CR31] Howard-Varona C, Hargreaves KR, Abedon ST, Sullivan MB (2017). Lysogeny in nature: mechanisms, impact and ecology of temperate phages. ISME J.

[CR33] Hurwitz BL, U'Ren JM (2016). Viral metabolic reprogramming in marine ecosystems. Curr Opin Microbiol.

[CR35] Jain C, Rodriguez RL, Phillippy AM, Konstantinidis KT, Aluru S (2018). High throughput ANI analysis of 90K prokaryotic genomes reveals clear species boundaries. Nat Commun.

[CR37] Jiao N, Herndl GJ, Hansell DA, Benner R, Kattner G, Wilhelm SW, Kirchman DL, Weinbauer MG, Luo T, Chen F, Azam F (2010). Microbial production of recalcitrant dissolved organic matter: long-term carbon storage in the global ocean. Nat Rev Microbiol.

[CR39] Kauffman KM, Hussain FA, Yang J, Arevalo P, Brown JM, Chang WK, VanInsberghe D, Elsherbini J, Sharma RS, Cutler MB, Kelly L, Polz MF (2018). A major lineage of non-tailed dsDNA viruses as unrecognized killers of marine bacteria. Nature.

[CR41] Kudo F, Eguchi T (2009). Biosynthetic enzymes for the aminoglycosides butirosin and neomycin. Methods Enzymol.

[CR42] Lara E, Holmfeldt K, Solonenko N, Sa EL, Ignacio-Espinoza JC, Cornejo-Castillo FM, Verberkmoes NC, Vaqué D, Sullivan MB, Acinas SG (2015). Life-style and genome structure of marine *Pseudoalteromonas siphovirus* B8b isolated from the northwestern Mediterranean Sea. PLoS ONE.

[CR43] Larsson G, Svensson LA, Nyman PO (1996). Crystal structure of the *Escherichia coli* dUTPase in complex with a substrate analogue (dUDP). Nat Struct Biol.

[CR45] Letunic I, Bork P (2021). Interactive Tree Of Life (iTOL) v5: an online tool for phylogenetic tree display and annotation. Nucleic Acids Res.

[CR47] Li Z, Pan D, Wei G, Pi W, Zhang C, Wang JH, Peng Y, Zhang L, Wang Y, Hubert CRJ, Dong X (2021). Deep sea sediments associated with cold seeps are a subsurface reservoir of viral diversity. ISME J.

[CR48] Lima-Mendez G, Van Helden J, Toussaint A, Leplae R (2008). Prophinder: a computational tool for prophage prediction in prokaryotic genomes. Bioinformatics.

[CR49] Liu J, Zheng Y, Lin H, Wang X, Li M, Liu Y, Yu M, Zhao M, Pedentchouk N, Lea-Smith DJ, Todd JD, Magill CR, Zhang WJ, Zhou S, Song D, Zhong H, Xin Y, Yu M, Tian J, Zhang XH (2019). Proliferation of hydrocarbon-degrading microbes at the bottom of the Mariana Trench. Microbiome.

[CR51] Lovejoy C, Bowman JP, Hallegraeff GM (1998). Algicidal effects of a novel marine Pseudoalteromonas isolate (class Proteobacteria, gamma subdivision) on harmful algal bloom species of the genera Chattonella, Gymnodinium, and Heterosigma. Appl Environ Microbiol.

[CR52] Medigue C, Krin E, Pascal G, Barbe V, Bernsel A, Bertin PN, Cheung F, Cruveiller S, D'Amico S, Duilio A, Fang G, Feller G, Ho C, Mangenot S, Marino G, Nilsson J, Parrilli E, Rocha EPC, Rouy Z, Sekowska A (2005). Coping with cold: the genome of the versatile marine Antarctica bacterium *Pseudoalteromonas haloplanktis* TAC125. Genome Res.

[CR53] Meier-Kolthoff JP, Goker M (2017). VICTOR: genome-based phylogeny and classification of prokaryotic viruses. Bioinformatics.

[CR55] Mistry J, Chuguransky S, Williams L, Qureshi M, Salazar GA, Sonnhammer ELL, Tosatto SCE, Paladin L, Raj S, Richardson LJ, Finn RD, Bateman A (2021). Pfam: the protein families database in 2021. Nucleic Acids Res.

[CR56] Mol CD, Harris JM, McIntosh EM, Tainer JA (1996). Human dUTP pyrophosphatase: uracil recognition by a beta hairpin and active sites formed by three separate subunits. Structure.

[CR57] Moraru C, Varsani A, Kropinski AM (2020). VIRIDIC-A novel tool to calculate the intergenomic similarities of prokaryote-infecting viruses. Viruses.

[CR58] Nayfach S, Camargo AP, Schulz F, Eloe-Fadrosh E, Roux S, Kyrpides NC (2021). CheckV assesses the quality and completeness of metagenome-assembled viral genomes. Nat Biotechnol.

[CR59] Nepusz T, Yu H, Paccanaro A (2012). Detecting overlapping protein complexes in protein-protein interaction networks. Nat Methods.

[CR60] Ortmann AC, Suttle CA (2005). High abundances of viruses in a deep-sea hydrothermal vent system indicates viral mediated microbial mortality. Deep Sea Res Part I.

[CR61] Paez-Espino D, Eloe-Fadrosh EA, Pavlopoulos GA, Thomas AD, Huntemann M, Mikhailova N, Rubin E, Ivanova NN, Kyrpides NC (2016). Uncovering Earth's virome. Nature.

[CR62] Paez-Espino D, Pavlopoulos GA, Ivanova NN, Kyrpides NC (2017). Nontargeted virus sequence discovery pipeline and virus clustering for metagenomic data. Nat Protoc.

[CR63] Pesant S, Not F, Picheral M, Kandels-Lewis S, Le Bescot N, Gorsky G, Iudicone D, Karsenti E, Speich S, Troublé R, Dimier C, Searson S, Tara Oceans Consortium Coordinators (2015). Open science resources for the discovery and analysis of Tara Oceans data. Sci Data.

[CR64] Preiss J, Handler P (1957). Enzymatic synthesis of nicotinamide mononucleotide. J Biol Chem.

[CR66] Ren J, Ahlgren NA, Lu YY, Fuhrman JA, Sun F (2017). VirFinder: a novel k-mer based tool for identifying viral sequences from assembled metagenomic data. Microbiome.

[CR67] Roux S, Enault F, Hurwitz BL, Sullivan MB (2015). VirSorter: mining viral signal from microbial genomic data. PeerJ.

[CR68] Roux S, Krupovic M, Daly RA, Borges AL, Nayfach S, Schulz F, Sharrar A, Carnevali PBM, Cheng JF, Ivanova NN, Bondy-Denomy J, Wrighton KC, Woyke T, Visel A, Kyrpides NC, Eloe-Fadrosh EA (2019). Cryptic inoviruses revealed as pervasive in bacteria and archaea across Earth's biomes. Nat Microbiol.

[CR69] Roux S, Paez-Espino D, Chen IA, Palaniappan K, Ratner A, Chu K, Reddy TBK, Nayfach S, Schulz F, Call L, Neches RY, Woyke T, Ivanova NN, Eloe-Fadrosh EA, Kyrpides NC (2021). IMG/VR v3: an integrated ecological and evolutionary framework for interrogating genomes of uncultivated viruses. Nucleic Acids Res.

[CR71] Samal B, Sun Y, Stearns G, Xie C, Suggs S, McNiece I (1994). Cloning and characterization of the cDNA encoding a novel human pre-B-cell colony-enhancing factor. Mol Cell Biol.

[CR76] Suttle CA (1994). The significance of viruses to mortality in aquatic microbial communities. Microb Ecol.

[CR77] Suzuki T, Yano K, Sugimoto S, Kitajima K, Lennarz WJ, Inoue S, Inoue Y, Emori Y (2002). Endo-beta-N-acetylglucosaminidase, an enzyme involved in processing of free oligosaccharides in the cytosol. Proc Natl Acad Sci USA.

[CR78] Villarroel J, Kleinheinz KA, Jurtz VI, Zschach H, Lund O, Nielsen M, Larsen MV (2016). HostPhinder: a phage host prediction tool. Viruses.

[CR80] Wang L, Zhao J, Wang Z, Li N, Song J, Zhang R, Zhang Y, Jiao N (2021). phoH-carrying virus communities responded to multiple factors and their correlation network with prokaryotes in sediments along Bohai Sea, Yellow Sea, and East China Sea in China. Sci Total Environ.

[CR81] Wei W, Wang L, Fang J, Liu R (2021). Population structure, activity potential and ecotype partitioning of Pseudoalteromonas along the vertical water column of the New Britain Trench. FEMS Microbiol Lett.

[CR82] Wittmann J, Turner D, Millard AD, Mahadevan P, Kropinski AM, Adriaenssens EM (2020). From orphan phage to a proposed new family-the diversity of N4-like viruses. Antibiotics (basel).

[CR83] Xie L, Wei W, Cai L, Chen X, Luo YW (2021). A global viral oceanography database (gVOD). Earth Syst Sci Data.

[CR84] Xu F, Cha QQ, Zhang YZ, Chen XL (2021). Degradation and utilization of alginate by marine Pseudoalteromonas: a review. Appl Environ Microbiol.

[CR85] Yong Y, Li H, Zeng Y, Bo C (2009). Extracellular enzymes of cold-adapted bacteria from Arctic sea ice, Canada Basin. Polar Biol.

[CR87] Yu ZC, Chen XL, Shen QT, Zhao DL, Tang BL, Su HN, Wu ZY, Qin QL, Xie BB, Zhang XY, Yu Y, Zhou BC, Chen B, Zhang YZ (2015). Filamentous phages prevalent in *Pseudoalteromonas* spp. confer properties advantageous to host survival in Arctic sea ice. ISME J.

[CR86] Yu Y, Yang J, Teng ZJ, Zheng LY, Sheng Q, Li PY, Fu HH, Li CY, Chen Y, Zhang YZ, Ding JM, Chen XL (2021). D-alanine metabolism via D-Ala aminotransferase by marine Gammaproteobacteria, *Pseudoalteromonas* sp. CF6-2. Appl Environ Microbiol.

[CR88] Zeng Z, Guo XP, Li B, Wang P, Cai X, Tian X, Zhang S, Yang JL, Wang X (2015). Characterization of self-generated variants in *Pseudoalteromonas lipolytica* biofilm with increased antifouling activities. Appl Microbiol Biotechnol.

[CR89] Zhan Y, Chen F (2019). Bacteriophages that infect marine roseobacters: genomics and ecology. Environ Microbiol.

[CR91] Zhang X, Zhang F, Mi Y, Liu Y, Zheng K, Zhou Y, Jiang T, Wang M, Jiang Y, Guo C, Shao H, He H, He J, Liang Y, Wang M, McMinn A (2021). Characterization and genome analysis of phage AL infecting *Pseudoalteromonas marina*. Virus Res.

[CR92] Zhang Z, Qin F, Chen F, Chu X, Luo H, Zhang R, Du S, Tian Z, Zhao Y (2021). Culturing novel and abundant pelagiphages in the ocean. Environ Microbiol.

[CR93] Zhao Y, Qin F, Zhang R, Giovannoni SJ, Zhang Z, Sun J, Du S, Rensing C (2019). Pelagiphages in the Podoviridae family integrate into host genomes. Environ Microbiol.

[CR96] Zimmerman AE, Howard-Varona C, Needham DM, John SG, Worden AZ, Sullivan MB, Waldbauer JR, Coleman LM (2020). Metabolic and biogeochemical consequences of viral infection in aquatic ecosystems. Nat Rev Microbiol.

