# Correction: Genomic diversity and ecological distribution of marine *Pseudoalteromonas* phages

**DOI:** 10.1007/s42995-023-00166-1

**Published:** 2023-04-07

**Authors:** Kaiyang Zheng, Yue Dong, Yantao Liang, Yundan Liu, Xinran Zhang, Wenjing Zhang, Ziyue Wang, Hongbing Shao, Yeong Yik Sung, Wen Jye Mok, Li Lian Wong, Andrew McMinn, Min Wang

**Affiliations:** 1 College of Marine Life Sciences, Institute of Evolution and Marine Biodiversity, and Frontiers Science Center for Deep Ocean Multispheres and Earth System, Ocean University of China, Qingdao, 266100 China; 2 UMT-OUC Joint Center for Marine Studies, Qingdao, 266003 China; 3 Institute of Marine Biotechnology, Universiti Malaysia Terengganu (UMT), 21030 Kuala Nerus, Malaysia; 4 Institute for Marine and Antarctic Studies, University of Tasmania, Hobart, Australia; 5 Haide College, Ocean University of China, Qingdao, 266100 China; 6 The Affiliated Hospital of Qingdao University, Qingdao, 266000 China

**Correction: Marine Life Science & Technology** 10.1007/s42995-022-00160-z

In this article the graphics relating to Figs. 2 and 3 captions had been interchanged; the figure(s) should have appeared as shown below.

**Fig. 2:**
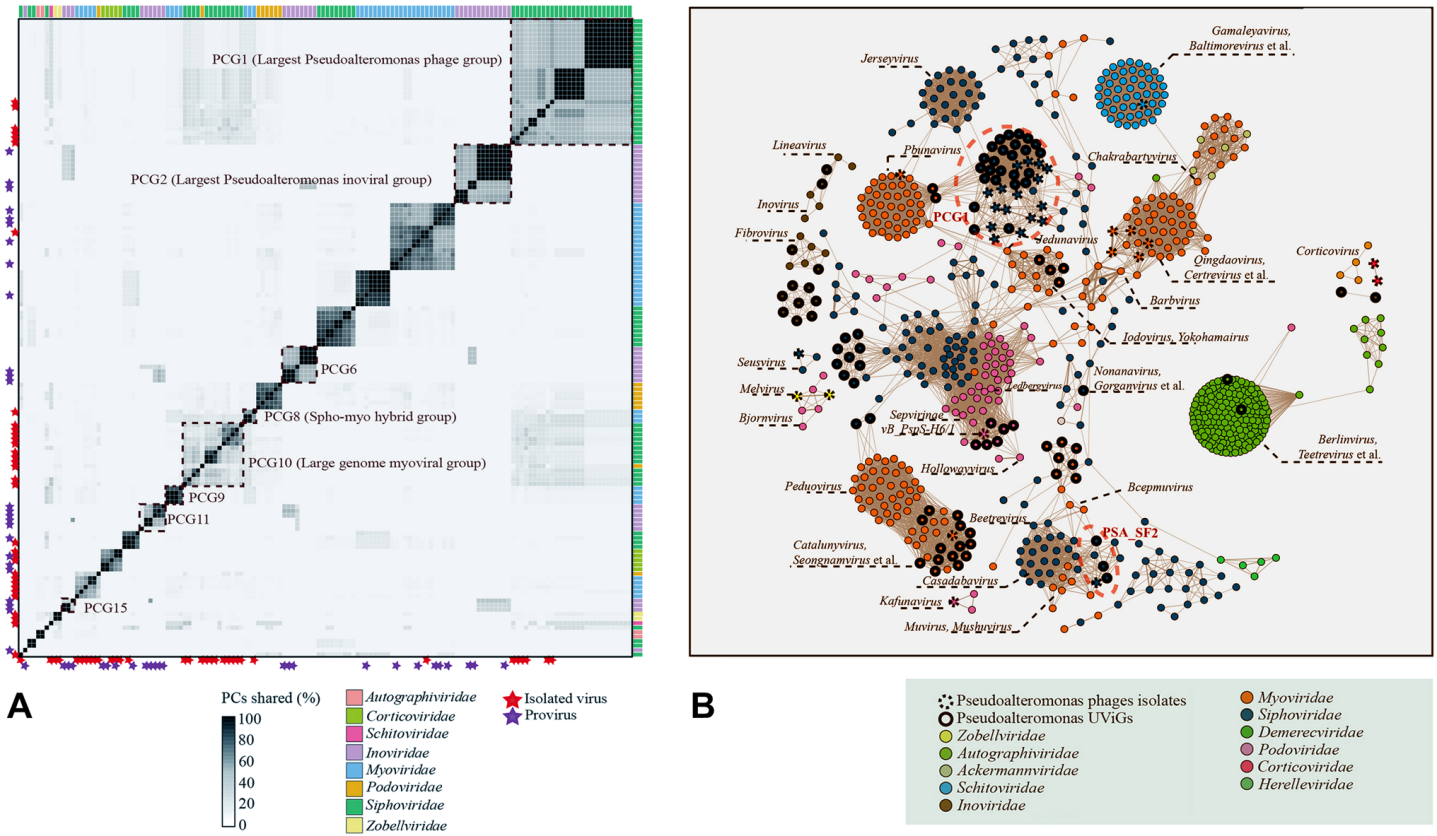
**A** Percentage of average shared protein clusters (PCs) between *Pseudoalteromonas* phages. Roughly, a total of 21 PCs-shared groups (sharing at least 15% PCs among members in each group) were clustered. The representative PCs-shared groups were enclosed by boxes with dash line. Seven different viral families were colored by different boxes on the rightward and top of the heatmap. Isolated viruses and integrated proviruses were labeled as stars with different colors that are located on the leftward and bottom of the heatmap. **B** Binary genome content based network analysis of *Pseudoalteromonas* phages and reference viral genomes. Uncultured viral genomes (UViGs) and isolates are indicated by circles with a full line and dash line, respectively. The names of genera that have linkages to these *Pseudoalteromonas* phages were labeled in the figure

**Fig. 3:**
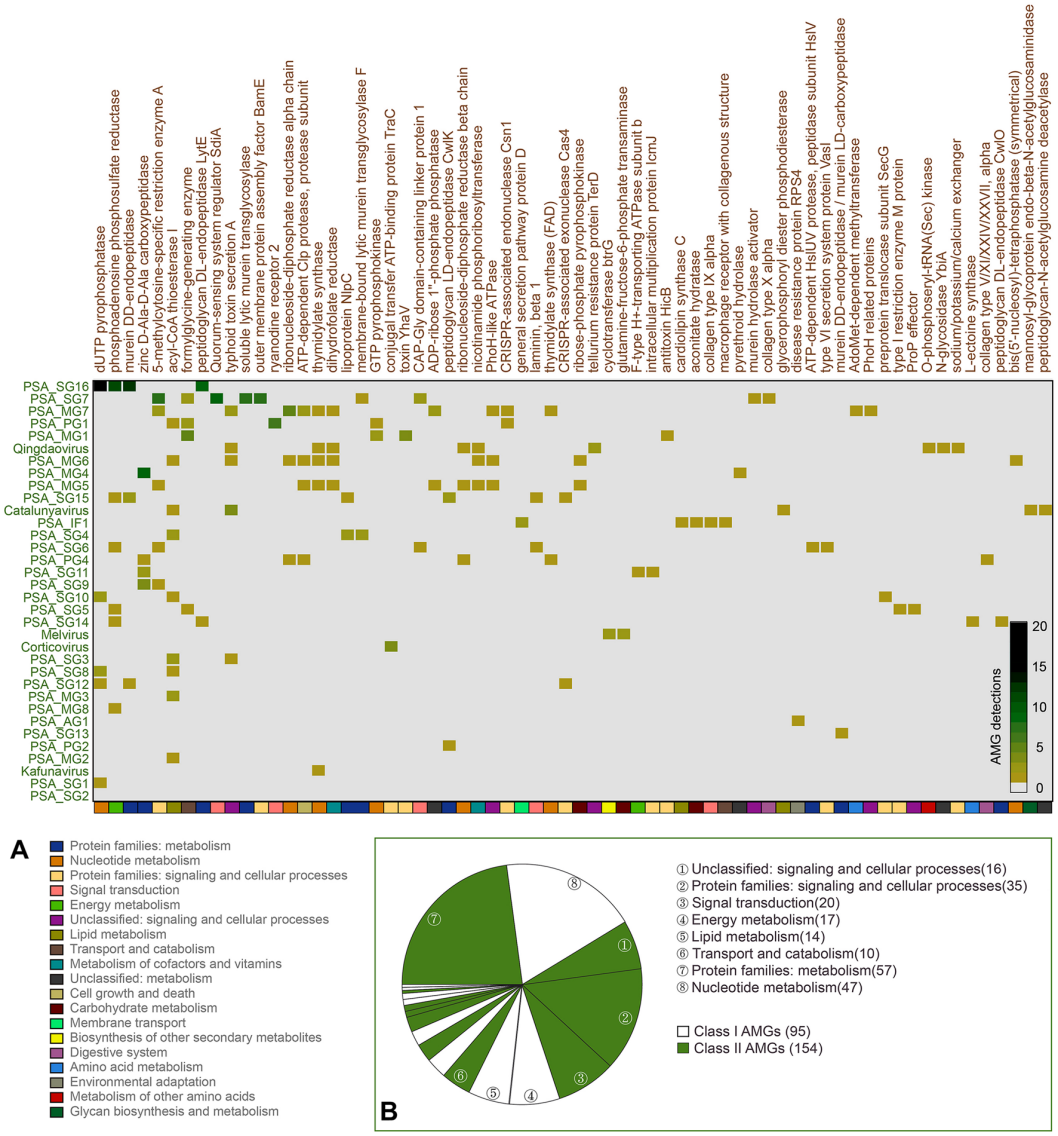
**A** Potential auxiliary metabolism genes (AMGs) encoded by *Pseudoalteromonas* phages from 34 genera and proposed genera, including 239 AMGs belonging to 66 metabolism types. The class I and class II AMGs were identified based on their annotated functions. Different metabolism classifications of AMGs were indicated by different color above the heatmap. The legend of metabolism classifications was shown below the heatmap and was sorted by the number of detections in 19 metabolism classifications. **B** The number of Class I and Class II AMGs was indicated by the pie chart. Top the numbers of detections in 8 metabolism classifications were indicated beside fans

The original article has been corrected.

