# Supplementary material for: Genomic diversity and ecological distribution of marine Pseudoalteromonas phages
Source: Mar Life Sci Technol. 2023 Jan 20;5(2):271–85. doi: 10.1007/s42995-022-00160-z (PMC10232697; doi:10.1007/s42995-022-00160-z)
Supplement: Supplementary file 1 — Supplementary file1 (DOCX 7639 KB) [file 42995_2022_160_MOESM1_ESM.docx]

To: Marine Life Science & Technology

**Supplementary information**

**Genomic diversity and ecological distribution of marine Pseudoalteromonas phages**

Kaiyang Zheng^1†^, Yue Dong ^1†^, Yantao Liang^1,2*^, Yundan Liu^1^, Xinran Zhang^1^, Wenjing Zhang^1^, Ziyue Wang^1^, Hongbing Shao^1,2^, Yeong Yik Sung^2,3^, Wen Jye Mok^2,3^, Li Lian Wong^2,3^, Andrew McMinn^1,4^, Min Wang^1,2,5,6*^

^1^College of Marine Life Sciences, Institute of Evolution and Marine Biodiversity, and Frontiers Science Center for Deep Ocean Multispheres and Earth System, Ocean University of China, Qingdao 266003, China

^2^UMT-OUC Joint Center for Marine Studies, Qingdao 266003, China

^3^Institute of Marine Biotechnology, Universiti Malaysia Terengganu (UMT), Kuala Nerus 21030, Malaysia.

^4^Institute for Marine and Antarctic Studies, University of Tasmania, Hobart, Australia

^5^Haide College, Ocean University of China, Qingdao, China

^6^The Affiliated Hospital of Qingdao University, Qingdao 266000, China.

Running title: Genomes of marine Pseudoalteromonas phages

^†^These authors contributed equally to this work.

^*^Correspondence: Yantao Liang, College of Marine Life Sciences, Ocean University of China, Qingdao 266003, China. Email: [liangyantao@ouc.edu.cn](mailto:liangyantao@ouc.edu.cn)

Min Wang, College of Marine Life Sciences, Ocean University of China, Qingdao 266003, China. Email: [mingwang@ouc.edu.cn](mailto:mingwang@ouc.edu.cn)

Table S1 The information of 144 Pseudoalteromonas-associated phage genomes investigated in this study, including the contig name, GenBank/IMGVR accession number, assembled length, percentage of G+C content, number of open reading frames (ORFs) and tRNA, corresponding VCs assigned by this study, corresponding viral family, source of contig, and corresponding reference. The B8b was excluded in this study due to its fragmented genomes. The data availability URLs of corresponding genomes is listed in the table.

| **Name** | **Accession** | **length** | **GC** | **Number of ORF** | **Number of tRNA** | **VC** | **Taxon** | **Type** | **Reference** | **Links** |
| --- | --- | --- | --- | --- | --- | --- | --- | --- | --- | --- |
| IMGVR_UViG_3300009132_000015 | IMGVR_UViG_3300009132_000015 | 37679 | 48.28 | 48 | 0 | PSA_AG1 | Autographiviridae | UViG | Reported in IMG/VR | https://img.jgi.doe.gov/cgi-bin/vr/main.cgi?section=ViralBrowse&page=uviginfo&uvig_id=IMGVR_UViG_3300009132_000015 |
| IMGVR_UViG_3300027861_000083 | IMGVR_UViG_3300027861_000083 | 37635 | 48.69 | 48 | 0 | PSA_AG1 | Autographiviridae | UViG | Reported in IMG/VR | https://img.jgi.doe.gov/cgi-bin/vr/main.cgi?section=ViralBrowse&page=uviginfo&uvig_id=IMGVR_UViG_3300027861_000083 |
| GXT1010 | MK424903 | 12527 | 43.19 | 25 | 0 | Corticovirus | Corticoviridae | Isolated virus | Xue,C., 2019 | https://www.ncbi.nlm.nih.gov/nuccore/MK424903 |
| Gammaproteobacteria_gi_409167409-274568-285664 | Gammaproteobacteria_gi_409167409-274568-285664 | 11096 | 45.48 | 20 | 0 | Corticovirus | Corticoviridae | Integrated provirus | Reported in IMG/VR | https://www.ncbi.nlm.nih.gov/nuccore/409167409 |
| Cr39582 | NC_042121 | 10584 | 41.9 | 20 | 0 | Corticovirus | Corticoviridae | Isolated virus | Leigh,B.A., et al. 018 | https://www.ncbi.nlm.nih.gov/nuccore/NC_042121 |
| Gammaproteobacteria_gi_484337382-174718-185041 | Gammaproteobacteria_gi_484337382-174718-185041 | 10323 | 45.19 | 19 | 0 | Corticovirus | Corticoviridae | Integrated provirus | Reported in IMG/VR | https://www.ncbi.nlm.nih.gov/nuccore/484337382 |
| PM2 | NC_000867 | 10079 | 42.17 | 20 | 0 | Corticovirus | Corticoviridae | Isolated virus | Mannisto,R.H. et al., 1999 | https://www.ncbi.nlm.nih.gov/nuccore/NC_000867 |
| IMGVR_UViG_2551306111_000001 | IMGVR_UViG_2551306111_000001 | 15442 | 42.91 | 20 | 0 | PSA_IF1 | Inoviridae | Integrated provirus | Reported in IMG/VR | https://img.jgi.doe.gov/cgi-bin/vr/main.cgi?section=ViralBrowse&page=uviginfo&uvig_id=IMGVR_UViG_2551306111_000001 |
| IMGVR_UViG_2728369619_000001 | IMGVR_UViG_2728369619_000001 | 11548 | 40.78 | 15 | 0 | PSA_IF1 | Inoviridae | UViG | Reported in IMG/VR | https://img.jgi.doe.gov/cgi-bin/vr/main.cgi?section=ViralBrowse&page=uviginfo&uvig_id=IMGVR_UViG_2728369619_000001 |
| IMGVR_UViG_2671180867_000001 | IMGVR_UViG_2671180867_000001 | 10352 | 44.44 | 14 | 0 | PSA_IF1 | Inoviridae | Integrated provirus | Reported in IMG/VR | https://img.jgi.doe.gov/cgi-bin/vr/main.cgi?section=ViralBrowse&page=uviginfo&uvig_id=IMGVR_UViG_2671180867_000001 |
| IMGVR_UViG_2505119014_000003 | IMGVR_UViG_2505119014_000003 | 8887 | 41 | 10 | 0 | PSA_IF1 | Inoviridae | Integrated provirus | Reported in IMG/VR | https://img.jgi.doe.gov/cgi-bin/vr/main.cgi?section=ViralBrowse&page=uviginfo&uvig_id=IMGVR_UViG_2505119014_000003 |
| IMGVR_UViG_2551306363_000001 | IMGVR_UViG_2551306363_000001 | 8613 | 44.21 | 14 | 0 | PSA_IF1 | Inoviridae | Integrated provirus | Reported in IMG/VR | https://img.jgi.doe.gov/cgi-bin/vr/main.cgi?section=ViralBrowse&page=uviginfo&uvig_id=IMGVR_UViG_2551306363_000001 |
| Gammaproteobacteria_gi_414071335-1273-11624 | Gammaproteobacteria_gi_414071335-1273-11624 | 8402 | 36.37 | 14 | 0 | PSA_IF1 | Inoviridae | Integrated provirus | Reported in IMG/VR | https://www.ncbi.nlm.nih.gov/nuccore/414071335 |
| IMGVR_UViG_3300025122_001375 | IMGVR_UViG_3300025122_001375 | 8238 | 38.71 | 15 | 0 | PSA_IF1 | Inoviridae | Integrated provirus | Reported in IMG/VR | https://img.jgi.doe.gov/cgi-bin/vr/main.cgi?section=ViralBrowse&page=uviginfo&uvig_id=IMGVR_UViG_3300025122_001375 |
| IMGVR_UViG_3300025125_000289 | IMGVR_UViG_3300025125_000289 | 8238 | 38.71 | 15 | 0 | PSA_IF1 | Inoviridae | Integrated provirus | Reported in IMG/VR | https://img.jgi.doe.gov/cgi-bin/vr/main.cgi?section=ViralBrowse&page=uviginfo&uvig_id=IMGVR_UViG_3300025125_000289 |
| IMGVR_UViG_3300025127_003056 | IMGVR_UViG_3300025127_003056 | 8238 | 38.71 | 15 | 0 | PSA_IF1 | Inoviridae | Integrated provirus | Reported in IMG/VR | https://img.jgi.doe.gov/cgi-bin/vr/main.cgi?section=ViralBrowse&page=uviginfo&uvig_id=IMGVR_UViG_3300025127_003056 |
| IMGVR_UViG_2639763062_000001 | IMGVR_UViG_2639763062_000001 | 8134 | 38.14 | 13 | 0 | PSA_IF1 | Inoviridae | UViG | Reported in IMG/VR | https://img.jgi.doe.gov/cgi-bin/vr/main.cgi?section=ViralBrowse&page=uviginfo&uvig_id=IMGVR_UViG_2639763062_000001 |
| IMGVR_UViG_2576861078_000001 | IMGVR_UViG_2576861078_000001 | 7877 | 36.04 | 12 | 0 | PSA_IF1 | Inoviridae | Integrated provirus | Reported in IMG/VR | https://img.jgi.doe.gov/cgi-bin/vr/main.cgi?section=ViralBrowse&page=uviginfo&uvig_id=IMGVR_UViG_2576861078_000001 |
| IMGVR_UViG_2505119014_000004 | IMGVR_UViG_2505119014_000004 | 7656 | 40.8 | 11 | 0 | PSA_IF1 | Inoviridae | Integrated provirus | Reported in IMG/VR | https://img.jgi.doe.gov/cgi-bin/vr/main.cgi?section=ViralBrowse&page=uviginfo&uvig_id=IMGVR_UViG_2505119014_000004 |
| IMGVR_UViG_2568526121_000001 | IMGVR_UViG_2568526121_000001 | 7267 | 34.64 | 12 | 0 | PSA_IF1 | Inoviridae | Integrated provirus | Reported in IMG/VR | https://img.jgi.doe.gov/cgi-bin/vr/main.cgi?section=ViralBrowse&page=uviginfo&uvig_id=IMGVR_UViG_2568526121_000001 |
| IMGVR_UViG_2579778683_000002 | IMGVR_UViG_2579778683_000002 | 7267 | 34.64 | 12 | 0 | PSA_IF1 | Inoviridae | Integrated provirus | Reported in IMG/VR | https://img.jgi.doe.gov/cgi-bin/vr/main.cgi?section=ViralBrowse&page=uviginfo&uvig_id=IMGVR_UViG_2579778683_000002 |
| IMGVR_UViG_3300026207_000007 | IMGVR_UViG_3300026207_000007 | 6743 | 36.33 | 10 | 0 | PSA_IF1 | Inoviridae | Integrated provirus | Reported in IMG/VR | https://img.jgi.doe.gov/cgi-bin/vr/main.cgi?section=ViralBrowse&page=uviginfo&uvig_id=IMGVR_UViG_3300026207_000007 |
| IMGVR_UViG_2639762812_000001 | IMGVR_UViG_2639762812_000001 | 6738 | 38.63 | 9 | 0 | PSA_IF1 | Inoviridae | Integrated provirus | Reported in IMG/VR | https://img.jgi.doe.gov/cgi-bin/vr/main.cgi?section=ViralBrowse&page=uviginfo&uvig_id=IMGVR_UViG_2639762812_000001 |
| IMGVR_UViG_3300020385_000394 | IMGVR_UViG_3300020385_000394 | 6563 | 36.26 | 10 | 0 | PSA_IF1 | Inoviridae | UViG | Reported in IMG/VR | https://img.jgi.doe.gov/cgi-bin/vr/main.cgi?section=ViralBrowse&page=uviginfo&uvig_id=IMGVR_UViG_3300020385_000394 |
| IMGVR_UViG_3300020388_000441 | IMGVR_UViG_3300020388_000441 | 6563 | 36.26 | 11 | 0 | PSA_IF1 | Inoviridae | UViG | Reported in IMG/VR | https://img.jgi.doe.gov/cgi-bin/vr/main.cgi?section=ViralBrowse&page=uviginfo&uvig_id=IMGVR_UViG_3300020388_000441 |
| IMGVR_UViG_3300020463_001007 | IMGVR_UViG_3300020463_001007 | 6563 | 36.26 | 9 | 0 | PSA_IF1 | Inoviridae | UViG | Reported in IMG/VR | https://img.jgi.doe.gov/cgi-bin/vr/main.cgi?section=ViralBrowse&page=uviginfo&uvig_id=IMGVR_UViG_3300020463_001007 |
| IMGVR_UViG_3300005594_000010 | IMGVR_UViG_3300005594_000010 | 6360 | 36.93 | 9 | 0 | PSA_IF1 | Inoviridae | UViG | Reported in IMG/VR | https://img.jgi.doe.gov/cgi-bin/vr/main.cgi?section=ViralBrowse&page=uviginfo&uvig_id=IMGVR_UViG_3300005594_000010 |
| IMGVR_UViG_3300005567_000001 | IMGVR_UViG_3300005567_000001 | 6350 | 36.93 | 10 | 0 | PSA_IF1 | Inoviridae | UViG | Reported in IMG/VR | https://img.jgi.doe.gov/cgi-bin/vr/main.cgi?section=ViralBrowse&page=uviginfo&uvig_id=IMGVR_UViG_3300005567_000001 |
| IMGVR_UViG_3300005658_000001 | IMGVR_UViG_3300005658_000001 | 6350 | 36.93 | 9 | 0 | PSA_IF1 | Inoviridae | UViG | Reported in IMG/VR | https://img.jgi.doe.gov/cgi-bin/vr/main.cgi?section=ViralBrowse&page=uviginfo&uvig_id=IMGVR_UViG_3300005658_000001 |
| IMGVR_UViG_3300025127_003058 | IMGVR_UViG_3300025127_003058 | 6350 | 36.93 | 10 | 0 | PSA_IF1 | Inoviridae | UViG | Reported in IMG/VR | https://img.jgi.doe.gov/cgi-bin/vr/main.cgi?section=ViralBrowse&page=uviginfo&uvig_id=IMGVR_UViG_3300025127_003058 |
| IMGVR_UViG_3300026190_000025 | IMGVR_UViG_3300026190_000025 | 6350 | 36.93 | 10 | 0 | PSA_IF1 | Inoviridae | UViG | Reported in IMG/VR | https://img.jgi.doe.gov/cgi-bin/vr/main.cgi?section=ViralBrowse&page=uviginfo&uvig_id=IMGVR_UViG_3300026190_000025 |
| IMGVR_UViG_3300026191_000006 | IMGVR_UViG_3300026191_000006 | 6350 | 36.93 | 9 | 0 | PSA_IF1 | Inoviridae | UViG | Reported in IMG/VR | https://img.jgi.doe.gov/cgi-bin/vr/main.cgi?section=ViralBrowse&page=uviginfo&uvig_id=IMGVR_UViG_3300026191_000006 |
| IMGVR_UViG_2728369169_000001 | IMGVR_UViG_2728369169_000001 | 6321 | 39.49 | 10 | 0 | PSA_IF1 | Inoviridae | Integrated provirus | Reported in IMG/VR | https://img.jgi.doe.gov/cgi-bin/vr/main.cgi?section=ViralBrowse&page=uviginfo&uvig_id=IMGVR_UViG_2728369169_000001 |
| RF327 | GU198194 | 6114 | 37.47 | 4 | 0 | PSA_IF1 | Inoviridae | Isolated virus | Yu, Z., et al., 2015 | https://www.ncbi.nlm.nih.gov/nuccore/GU198194 |
| IMGVR_UViG_3300006091_000001 | IMGVR_UViG_3300006091_000001 | 6188 | 37.72 | 10 | 0 | PSA_IF1 | Inoviridae | UViG | Reported in IMG/VR | https://img.jgi.doe.gov/cgi-bin/vr/main.cgi?section=ViralBrowse&page=uviginfo&uvig_id=IMGVR_UViG_3300006091_000001 |
| IMGVR_UViG_3300001679_000286 | IMGVR_UViG_3300001679_000286 | 6069 | 37.63 | 10 | 0 | PSA_IF1 | Inoviridae | UViG | Reported in IMG/VR | https://img.jgi.doe.gov/cgi-bin/vr/main.cgi?section=ViralBrowse&page=uviginfo&uvig_id=IMGVR_UViG_3300001679_000286 |
| IMGVR_UViG_3300001681_000322 | IMGVR_UViG_3300001681_000322 | 6069 | 37.63 | 10 | 0 | PSA_IF1 | Inoviridae | UViG | Reported in IMG/VR | https://img.jgi.doe.gov/cgi-bin/vr/main.cgi?section=ViralBrowse&page=uviginfo&uvig_id=IMGVR_UViG_3300001681_000322 |
| DTR_578141 | DTR_578141 | 6024 | 37.13 | 10 | 0 | PSA_IF1 | Inoviridae | UViG | Reported in IMG/VR | https://portal.nersc.gov/CheckV/checkv-db-v1.0.tar.gz |
| IMGVR_UViG_3300002511_000057 | IMGVR_UViG_3300002511_000057 | 5991 | 36.76 | 9 | 0 | PSA_IF1 | Inoviridae | UViG | Reported in IMG/VR | https://img.jgi.doe.gov/cgi-bin/vr/main.cgi?section=ViralBrowse&page=uviginfo&uvig_id=IMGVR_UViG_3300002511_000057 |
| J2-1 | MF988720 | 142204 | 37.92 | 190 | 17 | Qingdaovirus | Myoviridae | Isolated virus | Liu, Q., 2017 | https://www.ncbi.nlm.nih.gov/nuccore/MF988720 |
| PH357 | KX822733 | 136203 | 34.58 | 262 | 0 | PSA_MG5 | Myoviridae | Isolated virus | Gong, Z., 2016 | https://www.ncbi.nlm.nih.gov/nuccore/KX822733 |
| H101 | NC_029094 | 131903 | 37.36 | 236 | 0 | PSA_MG6 | Myoviridae | Isolated virus | Wang,D.B. and Wang,M., 2015 | https://www.ncbi.nlm.nih.gov/nuccore/NC_029094 |
| HM1 | KF302034 | 129439 | 35.73 | 230 | 2 | PSA_MG7 | Myoviridae | Isolated virus | Duhaime,M.B. and Sullivan,M.B., 2013 | https://www.ncbi.nlm.nih.gov/nuccore/KF302034 |
| SL20 | NC_047839 | 120295 | 35.84 | 194 | 2 | PSA_MG7 | Myoviridae | Isolated virus | Li,H., 2017 | https://www.ncbi.nlm.nih.gov/nuccore/NC_047839 |
| IMGVR_UViG_3300019762_000002 | IMGVR_UViG_3300019762_000002 | 49573 | 45.25 | 75 | 0 | PSA_MG8 | Myoviridae | UViG | Reported in IMG/VR | https://img.jgi.doe.gov/cgi-bin/vr/main.cgi?section=ViralBrowse&page=uviginfo&uvig_id=IMGVR_UViG_3300019762_000002 |
| IMGVR_UViG_3300029448_000067 | IMGVR_UViG_3300029448_000067 | 48818 | 45.42 | 72 | 0 | PSA_MG8 | Myoviridae | UViG | Reported in IMG/VR | https://img.jgi.doe.gov/cgi-bin/vr/main.cgi?section=ViralBrowse&page=uviginfo&uvig_id=IMGVR_UViG_3300029448_000067 |
| Station23_DCM_ALL_assembly_NODE_71_length_48784_cov_8.505264 | Station23_DCM_ALL_assembly_NODE_71 | 48784 | 45.43 | 72 | 0 | PSA_MG8 | Myoviridae | UViG | Reported in IMG/VR | https://datacommons.cyverse.org/browse/iplant/home/shared/iVirus/GOV2.0 |
| DTR_892086 | DTR_892086 | 48729 | 45.42 | 72 | 0 | PSA_MG8 | Myoviridae | UViG | Reported in IMG/VR | https://portal.nersc.gov/CheckV/checkv-db-v1.0.tar.gz |
| IMGVR_UViG_3300010385_000004 | IMGVR_UViG_3300010385_000004 | 45474 | 41.16 | 80 | 0 | PSA_MG3 | Myoviridae | UViG | Reported in IMG/VR | https://img.jgi.doe.gov/cgi-bin/vr/main.cgi?section=ViralBrowse&page=uviginfo&uvig_id=IMGVR_UViG_3300010385_000004 |
| IMGVR_UViG_3300010409_000001 | IMGVR_UViG_3300010409_000001 | 45474 | 41.16 | 81 | 0 | PSA_MG3 | Myoviridae | UViG | Reported in IMG/VR | https://img.jgi.doe.gov/cgi-bin/vr/main.cgi?section=ViralBrowse&page=uviginfo&uvig_id=IMGVR_UViG_3300010409_000001 |
| IMGVR_UViG_3300025122_000031 | IMGVR_UViG_3300025122_000031 | 45289 | 41.72 | 50 | 0 | PSA_MG4 | Myoviridae | UViG | Reported in IMG/VR | https://img.jgi.doe.gov/cgi-bin/vr/main.cgi?section=ViralBrowse&page=uviginfo&uvig_id=IMGVR_UViG_3300025122_000031 |
| IMGVR_UViG_3300025125_000003 | IMGVR_UViG_3300025125_000003 | 45289 | 41.72 | 50 | 0 | PSA_MG4 | Myoviridae | UViG | Reported in IMG/VR | https://img.jgi.doe.gov/cgi-bin/vr/main.cgi?section=ViralBrowse&page=uviginfo&uvig_id=IMGVR_UViG_3300025125_000003 |
| Maelstrom | MG675557 | 45263 | 41.39 | 70 | 0 | PSA_MG2 | Myoviridae | Isolated virus | Haggett,E.F. and Martin,S.E., 2017 | https://www.ncbi.nlm.nih.gov/nuccore/MG675557 |
| IMGVR_UViG_3300002488_000014 | IMGVR_UViG_3300002488_000014 | 45216 | 41.72 | 49 | 0 | PSA_MG4 | Myoviridae | UViG | Reported in IMG/VR | https://img.jgi.doe.gov/cgi-bin/vr/main.cgi?section=ViralBrowse&page=uviginfo&uvig_id=IMGVR_UViG_3300002488_000014 |
| IMGVR_UViG_3300002511_000002 | IMGVR_UViG_3300002511_000002 | 45216 | 41.72 | 49 | 0 | PSA_MG4 | Myoviridae | UViG | Reported in IMG/VR | https://img.jgi.doe.gov/cgi-bin/vr/main.cgi?section=ViralBrowse&page=uviginfo&uvig_id=IMGVR_UViG_3300002511_000002 |
| IMGVR_UViG_3300025132_000076 | IMGVR_UViG_3300025132_000076 | 45212 | 41.72 | 49 | 0 | PSA_MG4 | Myoviridae | UViG | Reported in IMG/VR | https://img.jgi.doe.gov/cgi-bin/vr/main.cgi?section=ViralBrowse&page=uviginfo&uvig_id=IMGVR_UViG_3300025132_000076 |
| IMGVR_UViG_3300005658_000006 | IMGVR_UViG_3300005658_000006 | 40750 | 42.59 | 48 | 0 | PSA_MG4 | Myoviridae | Integrated provirus | Reported in IMG/VR | https://img.jgi.doe.gov/cgi-bin/vr/main.cgi?section=ViralBrowse&page=uviginfo&uvig_id=IMGVR_UViG_3300005658_000006 |
| Station188_DCM_ALL_assembly_NODE_1032_length_40659_cov_11.063639 | Station188_DCM_ALL_assembly_NODE_1032 | 40659 | 41.89 | 54 | 0 | PSA_MG4 | Myoviridae | UViG | Reported in IMG/VR | https://datacommons.cyverse.org/browse/iplant/home/shared/iVirus/GOV2.0 |
| IMGVR_UViG_3300020423_000004 | IMGVR_UViG_3300020423_000004 | 36684 | 42.12 | 48 | 0 | PSA_MG4 | Myoviridae | UViG | Reported in IMG/VR | https://img.jgi.doe.gov/cgi-bin/vr/main.cgi?section=ViralBrowse&page=uviginfo&uvig_id=IMGVR_UViG_3300020423_000004 |
| IMGVR_UViG_3300010883_000156 | IMGVR_UViG_3300010883_000156 | 36078 | 42.11 | 49 | 0 | PSA_MG1 | Myoviridae | UViG | Reported in IMG/VR | https://img.jgi.doe.gov/cgi-bin/vr/main.cgi?section=ViralBrowse&page=uviginfo&uvig_id=IMGVR_UViG_3300010883_000156 |
| IMGVR_UViG_3300001749_000009 | IMGVR_UViG_3300001749_000009 | 36072 | 43.35 | 52 | 0 | Catalunyavirus | Myoviridae | Integrated provirus | Reported in IMG/VR | https://img.jgi.doe.gov/cgi-bin/vr/main.cgi?section=ViralBrowse&page=uviginfo&uvig_id=IMGVR_UViG_3300001749_000009 |
| IMGVR_UViG_3300032273_000003 | IMGVR_UViG_3300032273_000003 | 36006 | 41.95 | 48 | 0 | PSA_MG1 | Myoviridae | Integrated provirus | Reported in IMG/VR | https://img.jgi.doe.gov/cgi-bin/vr/main.cgi?section=ViralBrowse&page=uviginfo&uvig_id=IMGVR_UViG_3300032273_000003 |
| IMGVR_UViG_3300027742_000023 | IMGVR_UViG_3300027742_000023 | 35729 | 43.45 | 53 | 0 | Catalunyavirus | Myoviridae | UViG | Reported in IMG/VR | https://img.jgi.doe.gov/cgi-bin/vr/main.cgi?section=ViralBrowse&page=uviginfo&uvig_id=IMGVR_UViG_3300027742_000023 |
| IMGVR_UViG_3300027372_000005 | IMGVR_UViG_3300027372_000005 | 35455 | 41.38 | 49 | 0 | Catalunyavirus | Myoviridae | UViG | Reported in IMG/VR | https://img.jgi.doe.gov/cgi-bin/vr/main.cgi?section=ViralBrowse&page=uviginfo&uvig_id=IMGVR_UViG_3300027372_000005 |
| IMGVR_UViG_3300020423_000003 | IMGVR_UViG_3300020423_000003 | 35389 | 41.24 | 47 | 0 | Catalunyavirus | Myoviridae | Integrated provirus | Reported in IMG/VR | https://img.jgi.doe.gov/cgi-bin/vr/main.cgi?section=ViralBrowse&page=uviginfo&uvig_id=IMGVR_UViG_3300020423_000003 |
| C5a | NC_047790 | 35209 | 42.23 | 48 | 0 | Catalunyavirus | Myoviridae | Isolated virus | Lara,E., et al., 2016 | https://www.ncbi.nlm.nih.gov/nuccore/NC_047790 |
| Station84_SUR_COMBINED_FINAL_NODE_1140_length_34374_cov_8.039599 | Station84_SUR_COMBINED_FINAL_NODE_1140 | 34374 | 42.59 | 46 | 0 | Catalunyavirus | Myoviridae | UViG | Reported in IMG/VR | https://datacommons.cyverse.org/browse/iplant/home/shared/iVirus/GOV2.0 |
| Station82_SUR_COMBINED_FINAL_NODE_838_length_34176_cov_5.479998 | Station82_SUR_COMBINED_FINAL_NODE_838 | 34176 | 44.17 | 50 | 0 | PSA_MG1 | Myoviridae | UViG | Reported in IMG/VR | https://datacommons.cyverse.org/browse/iplant/home/shared/iVirus/GOV2.0 |
| Station193_SUR_ALL_assembly_NODE_911_length_34006_cov_4.701364 | Station193_SUR_ALL_assembly_NODE_911 | 34006 | 43.78 | 47 | 0 | Catalunyavirus | Myoviridae | UViG | Reported in IMG/VR | https://datacommons.cyverse.org/browse/iplant/home/shared/iVirus/GOV2.0 |
| Station70_MES_COMBINED_FINAL_NODE_1684_length_33645_cov_19.879696 | Station70_MES_COMBINED_FINAL_NODE_1684 | 33645 | 43.55 | 49 | 0 | Catalunyavirus | Myoviridae | UViG | Reported in IMG/VR | https://datacommons.cyverse.org/browse/iplant/home/shared/iVirus/GOV2.0 |
| Gammaproteobacteria_gi_414071926 | Gammaproteobacteria_gi_414071926 | 33392 | 41.79 | 46 | 0 | PSA_MG1 | Myoviridae | Integrated provirus | Reported in IMG/VR | https://www.ncbi.nlm.nih.gov/nuccore/414071926 |
| Station85_SUR_COMBINED_FINAL_NODE_902_length_32977_cov_12.500243 | Station85_SUR_COMBINED_FINAL_NODE_902 | 32977 | 41.9 | 46 | 0 | Catalunyavirus | Myoviridae | UViG | Reported in IMG/VR | https://datacommons.cyverse.org/browse/iplant/home/shared/iVirus/GOV2.0 |
| Station178_SUR_ALL_assembly_NODE_2136_length_32919_cov_6.431019 | Station178_SUR_ALL_assembly_NODE_2136 | 32919 | 43.17 | 46 | 0 | Catalunyavirus | Myoviridae | UViG | Reported in IMG/VR | https://datacommons.cyverse.org/browse/iplant/home/shared/iVirus/GOV2.0 |
| IMGVR_UViG_3300026207_000002 | IMGVR_UViG_3300026207_000002 | 32382 | 43.56 | 44 | 0 | PSA_MG1 | Myoviridae | Integrated provirus | Reported in IMG/VR | https://img.jgi.doe.gov/cgi-bin/vr/main.cgi?section=ViralBrowse&page=uviginfo&uvig_id=IMGVR_UViG_3300026207_000002 |
| pYD6-A | NC_020849 | 76802 | 38.66 | 105 | 3 | PSA_PG4 | Podoviridae | Isolated virus | Henn,M.R., et al., 2013 | https://www.ncbi.nlm.nih.gov/nuccore/NC_020849 |
| DTR_892308 | DTR_892308 | 58039 | 40.61 | 69 | 0 | PSA_PG1 | Podoviridae | UViG | Reported in IMG/VR | https://portal.nersc.gov/CheckV/checkv-db-v1.0.tar.gz |
| DTR_577845 | DTR_577845 | 57292 | 43.55 | 63 | 0 | PSA_PG1 | Podoviridae | UViG | Reported in IMG/VR | https://portal.nersc.gov/CheckV/checkv-db-v1.0.tar.gz |
| IMGVR_UViG_3300020385_000019 | IMGVR_UViG_3300020385_000019 | 55979 | 43.51 | 71 | 0 | PSA_PG1 | Podoviridae | UViG | Reported in IMG/VR | https://img.jgi.doe.gov/cgi-bin/vr/main.cgi?section=ViralBrowse&page=uviginfo&uvig_id=IMGVR_UViG_3300020385_000019 |
| Station205_SUR_ALL_assembly_NODE_403_length_55434_cov_32.917568 | Station205_SUR_ALL_assembly_NODE_403 | 55434 | 42.78 | 72 | 0 | PSA_PG1 | Podoviridae | UViG | Reported in IMG/VR | https://datacommons.cyverse.org/browse/iplant/home/shared/iVirus/GOV2.0 |
| IMGVR_UViG_3300025191_000002 | IMGVR_UViG_3300025191_000002 | 55260 | 43.46 | 69 | 0 | PSA_PG1 | Podoviridae | UViG | Reported in IMG/VR | https://img.jgi.doe.gov/cgi-bin/vr/main.cgi?section=ViralBrowse&page=uviginfo&uvig_id=IMGVR_UViG_3300025191_000002 |
| IMGVR_UViG_3300009428_000011 | IMGVR_UViG_3300009428_000011 | 53852 | 43.12 | 65 | 0 | PSA_PG1 | Podoviridae | UViG | Reported in IMG/VR | https://img.jgi.doe.gov/cgi-bin/vr/main.cgi?section=ViralBrowse&page=uviginfo&uvig_id=IMGVR_UViG_3300009428_000011 |
| PH1 | NC_031908 | 42685 | 42.24 | 54 | 0 | Kafunavirus | Podoviridae | Isolated virus | Liu,Z. 2016 | https://www.ncbi.nlm.nih.gov/nuccore/NC_031908 |
| vB_PspS-H6/1 | KX257490 | 36753 | 45.03 | 59 | 0 | PSA_PG2 | Podoviridae | Isolated virus | Kallies,R., 2018 | https://www.ncbi.nlm.nih.gov/nuccore/KX257490 |
| HP1 | NC_048630 | 45035 | 44.67 | 59 | 0 | Melvirus | Zobellviridae | Isolated virus | Duhaime,M.B. and Sullivan,M.B., 2013 | https://www.ncbi.nlm.nih.gov/nuccore/NC_048630 |
| RIO-1 | NC_021300 | 43882 | 44.72 | 58 | 0 | Melvirus | Zobellviridae | Isolated virus | Hardies,S.C., et al., 2013 | https://www.ncbi.nlm.nih.gov/nuccore/NC_021300 |
| KB12-38 | MF098558 | 78271 | 42.22 | 87 | 0 | PSA_SG5 | Siphoviridae | Isolated virus | Zablocki,O.D.J., et al., 2017 | https://www.ncbi.nlm.nih.gov/nuccore/MF098558 |
| Station173_DCM_ALL_assembly_NODE_1367_length_51271_cov_6.636286 | Station173_DCM_ALL_assembly_NODE_1367 | 51271 | 42.76 | 86 | 0 | PSA_SG7 | Siphoviridae | UViG | Reported in IMG/VR | https://datacommons.cyverse.org/browse/iplant/home/shared/iVirus/GOV2.0 |
| DTR_890449 | DTR_890449 | 51214 | 42.75 | 86 | 0 | PSA_SG7 | Siphoviridae | UViG | Reported in IMG/VR | https://portal.nersc.gov/CheckV/checkv-db-v1.0.tar.gz |
| IMGVR_UViG_3300020473_000002 | IMGVR_UViG_3300020473_000002 | 51011 | 44.48 | 81 | 0 | PSA_SG7 | Siphoviridae | UViG | Reported in IMG/VR | https://img.jgi.doe.gov/cgi-bin/vr/main.cgi?section=ViralBrowse&page=uviginfo&uvig_id=IMGVR_UViG_3300020473_000002 |
| IMGVR_UViG_3300032360_000014 | IMGVR_UViG_3300032360_000014 | 50253 | 44.33 | 72 | 0 | PSA_SG6 | Siphoviridae | UViG | Reported in IMG/VR | https://img.jgi.doe.gov/cgi-bin/vr/main.cgi?section=ViralBrowse&page=uviginfo&uvig_id=IMGVR_UViG_3300032360_000014 |
| IMGVR_UViG_3300031660_000008 | IMGVR_UViG_3300031660_000008 | 49598 | 43.05 | 77 | 0 | PSA_SG7 | Siphoviridae | UViG | Reported in IMG/VR | https://img.jgi.doe.gov/cgi-bin/vr/main.cgi?section=ViralBrowse&page=uviginfo&uvig_id=IMGVR_UViG_3300031660_000008 |
| Station85_SUR_COMBINED_FINAL_NODE_542_length_49541_cov_13.601200 | Station85_SUR_COMBINED_FINAL_NODE_542 | 49541 | 43.39 | 76 | 0 | PSA_SG7 | Siphoviridae | UViG | Reported in IMG/VR | https://datacommons.cyverse.org/browse/iplant/home/shared/iVirus/GOV2.0 |
| IMGVR_UViG_3300031696_000009 | IMGVR_UViG_3300031696_000009 | 49504 | 43.03 | 76 | 0 | PSA_SG7 | Siphoviridae | UViG | Reported in IMG/VR | https://img.jgi.doe.gov/cgi-bin/vr/main.cgi?section=ViralBrowse&page=uviginfo&uvig_id=IMGVR_UViG_3300031696_000009 |
| DTR_893818 | DTR_893818 | 49488 | 43.39 | 76 | 0 | PSA_SG7 | Siphoviridae | UViG | Reported in IMG/VR | https://portal.nersc.gov/CheckV/checkv-db-v1.0.tar.gz |
| XC | MT002874 | 46609 | 40.01 | 76 | 1 | PSA_SG15 | Siphoviridae | Isolated virus | Zhang,X., 2020 | https://www.ncbi.nlm.nih.gov/nuccore/MT002874 |
| IMGVR_UViG_3300020369_000005 | IMGVR_UViG_3300020369_000005 | 46522 | 44.72 | 74 | 0 | PSA_SG7 | Siphoviridae | UViG | Reported in IMG/VR | https://img.jgi.doe.gov/cgi-bin/vr/main.cgi?section=ViralBrowse&page=uviginfo&uvig_id=IMGVR_UViG_3300020369_000005 |
| XCL1123 | MN313256 | 45773 | 40.65 | 84 | 0 | PSA_SG14 | Siphoviridae | Isolated virus | Xue, C., 2019 | https://www.ncbi.nlm.nih.gov/nuccore/MN313256 |
| Station137_DCM_ALL_assembly_NODE_68_length_45654_cov_53.904998 | Station137_DCM_ALL_assembly_NODE_68 | 45654 | 39.53 | 91 | 1 | PSA_SG15 | Siphoviridae | UViG | Reported in IMG/VR | https://datacommons.cyverse.org/browse/iplant/home/shared/iVirus/GOV2.0 |
| DTR_889654 | DTR_889654 | 45599 | 39.52 | 91 | 1 | PSA_SG15 | Siphoviridae | UViG | Reported in IMG/VR | https://portal.nersc.gov/CheckV/checkv-db-v1.0.tar.gz |
| vB_PspS-H40/1 | KU747973 | 45306 | 40.15 | 73 | 0 | PSA_SG15 | Siphoviridae | Isolated virus | Kallies,R., 2017 | https://www.ncbi.nlm.nih.gov/nuccore/KU747973 |
| IMGVR_UViG_3300027828_000047 | IMGVR_UViG_3300027828_000047 | 44387 | 39.55 | 76 | 0 | PSA_SG16 | Siphoviridae | UViG | Reported in IMG/VR | https://img.jgi.doe.gov/cgi-bin/vr/main.cgi?section=ViralBrowse&page=uviginfo&uvig_id=IMGVR_UViG_3300027828_000047 |
| IMGVR_UViG_3300027980_000047 | IMGVR_UViG_3300027980_000047 | 44381 | 39.53 | 76 | 0 | PSA_SG16 | Siphoviridae | UViG | Reported in IMG/VR | https://img.jgi.doe.gov/cgi-bin/vr/main.cgi?section=ViralBrowse&page=uviginfo&uvig_id=IMGVR_UViG_3300027980_000047 |
| IMGVR_UViG_3300005588_000013 | IMGVR_UViG_3300005588_000013 | 44253 | 39.55 | 76 | 0 | PSA_SG16 | Siphoviridae | UViG | Reported in IMG/VR | https://img.jgi.doe.gov/cgi-bin/vr/main.cgi?section=ViralBrowse&page=uviginfo&uvig_id=IMGVR_UViG_3300005588_000013 |
| IMGVR_UViG_3300005589_000008 | IMGVR_UViG_3300005589_000008 | 44253 | 39.55 | 75 | 0 | PSA_SG16 | Siphoviridae | UViG | Reported in IMG/VR | https://img.jgi.doe.gov/cgi-bin/vr/main.cgi?section=ViralBrowse&page=uviginfo&uvig_id=IMGVR_UViG_3300005589_000008 |
| IMGVR_UViG_3300005609_000001 | IMGVR_UViG_3300005609_000001 | 44253 | 39.55 | 76 | 0 | PSA_SG16 | Siphoviridae | UViG | Reported in IMG/VR | https://img.jgi.doe.gov/cgi-bin/vr/main.cgi?section=ViralBrowse&page=uviginfo&uvig_id=IMGVR_UViG_3300005609_000001 |
| IMGVR_UViG_3300005920_000002 | IMGVR_UViG_3300005920_000002 | 44253 | 39.55 | 76 | 0 | PSA_SG16 | Siphoviridae | UViG | Reported in IMG/VR | https://img.jgi.doe.gov/cgi-bin/vr/main.cgi?section=ViralBrowse&page=uviginfo&uvig_id=IMGVR_UViG_3300005920_000002 |
| IMGVR_UViG_3300006467_000168 | IMGVR_UViG_3300006467_000168 | 44253 | 39.55 | 76 | 0 | PSA_SG16 | Siphoviridae | UViG | Reported in IMG/VR | https://img.jgi.doe.gov/cgi-bin/vr/main.cgi?section=ViralBrowse&page=uviginfo&uvig_id=IMGVR_UViG_3300006467_000168 |
| IMGVR_UViG_3300010392_000285 | IMGVR_UViG_3300010392_000285 | 44253 | 39.55 | 76 | 0 | PSA_SG16 | Siphoviridae | UViG | Reported in IMG/VR | https://img.jgi.doe.gov/cgi-bin/vr/main.cgi?section=ViralBrowse&page=uviginfo&uvig_id=IMGVR_UViG_3300010392_000285 |
| IMGVR_UViG_3300010430_000118 | IMGVR_UViG_3300010430_000118 | 44253 | 39.55 | 76 | 0 | PSA_SG16 | Siphoviridae | UViG | Reported in IMG/VR | https://img.jgi.doe.gov/cgi-bin/vr/main.cgi?section=ViralBrowse&page=uviginfo&uvig_id=IMGVR_UViG_3300010430_000118 |
| IMGVR_UViG_3300027758_000002 | IMGVR_UViG_3300027758_000002 | 44253 | 39.55 | 75 | 0 | PSA_SG16 | Siphoviridae | UViG | Reported in IMG/VR | https://img.jgi.doe.gov/cgi-bin/vr/main.cgi?section=ViralBrowse&page=uviginfo&uvig_id=IMGVR_UViG_3300027758_000002 |
| IMGVR_UViG_3300027967_000003 | IMGVR_UViG_3300027967_000003 | 44253 | 39.55 | 76 | 0 | PSA_SG16 | Siphoviridae | UViG | Reported in IMG/VR | https://img.jgi.doe.gov/cgi-bin/vr/main.cgi?section=ViralBrowse&page=uviginfo&uvig_id=IMGVR_UViG_3300027967_000003 |
| H103 | NC_028819 | 43190 | 41.17 | 77 | 0 | PSA_SG14 | Siphoviridae | Isolated virus | Sun,M.-Q., et al., 2015 | https://www.ncbi.nlm.nih.gov/nuccore/NC_028819 |
| Station158_SUR_ALL_assembly_NODE_1716_length_42512_cov_261.928398 | Station158_SUR_ALL_assembly_NODE_1716 | 42512 | 39.47 | 86 | 0 | PSA_SG16 | Siphoviridae | UViG | Reported in IMG/VR | https://datacommons.cyverse.org/browse/iplant/home/shared/iVirus/GOV2.0 |
| DTR_890239 | DTR_890239 | 42457 | 39.47 | 86 | 0 | PSA_SG16 | Siphoviridae | UViG | Reported in IMG/VR | https://portal.nersc.gov/CheckV/checkv-db-v1.0.tar.gz |
| C7 | MK387309 | 42261 | 40.63 | 72 | 1 | PSA_SG12 | Siphoviridae | Isolated virus | Wang, Q., 2019 | https://www.ncbi.nlm.nih.gov/nuccore/MK387309 |
| Station137_DCM_ALL_assembly_NODE_84_length_41860_cov_826.478436 | Station137_DCM_ALL_assembly_NODE_84 | 41860 | 39.21 | 79 | 0 | PSA_SG16 | Siphoviridae | UViG | Reported in IMG/VR | https://datacommons.cyverse.org/browse/iplant/home/shared/iVirus/GOV2.0 |
| Station137_MES_COMBINED_FINAL_NODE_1181_length_41860_cov_648.569597 | Station137_MES_COMBINED_FINAL_NODE_1181 | 41860 | 39.21 | 79 | 0 | PSA_SG16 | Siphoviridae | UViG | Reported in IMG/VR | https://datacommons.cyverse.org/browse/iplant/home/shared/iVirus/GOV2.0 |
| Station137_MES_DO_NOT_POOL_NODE_1181_length_41860_cov_564.048176 | Station137_MES_DO_NOT_POOL_NODE_1181 | 41860 | 39.21 | 79 | 0 | PSA_SG16 | Siphoviridae | UViG | Reported in IMG/VR | https://datacommons.cyverse.org/browse/iplant/home/shared/iVirus/GOV2.0 |
| Station137_SUR_ALL_assembly_NODE_95_length_41860_cov_1046.739983 | Station137_SUR_ALL_assembly_NODE_95 | 41860 | 39.21 | 79 | 0 | PSA_SG16 | Siphoviridae | UViG | Reported in IMG/VR | https://datacommons.cyverse.org/browse/iplant/home/shared/iVirus/GOV2.0 |
| Station138_SUR_ALL_assembly_NODE_88_length_41860_cov_10.422557 | Station138_SUR_ALL_assembly_NODE_88 | 41860 | 39.21 | 79 | 0 | PSA_SG16 | Siphoviridae | UViG | Reported in IMG/VR | https://datacommons.cyverse.org/browse/iplant/home/shared/iVirus/GOV2.0 |
| Station85_MES_COMBINED_FINAL_NODE_626_length_41860_cov_9.397560 | Station85_MES_COMBINED_FINAL_NODE_626 | 41860 | 39.21 | 79 | 0 | PSA_SG16 | Siphoviridae | UViG | Reported in IMG/VR | https://datacommons.cyverse.org/browse/iplant/home/shared/iVirus/GOV2.0 |
| DTR_889761 | DTR_889761 | 41805 | 39.21 | 79 | 0 | PSA_SG16 | Siphoviridae | UViG | Reported in IMG/VR | https://portal.nersc.gov/CheckV/checkv-db-v1.0.tar.gz |
| Station78_MES_COMBINED_FINAL_NODE_1040_length_40570_cov_7.922695 | Station78_MES_COMBINED_FINAL_NODE_1040 | 40570 | 39.94 | 73 | 0 | PSA_SG11 | Siphoviridae | UViG | Reported in IMG/VR | https://datacommons.cyverse.org/browse/iplant/home/shared/iVirus/GOV2.0 |
| BS5 | NC_031917 | 39949 | 40.6 | 69 | 0 | PSA_SG10 | Siphoviridae | Isolated virus | Meng,X., 2016 | https://www.ncbi.nlm.nih.gov/nuccore/NC_031917 |
| TW1 | KC542353 | 39940 | 40.19 | 66 | 0 | PSA_SG13 | Siphoviridae | Isolated virus | Lee,J.-H., et al., 2013 | https://www.ncbi.nlm.nih.gov/nuccore/KC542353 |
| Station82_SUR_COMBINED_FINAL_NODE_658_length_39824_cov_1556.026855 | Station82_SUR_COMBINED_FINAL_NODE_658 | 39824 | 40.23 | 52 | 0 | PSA_SG9 | Siphoviridae | UViG | Reported in IMG/VR | https://datacommons.cyverse.org/browse/iplant/home/shared/iVirus/GOV2.0 |
| DTR_577734 | DTR_577734 | 39460 | 39.7 | 74 | 0 | PSA_SG11 | Siphoviridae | UViG | Reported in IMG/VR | https://portal.nersc.gov/CheckV/checkv-db-v1.0.tar.gz |
| HS2 | KF302036 | 38208 | 40.23 | 72 | 0 | PSA_SG10 | Siphoviridae | Isolated virus | Duhaime,M.B. and Sullivan,M.B., 2013 | https://www.ncbi.nlm.nih.gov/nuccore/KF302036 |
| HS5 | KF302032 | 37730 | 40.4 | 64 | 0 | PSA_SG9 | Siphoviridae | Isolated virus | Duhaime,M.B. and Sullivan,M.B., 2013 | https://www.ncbi.nlm.nih.gov/nuccore/KF302032 |
| HS1 | KF302033 | 37211 | 40.53 | 66 | 0 | PSA_SG9 | Siphoviridae | Isolated virus | Duhaime,M.B. and Sullivan,M.B., 2013 | https://www.ncbi.nlm.nih.gov/nuccore/KF302033 |
| Gammaproteobacteria_gi_409167369 | Gammaproteobacteria_gi_409167369 | 36044 | 45.83 | 51 | 1 | PSA_SG2 | Siphoviridae | UViG | Reported in IMG/VR | https://www.ncbi.nlm.nih.gov/nuccore/409167369 |
| PHS21 | KY379511 | 35802 | 40.63 | 55 | 0 | PSA_SG8 | Siphoviridae | Isolated virus | Li,H., 2017 | https://www.ncbi.nlm.nih.gov/nuccore/KY379511 |
| PHS3 | KX912252 | 35626 | 40.85 | 64 | 0 | PSA_SG8 | Siphoviridae | Isolated virus | Li,H., 2016 | https://www.ncbi.nlm.nih.gov/nuccore/KX912252 |
| Kang_2014-00036_D_NODE_6_length_35471_cov_40.203665 | Kang_2014-00036_D_NODE_6 | 35471 | 44.91 | 52 | 1 | PSA_SG3 | Siphoviridae | UViG | Reported in IMG/VR | https://www.ncbi.nlm.nih.gov/nuccore/Kang_2014-00036_D_NODE_6 |
| HS6 | KF302035 | 35330 | 44.87 | 53 | 0 | PSA_SG3 | Siphoviridae | Isolated virus | Duhaime,M.B. and Sullivan,M.B., 2013 | https://www.ncbi.nlm.nih.gov/nuccore/KF302035 |
| SL25 | MF370965 | 33906 | 40.67 | 61 | 0 | PSA_SG8 | Siphoviridae | Isolated virus | Liu, Z., 2017 | https://www.ncbi.nlm.nih.gov/nuccore/MF370965 |
| AL | MT002875 | 33582 | 40.08 | 53 | 0 | PSA_SG8 | Siphoviridae | Isolated virus | Zhang,X., 2020 | https://www.ncbi.nlm.nih.gov/nuccore/MT002875 |
| Pq0 | NC_029100 | 33399 | 40.29 | 58 | 2 | PSA_SG8 | Siphoviridae | Isolated virus | Wang,D.B. and Wang,M., 2015 | https://www.ncbi.nlm.nih.gov/nuccore/NC_029100 |
| IMGVR_UViG_3300002484_000004 | IMGVR_UViG_3300002484_000004 | 32568 | 45.6 | 47 | 0 | PSA_SG3 | Siphoviridae | Integrated provirus | Reported in IMG/VR | https://img.jgi.doe.gov/cgi-bin/vr/main.cgi?section=ViralBrowse&page=uviginfo&uvig_id=IMGVR_UViG_3300002484_000004 |
| H105/1 | NC_015293 | 30651 | 40.82 | 51 | 0 | PSA_SG9 | Siphoviridae | Isolated virus | Duhaime,M.B., et al., 2010 | https://www.ncbi.nlm.nih.gov/nuccore/NC_015293 |
| B8b | KJ944830 |  |  |  |  |  | Siphoviridae | Isolated virus | Lara,E., et al., 2015 | https://www.ncbi.nlm.nih.gov/nuccore/KJ944830 |
| Station76_MES_COMBINED_FINAL_NODE_1001_length_48270_cov_18.343254 | Station76_MES_COMBINED_FINAL_NODE_1001 | 48270 | 42.53 | 64 | 0 | PSA_SG1 | Siphoviridae | UViG | Reported in IMG/VR | https://datacommons.cyverse.org/browse/iplant/home/shared/iVirus/GOV2.0 |
| Station201_DCM_ALL_assembly_NODE_823_length_41192_cov_31.076744 | Station201_DCM_ALL_assembly_NODE_823 | 41192 | 41.7 | 60 | 0 | PSA_SG4 | Siphoviridae | UViG | Reported in IMG/VR | https://datacommons.cyverse.org/browse/iplant/home/shared/iVirus/GOV2.0 |
| DTR_891517 | DTR_891517 | 41144 | 41.69 | 60 | 0 | PSA_SG4 | Siphoviridae | UViG | Reported in IMG/VR | https://portal.nersc.gov/CheckV/checkv-db-v1.0.tar.gz |

Table S2 The information about the 249 auxiliary metabolism genes (AMGs) detected based on the annotation against KEGG Orthologs (KO) database, including corresponding VC, contig ID, open reading frames (ORFs) ID, KO accession, E-value, KO definition, and AMG classification.

| **VC** | **genome acc** | **gene id** | **KO** | **E-value** | **KO definition** | **Classification** |
| --- | --- | --- | --- | --- | --- | --- |
| PSA_SG11 | DTR_577734 | DTR_577734\|_49 | K02109 | 3.8E-06 | F-type H+-transporting ATPase subunit b | AMGI |
| PSA_SG11 | DTR_577734 | DTR_577734\|_4 | K08640 | 6.40E-20 | zinc D-Ala-D-Ala carboxypeptidase [EC:3.4.17.14] | AMGII |
| PSA_PG1 | DTR_577845 | DTR_577845\|_44 | K04962 | 5.90E-11 | ryanodine receptor 2 | AMGII |
| PSA_PG1 | DTR_577845 | DTR_577845\|_51 | K13444 | 3.60E-11 | formylglycine-generating enzyme [EC:1.8.3.7] | AMGII |
| PSA_SG15 | DTR_889654 | DTR_889654\|_27 | K19303 | 5.60E-09 | murein DD-endopeptidase [EC:3.4.-.-] | AMGII |
| PSA_SG16 | DTR_889761 | DTR_889761\|_63 | K01520 | 1.70E-35 | dUTP pyrophosphatase [EC:3.6.1.23] | AMGI |
| PSA_SG16 | DTR_889761 | DTR_889761\|_78 | K19224 | 0.000002 | peptidoglycan DL-endopeptidase LytE [EC:3.4.-.-] | AMGII |
| PSA_SG16 | DTR_890239 | DTR_890239\|_35 | K01520 | 3.60E-33 | dUTP pyrophosphatase [EC:3.6.1.23] | AMGI |
| PSA_SG16 | DTR_890239 | DTR_890239\|_54 | K19303 | 2.8E-06 | murein DD-endopeptidase [EC:3.4.-.-] | AMGII |
| PSA_SG7 | DTR_890449 | DTR_890449\|_8 | K07451 | 2E-07 | 5-methylcytosine-specific restriction enzyme A [EC:3.1.21.-] | AMGII |
| PSA_SG7 | DTR_890449 | DTR_890449\|_13 | K08309 | 1.00E-23 | soluble lytic murein transglycosylase [EC:4.2.2.-] | AMGII |
| PSA_SG7 | DTR_890449 | DTR_890449\|_31 | K07782 | 3.6E-08 | LuxR family transcriptional regulator, quorum-sensing system regulator SdiA | AMGII |
| PSA_SG7 | DTR_890449 | DTR_890449\|_64 | K06186 | 0.000006 | outer membrane protein assembly factor BamE | AMGII |
| PSA_SG4 | DTR_891517 | DTR_891517\|_30 | K10804 | 1.7E-08 | acyl-CoA thioesterase I [EC:3.1.2.- 3.1.2.2 3.1.1.2 3.1.1.5] | AMGI |
| PSA_SG4 | DTR_891517 | DTR_891517\|_9 | K13695 | 8.6E-07 | probable lipoprotein NlpC | AMGII |
| PSA_SG4 | DTR_891517 | DTR_891517\|_33 | K18691 | 2.50E-28 | membrane-bound lytic murein transglycosylase F [EC:4.2.2.-] | AMGII |
| PSA_PG1 | DTR_892308 | DTR_892308\|_40 | K07816 | 1.10E-27 | putative GTP pyrophosphokinase [EC:2.7.6.5] | AMGI |
| PSA_PG1 | DTR_892308 | DTR_892308\|_39 | K09952 | 7.8E-06 | CRISPR-associated endonuclease Csn1 [EC:3.1.-.-] | AMGII |
| PSA_PG1 | DTR_892308 | DTR_892308\|_61 | K04962 | 1.50E-10 | ryanodine receptor 2 | AMGII |
| PSA_SG7 | DTR_893818 | DTR_893818\|_8 | K07451 | 2.6E-07 | 5-methylcytosine-specific restriction enzyme A [EC:3.1.21.-] | AMGII |
| PSA_SG7 | DTR_893818 | DTR_893818\|_11 | K08309 | 1.20E-23 | soluble lytic murein transglycosylase [EC:4.2.2.-] | AMGII |
| PSA_SG7 | DTR_893818 | DTR_893818\|_27 | K07782 | 3.6E-08 | LuxR family transcriptional regulator, quorum-sensing system regulator SdiA | AMGII |
| PSA_SG7 | DTR_893818 | DTR_893818\|_50 | K06186 | 7.3E-06 | outer membrane protein assembly factor BamE | AMGII |
| PSA_SG7 | DTR_893818 | DTR_893818\|_62 | K13444 | 5.50E-13 | formylglycine-generating enzyme [EC:1.8.3.7] | AMGII |
| Corticovirus | Gammaproteobacteria_gi_409167409-274568-285664 | Gammaproteobacteria_gi_409167409-274568-285664\|_14 | K12063 | 3.10E-09 | conjugal transfer ATP-binding protein TraC | AMGII |
| PSA_MG1 | Gammaproteobacteria_gi_414071926 | Gammaproteobacteria_gi_414071926\|_6 | K13444 | 1.10E-11 | formylglycine-generating enzyme [EC:1.8.3.7] | AMGII |
| PSA_MG1 | Gammaproteobacteria_gi_414071926 | Gammaproteobacteria_gi_414071926\|_31 | K18843 | 4.80E-14 | antitoxin HicB | AMGII |
| Corticovirus | Gammaproteobacteria_gi_484337382-174718-185041 | Gammaproteobacteria_gi_484337382-174718-185041\|_5 | K12063 | 3.10E-09 | conjugal transfer ATP-binding protein TraC | AMGII |
| PSA_IF1 | IMGVR_UViG_2505119014_000003 | IMGVR_UViG_2505119014_000003\|_9 | K02453 | 1.60E-75 | general secretion pathway protein D | AMGII |
| PSA_IF1 | IMGVR_UViG_2505119014_000004 | IMGVR_UViG_2505119014_000004\|_2 | K02453 | 1.60E-75 | general secretion pathway protein D | AMGII |
| PSA_IF1 | IMGVR_UViG_2551306111_000001 | IMGVR_UViG_2551306111_000001\|_5 | K06132 | 1.80E-09 | cardiolipin synthase C [EC:2.7.8.-] | AMGI |
| PSA_IF1 | IMGVR_UViG_2551306111_000001 | IMGVR_UViG_2551306111_000001\|_18 | K01681 | 9.6E-06 | aconitate hydratase [EC:4.2.1.3] | AMGI |
| PSA_IF1 | IMGVR_UViG_2671180867_000001 | IMGVR_UViG_2671180867_000001\|_4 | K08131 | 1.40E-10 | collagen type IX alpha | AMGII |
| PSA_IF1 | IMGVR_UViG_2728369169_000001 | IMGVR_UViG_2728369169_000001\|_3 | K13884 | 4.90E-23 | macrophage receptor with collagenous structure | AMGII |
| PSA_SG3 | IMGVR_UViG_3300002484_000004 | IMGVR_UViG_3300002484_000004\|_17 | K22918 | 2.8E-06 | typhoid toxin secretion A | AMGII |
| PSA_MG4 | IMGVR_UViG_3300002488_000014 | IMGVR_UViG_3300002488_000014\|_10 | K08640 | 1.8E-08 | zinc D-Ala-D-Ala carboxypeptidase [EC:3.4.17.14] | AMGII |
| PSA_MG4 | IMGVR_UViG_3300002511_000002 | IMGVR_UViG_3300002511_000002\|_40 | K08640 | 1.8E-08 | zinc D-Ala-D-Ala carboxypeptidase [EC:3.4.17.14] | AMGII |
| PSA_SG16 | IMGVR_UViG_3300005588_000013 | IMGVR_UViG_3300005588_000013\|_43 | K01520 | 7.30E-35 | dUTP pyrophosphatase [EC:3.6.1.23] | AMGI |
| PSA_SG16 | IMGVR_UViG_3300005588_000013 | IMGVR_UViG_3300005588_000013\|_65 | K00390 | 2.7E-07 | phosphoadenosine phosphosulfate reductase [EC:1.8.4.8 1.8.4.10] | AMGI |
| PSA_SG16 | IMGVR_UViG_3300005588_000013 | IMGVR_UViG_3300005588_000013\|_28 | K19303 | 1.10E-11 | murein DD-endopeptidase [EC:3.4.-.-] | AMGII |
| PSA_SG16 | IMGVR_UViG_3300005589_000008 | IMGVR_UViG_3300005589_000008\|_28 | K00390 | 2.7E-07 | phosphoadenosine phosphosulfate reductase [EC:1.8.4.8 1.8.4.10] | AMGI |
| PSA_SG16 | IMGVR_UViG_3300005589_000008 | IMGVR_UViG_3300005589_000008\|_50 | K01520 | 7.30E-35 | dUTP pyrophosphatase [EC:3.6.1.23] | AMGI |
| PSA_SG16 | IMGVR_UViG_3300005589_000008 | IMGVR_UViG_3300005589_000008\|_65 | K19303 | 1.10E-11 | murein DD-endopeptidase [EC:3.4.-.-] | AMGII |
| PSA_SG16 | IMGVR_UViG_3300005609_000001 | IMGVR_UViG_3300005609_000001\|_16 | K01520 | 7.30E-35 | dUTP pyrophosphatase [EC:3.6.1.23] | AMGI |
| PSA_SG16 | IMGVR_UViG_3300005609_000001 | IMGVR_UViG_3300005609_000001\|_38 | K00390 | 2.7E-07 | phosphoadenosine phosphosulfate reductase [EC:1.8.4.8 1.8.4.10] | AMGI |
| PSA_SG16 | IMGVR_UViG_3300005609_000001 | IMGVR_UViG_3300005609_000001\|_1 | K19224 | 3.6E-07 | peptidoglycan DL-endopeptidase LytE [EC:3.4.-.-] | AMGII |
| PSA_MG4 | IMGVR_UViG_3300005658_000006 | IMGVR_UViG_3300005658_000006\|_9 | K08640 | 2.90E-10 | zinc D-Ala-D-Ala carboxypeptidase [EC:3.4.17.14] | AMGII |
| PSA_MG4 | IMGVR_UViG_3300005658_000006 | IMGVR_UViG_3300005658_000006\|_37 | K20993 | 7.8E-06 | pyrethroid hydrolase [EC:3.1.1.88] | AMGII |
| PSA_SG16 | IMGVR_UViG_3300005920_000002 | IMGVR_UViG_3300005920_000002\|_5 | K01520 | 7.30E-35 | dUTP pyrophosphatase [EC:3.6.1.23] | AMGI |
| PSA_SG16 | IMGVR_UViG_3300005920_000002 | IMGVR_UViG_3300005920_000002\|_27 | K00390 | 2.7E-07 | phosphoadenosine phosphosulfate reductase [EC:1.8.4.8 1.8.4.10] | AMGI |
| PSA_SG16 | IMGVR_UViG_3300005920_000002 | IMGVR_UViG_3300005920_000002\|_63 | K19303 | 1.10E-11 | murein DD-endopeptidase [EC:3.4.-.-] | AMGII |
| PSA_SG16 | IMGVR_UViG_3300006467_000168 | IMGVR_UViG_3300006467_000168\|_21 | K01520 | 7.30E-35 | dUTP pyrophosphatase [EC:3.6.1.23] | AMGI |
| PSA_SG16 | IMGVR_UViG_3300006467_000168 | IMGVR_UViG_3300006467_000168\|_43 | K00390 | 2.7E-07 | phosphoadenosine phosphosulfate reductase [EC:1.8.4.8 1.8.4.10] | AMGI |
| PSA_SG16 | IMGVR_UViG_3300006467_000168 | IMGVR_UViG_3300006467_000168\|_6 | K19303 | 1.10E-11 | murein DD-endopeptidase [EC:3.4.-.-] | AMGII |
| PSA_PG1 | IMGVR_UViG_3300009428_000011 | IMGVR_UViG_3300009428_000011\|_56 | K04962 | 1.90E-11 | ryanodine receptor 2 | AMGII |
| PSA_MG3 | IMGVR_UViG_3300010385_000004 | IMGVR_UViG_3300010385_000004\|_28 | K10804 | 8.5E-08 | acyl-CoA thioesterase I [EC:3.1.2.- 3.1.2.2 3.1.1.2 3.1.1.5] | AMGI |
| PSA_SG16 | IMGVR_UViG_3300010392_000285 | IMGVR_UViG_3300010392_000285\|_5 | K01520 | 7.30E-35 | dUTP pyrophosphatase [EC:3.6.1.23] | AMGI |
| PSA_SG16 | IMGVR_UViG_3300010392_000285 | IMGVR_UViG_3300010392_000285\|_27 | K00390 | 2.7E-07 | phosphoadenosine phosphosulfate reductase [EC:1.8.4.8 1.8.4.10] | AMGI |
| PSA_SG16 | IMGVR_UViG_3300010392_000285 | IMGVR_UViG_3300010392_000285\|_63 | K19303 | 1.10E-11 | murein DD-endopeptidase [EC:3.4.-.-] | AMGII |
| PSA_MG3 | IMGVR_UViG_3300010409_000001 | IMGVR_UViG_3300010409_000001\|_48 | K10804 | 8.5E-08 | acyl-CoA thioesterase I [EC:3.1.2.- 3.1.2.2 3.1.1.2 3.1.1.5] | AMGI |
| PSA_SG16 | IMGVR_UViG_3300010430_000118 | IMGVR_UViG_3300010430_000118\|_15 | K01520 | 7.30E-35 | dUTP pyrophosphatase [EC:3.6.1.23] | AMGI |
| PSA_SG16 | IMGVR_UViG_3300010430_000118 | IMGVR_UViG_3300010430_000118\|_37 | K00390 | 2.7E-07 | phosphoadenosine phosphosulfate reductase [EC:1.8.4.8 1.8.4.10] | AMGI |
| PSA_SG16 | IMGVR_UViG_3300010430_000118 | IMGVR_UViG_3300010430_000118\|_73 | K19303 | 1.10E-11 | murein DD-endopeptidase [EC:3.4.-.-] | AMGII |
| PSA_MG1 | IMGVR_UViG_3300010883_000156 | IMGVR_UViG_3300010883_000156\|_39 | K07816 | 9.00E-38 | putative GTP pyrophosphokinase [EC:2.7.6.5] | AMGI |
| PSA_MG1 | IMGVR_UViG_3300010883_000156 | IMGVR_UViG_3300010883_000156\|_6 | K13444 | 1.90E-12 | formylglycine-generating enzyme [EC:1.8.3.7] | AMGII |
| PSA_MG1 | IMGVR_UViG_3300010883_000156 | IMGVR_UViG_3300010883_000156\|_43 | K19155 | 1.10E-47 | toxin YhaV [EC:3.1.-.-] | AMGII |
| PSA_MG8 | IMGVR_UViG_3300019762_000002 | IMGVR_UViG_3300019762_000002\|_63 | K00390 | 3.2E-06 | phosphoadenosine phosphosulfate reductase [EC:1.8.4.8 1.8.4.10] | AMGI |
| PSA_SG7 | IMGVR_UViG_3300020369_000005 | IMGVR_UViG_3300020369_000005\|_1 | K08309 | 7.40E-16 | soluble lytic murein transglycosylase [EC:4.2.2.-] | AMGII |
| PSA_SG7 | IMGVR_UViG_3300020369_000005 | IMGVR_UViG_3300020369_000005\|_17 | K07782 | 7.9E-07 | LuxR family transcriptional regulator, quorum-sensing system regulator SdiA | AMGII |
| PSA_SG7 | IMGVR_UViG_3300020369_000005 | IMGVR_UViG_3300020369_000005\|_19 | K22719 | 9.2E-06 | murein hydrolase activator | AMGII |
| PSA_SG7 | IMGVR_UViG_3300020369_000005 | IMGVR_UViG_3300020369_000005\|_50 | K19479 | 1.60E-09 | collagen type X alpha | AMGII |
| PSA_SG7 | IMGVR_UViG_3300020369_000005 | IMGVR_UViG_3300020369_000005\|_67 | K07451 | 4.5E-06 | 5-methylcytosine-specific restriction enzyme A [EC:3.1.21.-] | AMGII |
| PSA_PG1 | IMGVR_UViG_3300020385_000019 | IMGVR_UViG_3300020385_000019\|_20 | K13444 | 5.40E-11 | formylglycine-generating enzyme [EC:1.8.3.7] | AMGII |
| PSA_PG1 | IMGVR_UViG_3300020385_000019 | IMGVR_UViG_3300020385_000019\|_28 | K04962 | 4.6E-08 | ryanodine receptor 2 | AMGII |
| Catalunyavirus | IMGVR_UViG_3300020423_000003 | IMGVR_UViG_3300020423_000003\|_13 | K22918 | 2.60E-38 | typhoid toxin secretion A | AMGII |
| PSA_MG4 | IMGVR_UViG_3300020423_000004 | IMGVR_UViG_3300020423_000004\|_10 | K08640 | 8.2E-07 | zinc D-Ala-D-Ala carboxypeptidase [EC:3.4.17.14] | AMGII |
| PSA_SG7 | IMGVR_UViG_3300020473_000002 | IMGVR_UViG_3300020473_000002\|_11 | K18691 | 7.10E-23 | membrane-bound lytic murein transglycosylase F [EC:4.2.2.-] | AMGII |
| PSA_SG7 | IMGVR_UViG_3300020473_000002 | IMGVR_UViG_3300020473_000002\|_27 | K07782 | 2.7E-08 | LuxR family transcriptional regulator, quorum-sensing system regulator SdiA | AMGII |
| PSA_SG7 | IMGVR_UViG_3300020473_000002 | IMGVR_UViG_3300020473_000002\|_54 | K06186 | 4.7E-06 | outer membrane protein assembly factor BamE | AMGII |
| PSA_MG4 | IMGVR_UViG_3300025122_000031 | IMGVR_UViG_3300025122_000031\|_41 | K08640 | 1.8E-08 | zinc D-Ala-D-Ala carboxypeptidase [EC:3.4.17.14] | AMGII |
| PSA_MG4 | IMGVR_UViG_3300025125_000003 | IMGVR_UViG_3300025125_000003\|_41 | K08640 | 1.8E-08 | zinc D-Ala-D-Ala carboxypeptidase [EC:3.4.17.14] | AMGII |
| PSA_MG4 | IMGVR_UViG_3300025132_000076 | IMGVR_UViG_3300025132_000076\|_40 | K08640 | 1.8E-08 | zinc D-Ala-D-Ala carboxypeptidase [EC:3.4.17.14] | AMGII |
| PSA_PG1 | IMGVR_UViG_3300025191_000002 | IMGVR_UViG_3300025191_000002\|_6 | K10804 | 6.20E-23 | acyl-CoA thioesterase I [EC:3.1.2.- 3.1.2.2 3.1.1.2 3.1.1.5] | AMGI |
| PSA_PG1 | IMGVR_UViG_3300025191_000002 | IMGVR_UViG_3300025191_000002\|_4 | K04962 | 8.70E-12 | ryanodine receptor 2 | AMGII |
| PSA_MG1 | IMGVR_UViG_3300026207_000002 | IMGVR_UViG_3300026207_000002\|_1 | K19155 | 2.90E-47 | toxin YhaV [EC:3.1.-.-] | AMGII |
| PSA_MG1 | IMGVR_UViG_3300026207_000002 | IMGVR_UViG_3300026207_000002\|_38 | K13444 | 9.30E-12 | formylglycine-generating enzyme [EC:1.8.3.7] | AMGII |
| Catalunyavirus | IMGVR_UViG_3300027372_000005 | IMGVR_UViG_3300027372_000005\|_42 | K01126 | 1.60E-43 | glycerophosphoryl diester phosphodiesterase [EC:3.1.4.46] | AMGI |
| PSA_SG16 | IMGVR_UViG_3300027758_000002 | IMGVR_UViG_3300027758_000002\|_9 | K00390 | 2.7E-07 | phosphoadenosine phosphosulfate reductase [EC:1.8.4.8 1.8.4.10] | AMGI |
| PSA_SG16 | IMGVR_UViG_3300027758_000002 | IMGVR_UViG_3300027758_000002\|_31 | K01520 | 7.30E-35 | dUTP pyrophosphatase [EC:3.6.1.23] | AMGI |
| PSA_SG16 | IMGVR_UViG_3300027758_000002 | IMGVR_UViG_3300027758_000002\|_46 | K19303 | 1.10E-11 | murein DD-endopeptidase [EC:3.4.-.-] | AMGII |
| PSA_SG16 | IMGVR_UViG_3300027828_000047 | IMGVR_UViG_3300027828_000047\|_50 | K00390 | 2.7E-07 | phosphoadenosine phosphosulfate reductase [EC:1.8.4.8 1.8.4.10] | AMGI |
| PSA_SG16 | IMGVR_UViG_3300027828_000047 | IMGVR_UViG_3300027828_000047\|_72 | K01520 | 7.30E-35 | dUTP pyrophosphatase [EC:3.6.1.23] | AMGI |
| PSA_SG16 | IMGVR_UViG_3300027828_000047 | IMGVR_UViG_3300027828_000047\|_14 | K19303 | 1.10E-11 | murein DD-endopeptidase [EC:3.4.-.-] | AMGII |
| PSA_AG1 | IMGVR_UViG_3300027861_000083 | IMGVR_UViG_3300027861_000083\|_11 | K16226 | 3.9E-07 | disease resistance protein RPS4 | AMGII |
| PSA_SG16 | IMGVR_UViG_3300027967_000003 | IMGVR_UViG_3300027967_000003\|_43 | K01520 | 7.30E-35 | dUTP pyrophosphatase [EC:3.6.1.23] | AMGI |
| PSA_SG16 | IMGVR_UViG_3300027967_000003 | IMGVR_UViG_3300027967_000003\|_65 | K00390 | 2.7E-07 | phosphoadenosine phosphosulfate reductase [EC:1.8.4.8 1.8.4.10] | AMGI |
| PSA_SG16 | IMGVR_UViG_3300027967_000003 | IMGVR_UViG_3300027967_000003\|_28 | K19303 | 1.10E-11 | murein DD-endopeptidase [EC:3.4.-.-] | AMGII |
| PSA_SG16 | IMGVR_UViG_3300027980_000047 | IMGVR_UViG_3300027980_000047\|_9 | K00390 | 2.7E-07 | phosphoadenosine phosphosulfate reductase [EC:1.8.4.8 1.8.4.10] | AMGI |
| PSA_SG16 | IMGVR_UViG_3300027980_000047 | IMGVR_UViG_3300027980_000047\|_31 | K01520 | 7.30E-35 | dUTP pyrophosphatase [EC:3.6.1.23] | AMGI |
| PSA_SG16 | IMGVR_UViG_3300027980_000047 | IMGVR_UViG_3300027980_000047\|_46 | K19303 | 1.10E-11 | murein DD-endopeptidase [EC:3.4.-.-] | AMGII |
| PSA_SG7 | IMGVR_UViG_3300031660_000008 | IMGVR_UViG_3300031660_000008\|_9 | K08309 | 4.40E-25 | soluble lytic murein transglycosylase [EC:4.2.2.-] | AMGII |
| PSA_SG7 | IMGVR_UViG_3300031660_000008 | IMGVR_UViG_3300031660_000008\|_15 | K07451 | 4.7E-07 | 5-methylcytosine-specific restriction enzyme A [EC:3.1.21.-] | AMGII |
| PSA_SG7 | IMGVR_UViG_3300031660_000008 | IMGVR_UViG_3300031660_000008\|_39 | K06186 | 7.2E-06 | outer membrane protein assembly factor BamE | AMGII |
| PSA_SG7 | IMGVR_UViG_3300031660_000008 | IMGVR_UViG_3300031660_000008\|_63 | K10421 | 9.2E-06 | CAP-Gly domain-containing linker protein 1 | AMGII |
| PSA_SG7 | IMGVR_UViG_3300031660_000008 | IMGVR_UViG_3300031660_000008\|_65 | K07782 | 3.4E-08 | LuxR family transcriptional regulator, quorum-sensing system regulator SdiA | AMGII |
| PSA_SG7 | IMGVR_UViG_3300031696_000009 | IMGVR_UViG_3300031696_000009\|_1 | K07451 | 4.7E-07 | 5-methylcytosine-specific restriction enzyme A [EC:3.1.21.-] | AMGII |
| PSA_SG7 | IMGVR_UViG_3300031696_000009 | IMGVR_UViG_3300031696_000009\|_7 | K08309 | 4.40E-25 | soluble lytic murein transglycosylase [EC:4.2.2.-] | AMGII |
| PSA_SG7 | IMGVR_UViG_3300031696_000009 | IMGVR_UViG_3300031696_000009\|_22 | K07782 | 3.4E-08 | LuxR family transcriptional regulator, quorum-sensing system regulator SdiA | AMGII |
| PSA_SG7 | IMGVR_UViG_3300031696_000009 | IMGVR_UViG_3300031696_000009\|_24 | K10421 | 9.2E-06 | CAP-Gly domain-containing linker protein 1 | AMGII |
| PSA_SG7 | IMGVR_UViG_3300031696_000009 | IMGVR_UViG_3300031696_000009\|_48 | K06186 | 7.2E-06 | outer membrane protein assembly factor BamE | AMGII |
| PSA_MG1 | IMGVR_UViG_3300032273_000003 | IMGVR_UViG_3300032273_000003\|_5 | K07816 | 6.50E-43 | putative GTP pyrophosphokinase [EC:2.7.6.5] | AMGI |
| PSA_MG1 | IMGVR_UViG_3300032273_000003 | IMGVR_UViG_3300032273_000003\|_2 | K19155 | 1.10E-46 | toxin YhaV [EC:3.1.-.-] | AMGII |
| PSA_SG6 | IMGVR_UViG_3300032360_000014 | IMGVR_UViG_3300032360_000014\|_7 | K00390 | 1.2E-07 | phosphoadenosine phosphosulfate reductase [EC:1.8.4.8 1.8.4.10] | AMGI |
| PSA_SG6 | IMGVR_UViG_3300032360_000014 | IMGVR_UViG_3300032360_000014\|_16 | K01419 | 5.5E-06 | ATP-dependent HslUV protease, peptidase subunit HslV [EC:3.4.25.2] | AMGII |
| PSA_SG6 | IMGVR_UViG_3300032360_000014 | IMGVR_UViG_3300032360_000014\|_28 | K11909 | 5.00E-20 | type VI secretion system protein VasI | AMGII |
| PSA_SG6 | IMGVR_UViG_3300032360_000014 | IMGVR_UViG_3300032360_000014\|_47 | K07451 | 0.000009 | 5-methylcytosine-specific restriction enzyme A [EC:3.1.21.-] | AMGII |
| PSA_SG6 | IMGVR_UViG_3300032360_000014 | IMGVR_UViG_3300032360_000014\|_64 | K10421 | 6.3E-08 | CAP-Gly domain-containing linker protein 1 | AMGII |
| PSA_SG6 | IMGVR_UViG_3300032360_000014 | IMGVR_UViG_3300032360_000014\|_65 | K05636 | 6.1E-06 | laminin, beta 1 | AMGII |
| PSA_SG3 | Kang_2014-00036_D_NODE_6 | Kang_2014-00036_D_NODE_6\|_18 | K10804 | 1.70E-11 | acyl-CoA thioesterase I [EC:3.1.2.- 3.1.2.2 3.1.1.2 3.1.1.5] | AMGI |
| PSA_SG13 | KC542353 | KC542353\|_17 | K13694 | 6.3E-06 | murein DD-endopeptidase / murein LD-carboxypeptidase [EC:3.4.-.- 3.4.17.13] | AMGII |
| PSA_SG9 | KF302032 | KF302032\|_5 | K08640 | 1.40E-18 | zinc D-Ala-D-Ala carboxypeptidase [EC:3.4.17.14] | AMGII |
| PSA_SG9 | KF302033 | KF302033\|_53 | K08640 | 1.40E-18 | zinc D-Ala-D-Ala carboxypeptidase [EC:3.4.17.14] | AMGII |
| PSA_MG7 | KF302034 | KF302034\|_184 | K03465 | 2.20E-15 | thymidylate synthase (FAD) [EC:2.1.1.148] | AMGI |
| PSA_MG7 | KF302034 | KF302034\|_187 | K00525 | 4.70E-32 | ribonucleoside-diphosphate reductase alpha chain [EC:1.17.4.1] | AMGI |
| PSA_MG7 | KF302034 | KF302034\|_189 | K00525 | 8.30E-40 | ribonucleoside-diphosphate reductase alpha chain [EC:1.17.4.1] | AMGI |
| PSA_MG7 | KF302034 | KF302034\|_228 | K17462 | 5.2E-06 | putative AdoMet-dependent methyltransferase [EC:2.1.1.-] | AMGI |
| PSA_MG7 | KF302034 | KF302034\|_17 | K15529 | 1.2E-06 | ADP-ribose 1''-phosphate phosphatase [EC:3.1.3.84] | AMGII |
| PSA_MG7 | KF302034 | KF302034\|_94 | K09952 | 3.70E-11 | CRISPR-associated endonuclease Csn1 [EC:3.1.-.-] | AMGII |
| PSA_MG7 | KF302034 | KF302034\|_181 | K07451 | 1.1E-06 | 5-methylcytosine-specific restriction enzyme A [EC:3.1.21.-] | AMGII |
| PSA_MG7 | KF302034 | KF302034\|_194 | K22918 | 3.40E-45 | typhoid toxin secretion A | AMGII |
| PSA_MG7 | KF302034 | KF302034\|_197 | K06217 | 1.90E-25 | phosphate starvation-inducible protein PhoH and related proteins | AMGII |
| PSA_MG7 | KF302034 | KF302034\|_199 | K01358 | 1.6E-08 | ATP-dependent Clp protease, protease subunit [EC:3.4.21.92] | AMGII |
| PSA_SG3 | KF302035 | KF302035\|_19 | K10804 | 1.70E-11 | acyl-CoA thioesterase I [EC:3.1.2.- 3.1.2.2 3.1.1.2 3.1.1.5] | AMGI |
| PSA_SG10 | KF302036 | KF302036\|_27 | K01520 | 5.30E-34 | dUTP pyrophosphatase [EC:3.6.1.23] | AMGI |
| PSA_SG10 | KF302036 | KF302036\|_64 | K22228 | 6.6E-06 | preprotein translocase subunit SecG | AMGII |
| PSA_SG15 | KU747973 | KU747973\|_2 | K13695 | 1.20E-10 | probable lipoprotein NlpC | AMGII |
| PSA_SG15 | KU747973 | KU747973\|_10 | K17733 | 2.70E-24 | peptidoglycan LD-endopeptidase CwlK [EC:3.4.-.-] | AMGII |
| PSA_SG15 | KU747973 | KU747973\|_29 | K07464 | 1.4E-06 | CRISPR-associated exonuclease Cas4 [EC:3.1.12.1] | AMGII |
| PSA_PG2 | KX257490 | KX257490\|_22 | K17733 | 6.20E-25 | peptidoglycan LD-endopeptidase CwlK [EC:3.4.-.-] | AMGII |
| PSA_MG5 | KX822733 | KX822733\|_72 | K00526 | 1.70E-82 | ribonucleoside-diphosphate reductase beta chain [EC:1.17.4.1] | AMGI |
| PSA_MG5 | KX822733 | KX822733\|_76 | K00287 | 2.10E-35 | dihydrofolate reductase [EC:1.5.1.3] | AMGI |
| PSA_MG5 | KX822733 | KX822733\|_78 | K00560 | 2.60E-32 | thymidylate synthase [EC:2.1.1.45] | AMGI |
| PSA_MG5 | KX822733 | KX822733\|_200 | K03462 | 4.10E-80 | nicotinamide phosphoribosyltransferase [EC:2.4.2.12] | AMGI |
| PSA_MG5 | KX822733 | KX822733\|_201 | K00948 | 1.90E-20 | ribose-phosphate pyrophosphokinase [EC:2.7.6.1] | AMGI |
| PSA_MG5 | KX822733 | KX822733\|_66 | K01358 | 7.10E-09 | ATP-dependent Clp protease, protease subunit [EC:3.4.21.92] | AMGII |
| PSA_MG5 | KX822733 | KX822733\|_67 | K07175 | 6.00E-27 | PhoH-like ATPase | AMGII |
| PSA_MG5 | KX822733 | KX822733\|_83 | K07451 | 1.9E-06 | 5-methylcytosine-specific restriction enzyme A [EC:3.1.21.-] | AMGII |
| PSA_MG5 | KX822733 | KX822733\|_202 | K15529 | 7E-07 | ADP-ribose 1''-phosphate phosphatase [EC:3.1.3.84] | AMGII |
| PSA_SG8 | KX912252 | KX912252\|_56 | K01520 | 2.00E-34 | dUTP pyrophosphatase [EC:3.6.1.23] | AMGI |
| PSA_SG5 | MF098558 | MF098558\|_54 | K00390 | 4.40E-11 | phosphoadenosine phosphosulfate reductase [EC:1.8.4.8 1.8.4.10] | AMGI |
| PSA_SG5 | MF098558 | MF098558\|_1 | K13444 | 2.80E-11 | formylglycine-generating enzyme [EC:1.8.3.7] | AMGII |
| PSA_SG5 | MF098558 | MF098558\|_50 | K03427 | 5.1E-07 | type I restriction enzyme M protein [EC:2.1.1.72] | AMGII |
| PSA_SG5 | MF098558 | MF098558\|_75 | K03607 | 1.90E-19 | ProP effector | AMGII |
| Qingdaovirus | MF988720 | MF988720\|_48 | K10837 | 4.7E-07 | O-phosphoseryl-tRNA(Sec) kinase [EC:2.7.1.164] | AMGI |
| Qingdaovirus | MF988720 | MF988720\|_54 | K03462 | 3.40E-111 | nicotinamide phosphoribosyltransferase [EC:2.4.2.12] | AMGI |
| Qingdaovirus | MF988720 | MF988720\|_139 | K00526 | 6.30E-81 | ribonucleoside-diphosphate reductase beta chain [EC:1.17.4.1] | AMGI |
| Qingdaovirus | MF988720 | MF988720\|_143 | K00287 | 1.10E-36 | dihydrofolate reductase [EC:1.5.1.3] | AMGI |
| Qingdaovirus | MF988720 | MF988720\|_144 | K00560 | 4.10E-45 | thymidylate synthase [EC:2.1.1.45] | AMGI |
| Qingdaovirus | MF988720 | MF988720\|_120 | K09935 | 3.50E-12 | N-glycosidase YbiA [EC:3.2.2.-] | AMGII |
| Qingdaovirus | MF988720 | MF988720\|_136 | K22918 | 9.60E-40 | typhoid toxin secretion A | AMGII |
| Qingdaovirus | MF988720 | MF988720\|_158 | K13753 | 3.20E-45 | solute carrier family 24 (sodium/potassium/calcium exchanger), member 5 | AMGII |
| Qingdaovirus | MF988720 | MF988720\|_163 | K05795 | 8.30E-22 | tellurium resistance protein TerD | AMGII |
| Qingdaovirus | MF988720 | MF988720\|_164 | K05795 | 3.30E-15 | tellurium resistance protein TerD | AMGII |
| PSA_MG2 | MG675557 | MG675557\|_47 | K10804 | 3.70E-15 | acyl-CoA thioesterase I [EC:3.1.2.- 3.1.2.2 3.1.1.2 3.1.1.5] | AMGI |
| PSA_SG12 | MK387309 | MK387309\|_59 | K01520 | 9.10E-34 | dUTP pyrophosphatase [EC:3.6.1.23] | AMGI |
| PSA_SG12 | MK387309 | MK387309\|_3 | K19303 | 3.1E-06 | murein DD-endopeptidase [EC:3.4.-.-] | AMGII |
| PSA_SG12 | MK387309 | MK387309\|_53 | K07464 | 1.5E-06 | CRISPR-associated exonuclease Cas4 [EC:3.1.12.1] | AMGII |
| Corticovirus | MK424903 | MK424903\|_16 | K12063 | 6.7E-07 | conjugal transfer ATP-binding protein TraC | AMGII |
| PSA_SG14 | MN313256 | MN313256\|_41 | K00390 | 3.40E-09 | phosphoadenosine phosphosulfate reductase [EC:1.8.4.8 1.8.4.10] | AMGI |
| PSA_SG14 | MN313256 | MN313256\|_42 | K06720 | 1.50E-34 | L-ectoine synthase [EC:4.2.1.108] | AMGI |
| PSA_SG14 | MN313256 | MN313256\|_58 | K19224 | 1.5E-07 | peptidoglycan DL-endopeptidase LytE [EC:3.4.-.-] | AMGII |
| PSA_SG15 | MT002874 | MT002874\|_53 | K00390 | 7.70E-26 | phosphoadenosine phosphosulfate reductase [EC:1.8.4.8 1.8.4.10] | AMGI |
| PSA_SG15 | MT002874 | MT002874\|_33 | K05636 | 8.50E-10 | laminin, beta 1 | AMGII |
| PSA_SG15 | MT002874 | MT002874\|_40 | K17733 | 3.00E-23 | peptidoglycan LD-endopeptidase CwlK [EC:3.4.-.-] | AMGII |
| PSA_SG9 | NC_015293 | NC_015293\|_49 | K08640 | 1.40E-18 | zinc D-Ala-D-Ala carboxypeptidase [EC:3.4.17.14] | AMGII |
| PSA_PG4 | NC_020849 | NC_020849\|_64 | K03465 | 4.20E-54 | thymidylate synthase (FAD) [EC:2.1.1.148] | AMGI |
| PSA_PG4 | NC_020849 | NC_020849\|_68 | K00525 | 3.10E-45 | ribonucleoside-diphosphate reductase alpha chain [EC:1.17.4.1] | AMGI |
| PSA_PG4 | NC_020849 | NC_020849\|_71 | K00526 | 9.50E-52 | ribonucleoside-diphosphate reductase beta chain [EC:1.17.4.1] | AMGI |
| PSA_PG4 | NC_020849 | NC_020849\|_93 | K08640 | 1.20E-18 | zinc D-Ala-D-Ala carboxypeptidase [EC:3.4.17.14] | AMGII |
| PSA_PG4 | NC_020849 | NC_020849\|_96 | K19721 | 0.00001 | collagen type V/XI/XXIV/XXVII, alpha | AMGII |
| PSA_PG4 | NC_020849 | NC_020849\|_104 | K01358 | 2.60E-14 | ATP-dependent Clp protease, protease subunit [EC:3.4.21.92] | AMGII |
| Melvirus | NC_021300 | NC_021300\|_7 | K13564 | 9.7E-06 | gamma-L-glutamyl-butirosin B gamma-L-glutamyl cyclotransferase [EC:4.3.2.6] | AMGI |
| Melvirus | NC_021300 | NC_021300\|_8 | K00820 | 3.20E-17 | glutamine---fructose-6-phosphate transaminase (isomerizing) [EC:2.6.1.16] | AMGI |
| PSA_SG14 | NC_028819 | NC_028819\|_12 | K21471 | 7.4E-08 | peptidoglycan DL-endopeptidase CwlO [EC:3.4.-.-] | AMGII |
| PSA_MG6 | NC_029094 | NC_029094\|_22 | K10804 | 2.40E-10 | acyl-CoA thioesterase I [EC:3.1.2.- 3.1.2.2 3.1.1.2 3.1.1.5] | AMGI |
| PSA_MG6 | NC_029094 | NC_029094\|_74 | K00948 | 5.20E-26 | ribose-phosphate pyrophosphokinase [EC:2.7.6.1] | AMGI |
| PSA_MG6 | NC_029094 | NC_029094\|_76 | K03462 | 3.60E-115 | nicotinamide phosphoribosyltransferase [EC:2.4.2.12] | AMGI |
| PSA_MG6 | NC_029094 | NC_029094\|_121 | K01525 | 4.60E-22 | bis(5'-nucleosyl)-tetraphosphatase (symmetrical) [EC:3.6.1.41] | AMGI |
| PSA_MG6 | NC_029094 | NC_029094\|_140 | K00525 | 6.90E-116 | ribonucleoside-diphosphate reductase alpha chain [EC:1.17.4.1] | AMGI |
| PSA_MG6 | NC_029094 | NC_029094\|_143 | K00287 | 3.50E-38 | dihydrofolate reductase [EC:1.5.1.3] | AMGI |
| PSA_MG6 | NC_029094 | NC_029094\|_145 | K00560 | 2.70E-54 | thymidylate synthase [EC:2.1.1.45] | AMGI |
| PSA_MG6 | NC_029094 | NC_029094\|_122 | K01358 | 2.70E-11 | ATP-dependent Clp protease, protease subunit [EC:3.4.21.92] | AMGII |
| PSA_MG6 | NC_029094 | NC_029094\|_124 | K07175 | 3.90E-28 | PhoH-like ATPase | AMGII |
| PSA_MG6 | NC_029094 | NC_029094\|_128 | K22918 | 1.20E-35 | typhoid toxin secretion A | AMGII |
| PSA_SG8 | NC_029100 | NC_029100\|_50 | K01520 | 6.70E-33 | dUTP pyrophosphatase [EC:3.6.1.23] | AMGI |
| PSA_SG8 | NC_029100 | NC_029100\|_55 | K10804 | 5.60E-09 | acyl-CoA thioesterase I [EC:3.1.2.- 3.1.2.2 3.1.1.2 3.1.1.5] | AMGI |
| Kafunavirus | NC_031908 | NC_031908\|_10 | K00560 | 3.10E-59 | thymidylate synthase [EC:2.1.1.45] | AMGI |
| PSA_SG10 | NC_031917 | NC_031917\|_10 | K10804 | 2.60E-12 | acyl-CoA thioesterase I [EC:3.1.2.- 3.1.2.2 3.1.1.2 3.1.1.5] | AMGI |
| PSA_SG10 | NC_031917 | NC_031917\|_60 | K01520 | 4.30E-33 | dUTP pyrophosphatase [EC:3.6.1.23] | AMGI |
| Catalunyavirus | NC_047790 | NC_047790\|_23 | K23989 | 4.4E-07 | mannosyl-glycoprotein endo-beta-N-acetylglucosaminidase [EC:3.2.1.96] | AMGI |
| PSA_MG7 | NC_047839 | NC_047839\|_59 | K00560 | 3.50E-63 | thymidylate synthase [EC:2.1.1.45] | AMGI |
| PSA_MG7 | NC_047839 | NC_047839\|_60 | K00287 | 1.10E-37 | dihydrofolate reductase [EC:1.5.1.3] | AMGI |
| PSA_MG7 | NC_047839 | NC_047839\|_63 | K00525 | 1.80E-31 | ribonucleoside-diphosphate reductase alpha chain [EC:1.17.4.1] | AMGI |
| PSA_MG7 | NC_047839 | NC_047839\|_65 | K00525 | 4.60E-41 | ribonucleoside-diphosphate reductase alpha chain [EC:1.17.4.1] | AMGI |
| PSA_MG7 | NC_047839 | NC_047839\|_56 | K07451 | 1E-08 | 5-methylcytosine-specific restriction enzyme A [EC:3.1.21.-] | AMGII |
| PSA_MG7 | NC_047839 | NC_047839\|_71 | K22918 | 1.40E-45 | typhoid toxin secretion A | AMGII |
| PSA_MG7 | NC_047839 | NC_047839\|_72 | K07175 | 4.70E-27 | PhoH-like ATPase | AMGII |
| PSA_MG7 | NC_047839 | NC_047839\|_74 | K01358 | 7.50E-11 | ATP-dependent Clp protease, protease subunit [EC:3.4.21.92] | AMGII |
| PSA_MG7 | NC_047839 | NC_047839\|_118 | K15529 | 0.000008 | ADP-ribose 1''-phosphate phosphatase [EC:3.1.3.84] | AMGII |
| Melvirus | NC_048630 | NC_048630\|_40 | K13564 | 5.9E-07 | gamma-L-glutamyl-butirosin B gamma-L-glutamyl cyclotransferase [EC:4.3.2.6] | AMGI |
| Melvirus | NC_048630 | NC_048630\|_42 | K00820 | 1.30E-16 | glutamine---fructose-6-phosphate transaminase (isomerizing) [EC:2.6.1.16] | AMGI |
| PSA_SG15 | Station137_DCM_ALL_assembly_NODE_68 | Station137_DCM_ALL_assembly_NODE_68\|_27 | K19303 | 5.60E-09 | murein DD-endopeptidase [EC:3.4.-.-] | AMGII |
| PSA_SG16 | Station137_DCM_ALL_assembly_NODE_84 | Station137_DCM_ALL_assembly_NODE_84\|_36 | K01520 | 1.70E-35 | dUTP pyrophosphatase [EC:3.6.1.23] | AMGI |
| PSA_SG16 | Station137_DCM_ALL_assembly_NODE_84 | Station137_DCM_ALL_assembly_NODE_84\|_51 | K19224 | 0.000002 | peptidoglycan DL-endopeptidase LytE [EC:3.4.-.-] | AMGII |
| PSA_SG16 | Station137_MES_COMBINED_FINAL_NODE_1181 | Station137_MES_COMBINED_FINAL_NODE_1181\|_63 | K01520 | 1.70E-35 | dUTP pyrophosphatase [EC:3.6.1.23] | AMGI |
| PSA_SG16 | Station137_MES_COMBINED_FINAL_NODE_1181 | Station137_MES_COMBINED_FINAL_NODE_1181\|_78 | K19224 | 0.000002 | peptidoglycan DL-endopeptidase LytE [EC:3.4.-.-] | AMGII |
| PSA_SG16 | Station137_MES_DO_NOT_POOL_NODE_1181 | Station137_MES_DO_NOT_POOL_NODE_1181\|_63 | K01520 | 1.70E-35 | dUTP pyrophosphatase [EC:3.6.1.23] | AMGI |
| PSA_SG16 | Station137_MES_DO_NOT_POOL_NODE_1181 | Station137_MES_DO_NOT_POOL_NODE_1181\|_78 | K19224 | 0.000002 | peptidoglycan DL-endopeptidase LytE [EC:3.4.-.-] | AMGII |
| PSA_SG16 | Station137_SUR_ALL_assembly_NODE_95 | Station137_SUR_ALL_assembly_NODE_95\|_63 | K01520 | 1.70E-35 | dUTP pyrophosphatase [EC:3.6.1.23] | AMGI |
| PSA_SG16 | Station137_SUR_ALL_assembly_NODE_95 | Station137_SUR_ALL_assembly_NODE_95\|_78 | K19224 | 0.000002 | peptidoglycan DL-endopeptidase LytE [EC:3.4.-.-] | AMGII |
| PSA_SG16 | Station138_SUR_ALL_assembly_NODE_88 | Station138_SUR_ALL_assembly_NODE_88\|_53 | K01520 | 1.70E-35 | dUTP pyrophosphatase [EC:3.6.1.23] | AMGI |
| PSA_SG16 | Station138_SUR_ALL_assembly_NODE_88 | Station138_SUR_ALL_assembly_NODE_88\|_68 | K19224 | 0.000002 | peptidoglycan DL-endopeptidase LytE [EC:3.4.-.-] | AMGII |
| PSA_SG16 | Station158_SUR_ALL_assembly_NODE_1716 | Station158_SUR_ALL_assembly_NODE_1716\|_35 | K01520 | 3.60E-33 | dUTP pyrophosphatase [EC:3.6.1.23] | AMGI |
| PSA_SG16 | Station158_SUR_ALL_assembly_NODE_1716 | Station158_SUR_ALL_assembly_NODE_1716\|_54 | K19303 | 2.8E-06 | murein DD-endopeptidase [EC:3.4.-.-] | AMGII |
| PSA_SG7 | Station173_DCM_ALL_assembly_NODE_1367 | Station173_DCM_ALL_assembly_NODE_1367\|_8 | K07451 | 2E-07 | 5-methylcytosine-specific restriction enzyme A [EC:3.1.21.-] | AMGII |
| PSA_SG7 | Station173_DCM_ALL_assembly_NODE_1367 | Station173_DCM_ALL_assembly_NODE_1367\|_13 | K08309 | 1.00E-23 | soluble lytic murein transglycosylase [EC:4.2.2.-] | AMGII |
| PSA_SG7 | Station173_DCM_ALL_assembly_NODE_1367 | Station173_DCM_ALL_assembly_NODE_1367\|_31 | K07782 | 3.6E-08 | LuxR family transcriptional regulator, quorum-sensing system regulator SdiA | AMGII |
| PSA_SG7 | Station173_DCM_ALL_assembly_NODE_1367 | Station173_DCM_ALL_assembly_NODE_1367\|_64 | K06186 | 0.000006 | outer membrane protein assembly factor BamE | AMGII |
| PSA_MG4 | Station188_DCM_ALL_assembly_NODE_1032 | Station188_DCM_ALL_assembly_NODE_1032\|_44 | K08640 | 9.90E-09 | zinc D-Ala-D-Ala carboxypeptidase [EC:3.4.17.14] | AMGII |
| Catalunyavirus | Station193_SUR_ALL_assembly_NODE_911 | Station193_SUR_ALL_assembly_NODE_911\|_34 | K22918 | 1.90E-36 | typhoid toxin secretion A | AMGII |
| PSA_SG4 | Station201_DCM_ALL_assembly_NODE_823 | Station201_DCM_ALL_assembly_NODE_823\|_30 | K10804 | 1.7E-08 | acyl-CoA thioesterase I [EC:3.1.2.- 3.1.2.2 3.1.1.2 3.1.1.5] | AMGI |
| PSA_SG4 | Station201_DCM_ALL_assembly_NODE_823 | Station201_DCM_ALL_assembly_NODE_823\|_9 | K13695 | 8.6E-07 | probable lipoprotein NlpC | AMGII |
| PSA_SG4 | Station201_DCM_ALL_assembly_NODE_823 | Station201_DCM_ALL_assembly_NODE_823\|_33 | K18691 | 2.50E-28 | membrane-bound lytic murein transglycosylase F [EC:4.2.2.-] | AMGII |
| PSA_PG1 | Station205_SUR_ALL_assembly_NODE_403 | Station205_SUR_ALL_assembly_NODE_403\|_47 | K04962 | 4.7E-08 | ryanodine receptor 2 | AMGII |
| PSA_SG1 | Station76_MES_COMBINED_FINAL_NODE_1001 | Station76_MES_COMBINED_FINAL_NODE_1001\|_53 | K01520 | 7.50E-22 | dUTP pyrophosphatase [EC:3.6.1.23] | AMGI |
| PSA_SG11 | Station78_MES_COMBINED_FINAL_NODE_1040 | Station78_MES_COMBINED_FINAL_NODE_1040\|_24 | K12212 | 2.2E-06 | intracellular multiplication protein IcmJ | AMGII |
| PSA_SG11 | Station78_MES_COMBINED_FINAL_NODE_1040 | Station78_MES_COMBINED_FINAL_NODE_1040\|_66 | K08640 | 1.40E-19 | zinc D-Ala-D-Ala carboxypeptidase [EC:3.4.17.14] | AMGII |
| PSA_SG9 | Station82_SUR_COMBINED_FINAL_NODE_658 | Station82_SUR_COMBINED_FINAL_NODE_658\|_8 | K07451 | 6.20E-11 | 5-methylcytosine-specific restriction enzyme A [EC:3.1.21.-] | AMGII |
| PSA_MG1 | Station82_SUR_COMBINED_FINAL_NODE_838 | Station82_SUR_COMBINED_FINAL_NODE_838\|_27 | K13444 | 3.20E-12 | formylglycine-generating enzyme [EC:1.8.3.7] | AMGII |
| Catalunyavirus | Station84_SUR_COMBINED_FINAL_NODE_1140 | Station84_SUR_COMBINED_FINAL_NODE_1140\|_38 | K10804 | 2.50E-19 | acyl-CoA thioesterase I [EC:3.1.2.- 3.1.2.2 3.1.1.2 3.1.1.5] | AMGI |
| Catalunyavirus | Station84_SUR_COMBINED_FINAL_NODE_1140 | Station84_SUR_COMBINED_FINAL_NODE_1140\|_30 | K22918 | 1.80E-36 | typhoid toxin secretion A | AMGII |
| PSA_SG16 | Station85_MES_COMBINED_FINAL_NODE_626 | Station85_MES_COMBINED_FINAL_NODE_626\|_22 | K01520 | 1.70E-35 | dUTP pyrophosphatase [EC:3.6.1.23] | AMGI |
| PSA_SG16 | Station85_MES_COMBINED_FINAL_NODE_626 | Station85_MES_COMBINED_FINAL_NODE_626\|_7 | K19224 | 0.000002 | peptidoglycan DL-endopeptidase LytE [EC:3.4.-.-] | AMGII |
| PSA_SG7 | Station85_SUR_COMBINED_FINAL_NODE_542 | Station85_SUR_COMBINED_FINAL_NODE_542\|_8 | K07451 | 2.6E-07 | 5-methylcytosine-specific restriction enzyme A [EC:3.1.21.-] | AMGII |
| PSA_SG7 | Station85_SUR_COMBINED_FINAL_NODE_542 | Station85_SUR_COMBINED_FINAL_NODE_542\|_11 | K08309 | 1.20E-23 | soluble lytic murein transglycosylase [EC:4.2.2.-] | AMGII |
| PSA_SG7 | Station85_SUR_COMBINED_FINAL_NODE_542 | Station85_SUR_COMBINED_FINAL_NODE_542\|_27 | K07782 | 3.6E-08 | LuxR family transcriptional regulator, quorum-sensing system regulator SdiA | AMGII |
| PSA_SG7 | Station85_SUR_COMBINED_FINAL_NODE_542 | Station85_SUR_COMBINED_FINAL_NODE_542\|_50 | K06186 | 7.3E-06 | outer membrane protein assembly factor BamE | AMGII |
| PSA_SG7 | Station85_SUR_COMBINED_FINAL_NODE_542 | Station85_SUR_COMBINED_FINAL_NODE_542\|_62 | K13444 | 5.50E-13 | formylglycine-generating enzyme [EC:1.8.3.7] | AMGII |
| Catalunyavirus | Station85_SUR_COMBINED_FINAL_NODE_902 | Station85_SUR_COMBINED_FINAL_NODE_902\|_4 | K22278 | 1.90E-10 | peptidoglycan-N-acetylglucosamine deacetylase [EC:3.5.1.104] | AMGII |


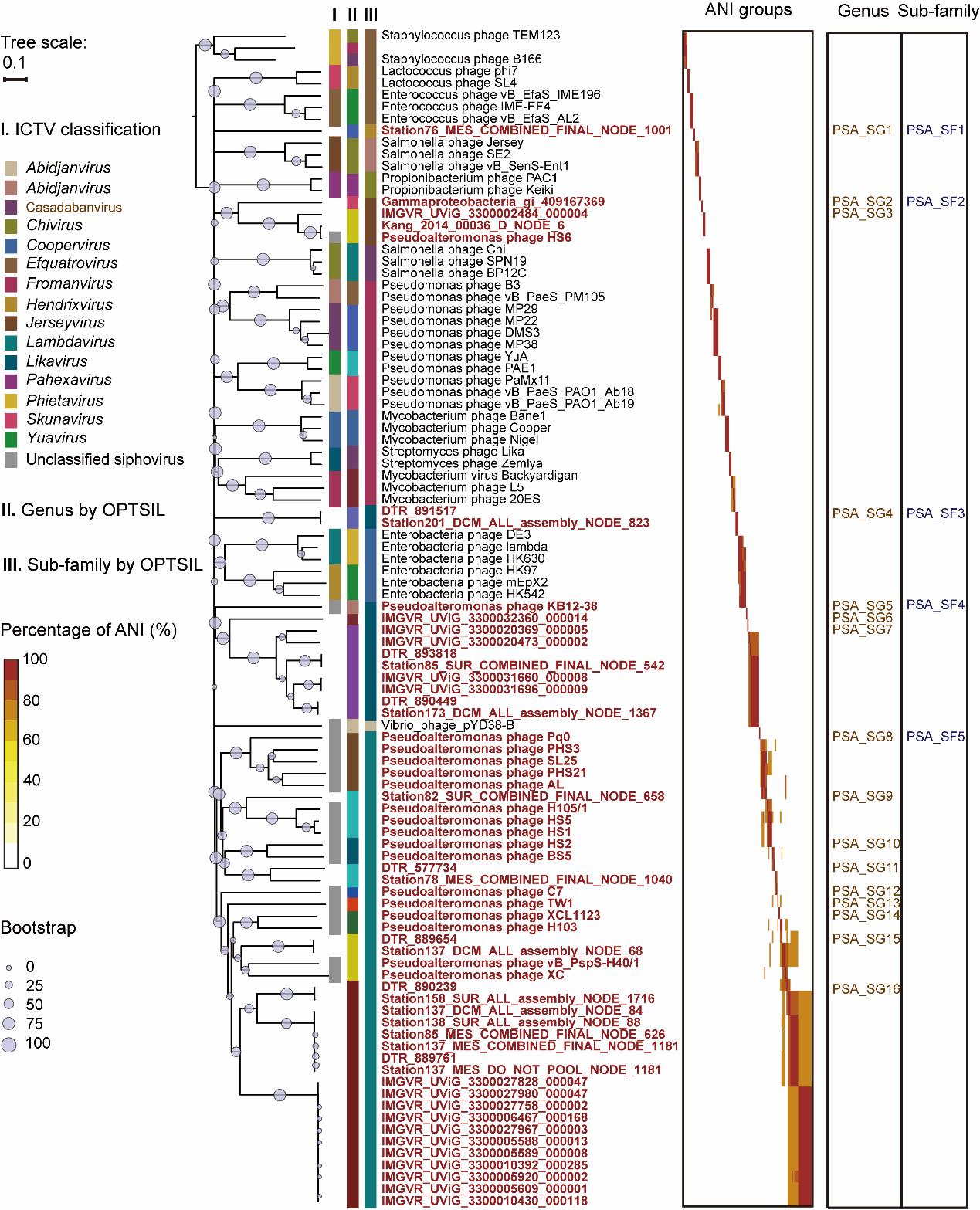


**Fig. S1.** Whole-genome based phylogenic trees and average nucleotide identity (ANI) clustering of 57 *Pseudoalteromonas* Siphoviral genomes with Siphoviral references*.* The 5 subfamily-leveled viral clusters belonging to *Siphoviridae* were assigned by OPTSIL, correcting and verifying by ANI clustering and VIRIDIC, including 16 genera and proposed genera. The bootstrap of each branch node was indicated by circle proportional to the value. Three series of color boxes behind the tree indicate: I. Genera classified by ICTV; II. Genus-leveled VCs of all these *Pseudoalteromonas* phages classified by OPTSIL; III. Subfamily-leveled viral clusters of all these *Pseudoalteromonas* phages classified by OPTSIL. Legends of tree scale, number of bootstraps, ICTV classification and ANI are indicated on the leftward of the tree.


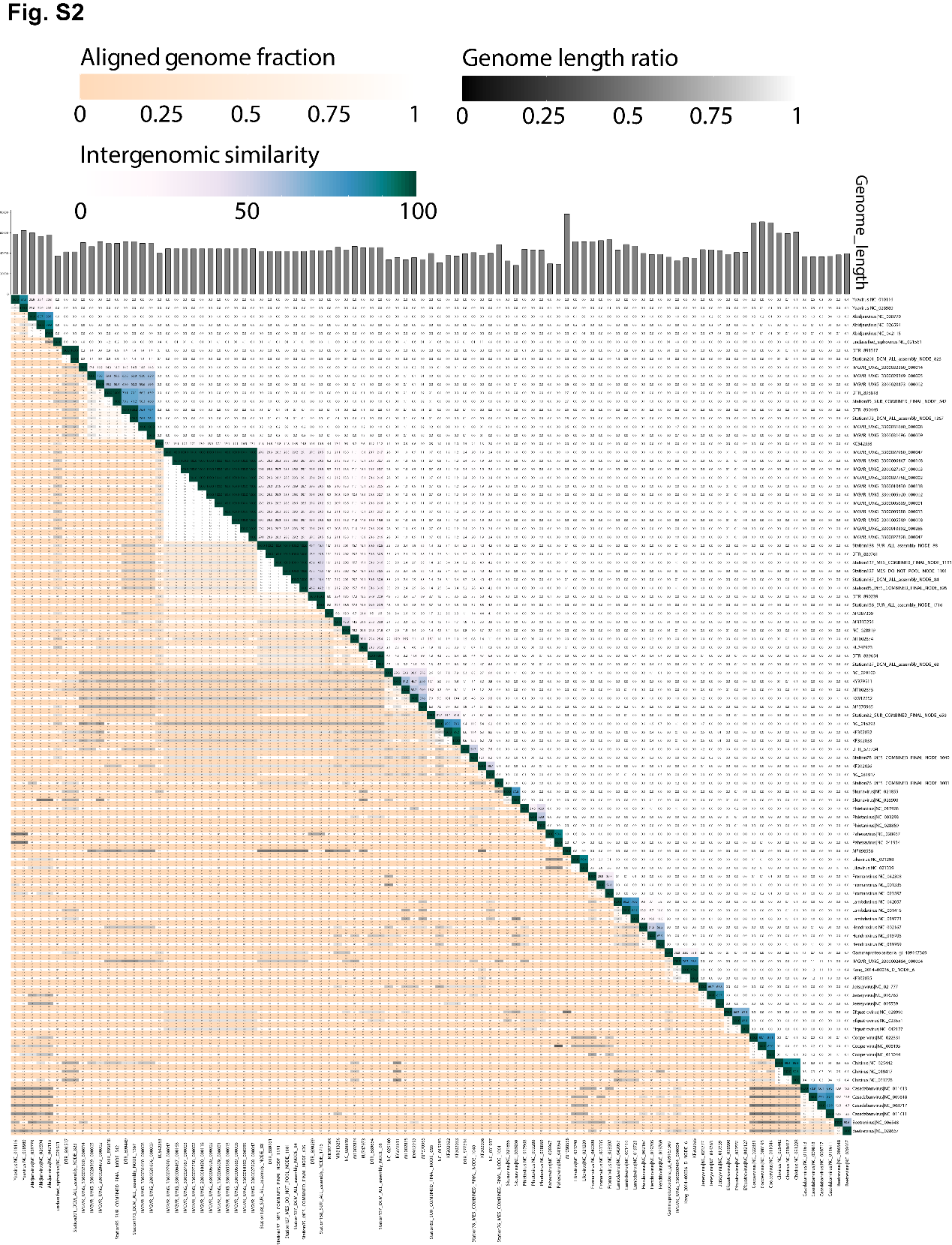


Fig. S2 The heatmaps generated by the VIRIDIC, showing the intergenomic relationships of *Siphoviridae*, *Myoviridae*, *Podoviridae/Zebellviridae/Schitoviridae*, *Autographiviridae* and *Inoviridae*, respectively. The intergenomic average nucleotides identity between transient pairs was shown in the right half of the heatmaps. The darker colors emphasize low values, indicating genome fractions are expected to decrease with increasing the distance between the phages. The boundary in the right half of the heatmaps indicates different genus-leveled VC.


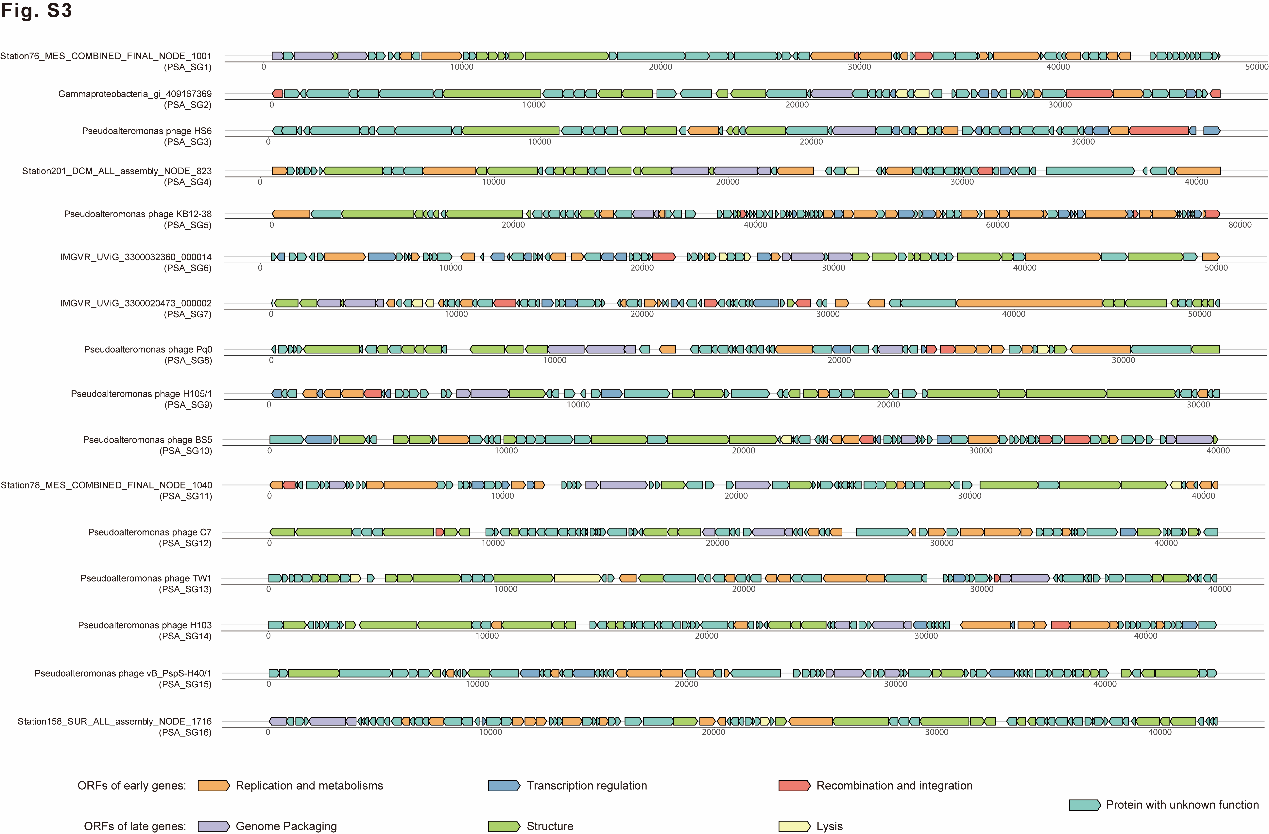


Fig. S3 The gene maps of representative Pseudoalteromonas-associated phage genomes (PSAPGs) in *Siphoviridae*, *Myoviridae*, *Podoviridae/Zebellviridae/Schitoviridae*, *Autographiviridae* and *Inoviridae*, respectively. The six functional modules were classified and indicated by different colors. The potential metabolism auxiliary genes (AMGs) were classified into the ‘Replication and metabolisms’ modules here.


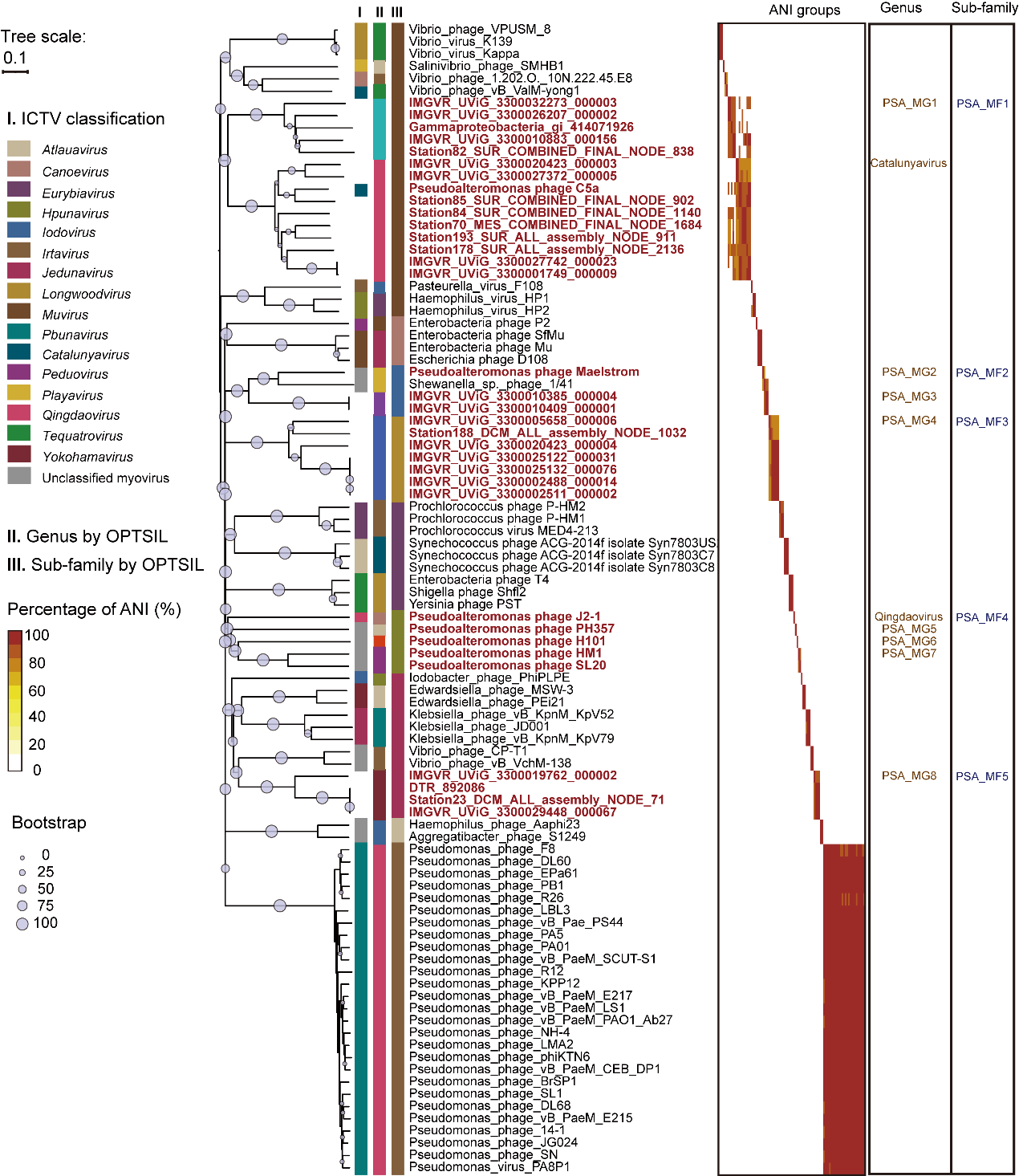


**Fig. S4.** Whole-genome based phylogenic trees and average nucleotide identity (ANI) clustering of 34 *Pseudoalteromonas* Myoviral genomes with Myoviral references*.* The 5 subfamily-leveled viral clusters belonging to *Myoviridae* were assigned by OPTSIL, correcting and verifying by ANI clustering and VIRIDIC, including 10 genera and proposed genera. The bootstrap of each branch node was indicated by circle proportional to the value. Three series of color boxes behind the tree indicate: I. Genera classified by ICTV; II. Genus-leveled VCs of all these *Pseudoalteromonas* phages classified by OPTSIL; III. Subfamily-leveled viral clusters of all these *Pseudoalteromonas* phages classified by OPTSIL. Legends of tree scale, number of bootstraps, ICTV classification and ANI are indicated on the leftward of the tree.


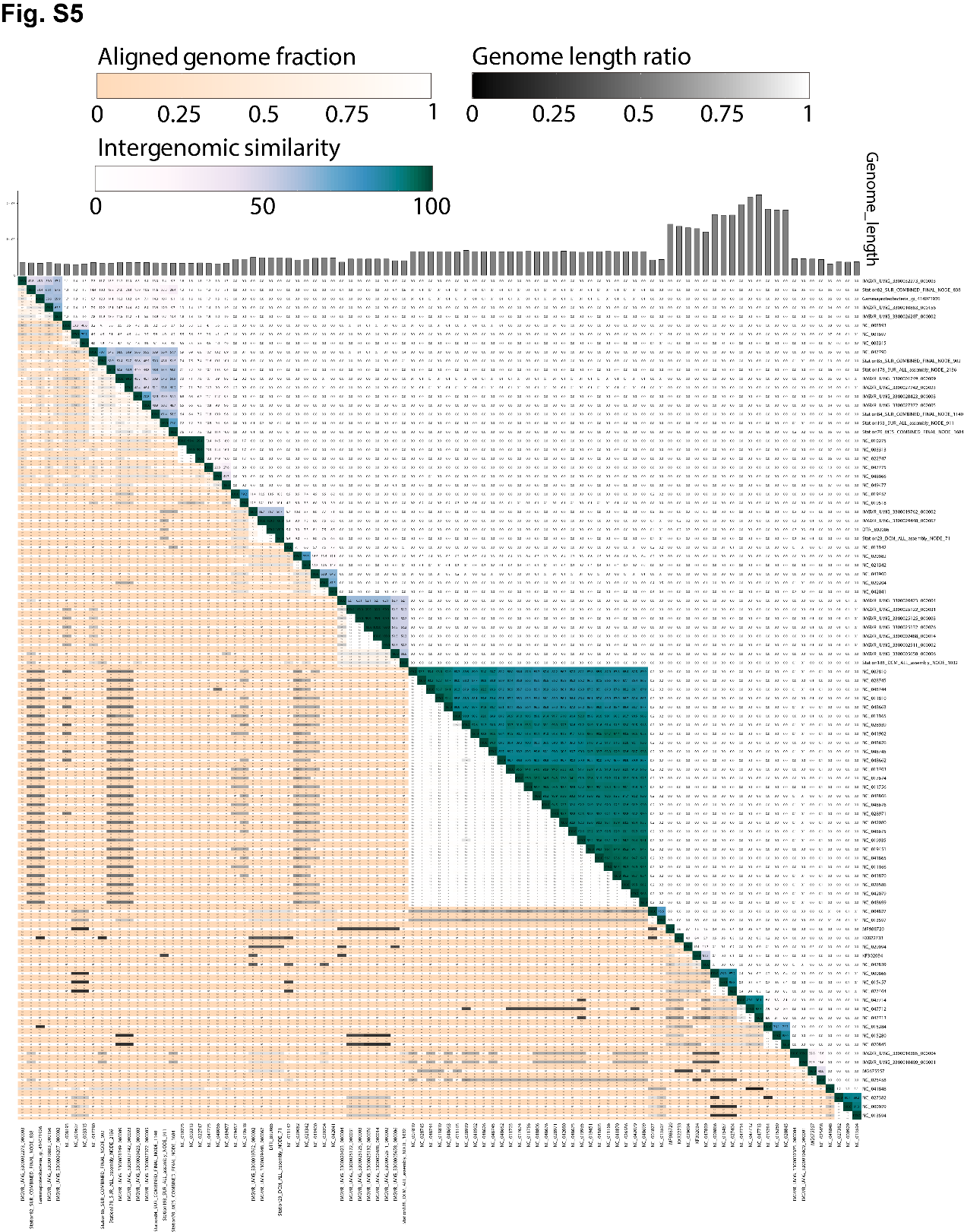


Fig. S5 The heatmaps generated by the VIRIDIC, showing the intergenomic relationships of *Siphoviridae*, *Myoviridae*, *Podoviridae/Zebellviridae/Schitoviridae*, *Autographiviridae* and *Inoviridae*, respectively. The intergenomic average nucleotides identity between transient pairs was shown in the right half of the heatmaps. The darker colors emphasize low values, indicating genome fractions are expected to decrease with increasing the distance between the phages. The boundary in the right half of the heatmaps indicates different genus-leveled VC.


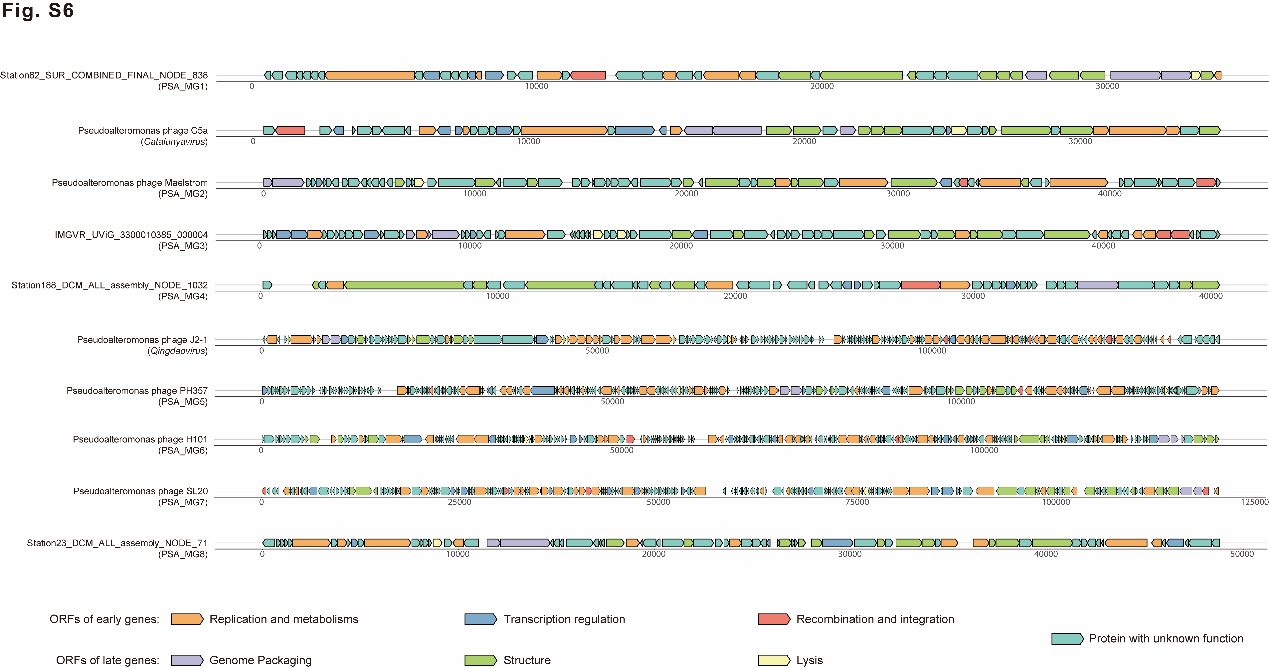


Fig. S6 The gene maps of representative Pseudoalteromonas-associated phage genomes (PSAPGs) in *Siphoviridae*, *Myoviridae*, *Podoviridae/Zebellviridae/Schitoviridae*, *Autographiviridae* and *Inoviridae*, respectively. The six functional modules were classified and indicated by different colors. The potential metabolism auxiliary genes (AMGs) were classified into the ‘Replication and metabolisms’ modules here.


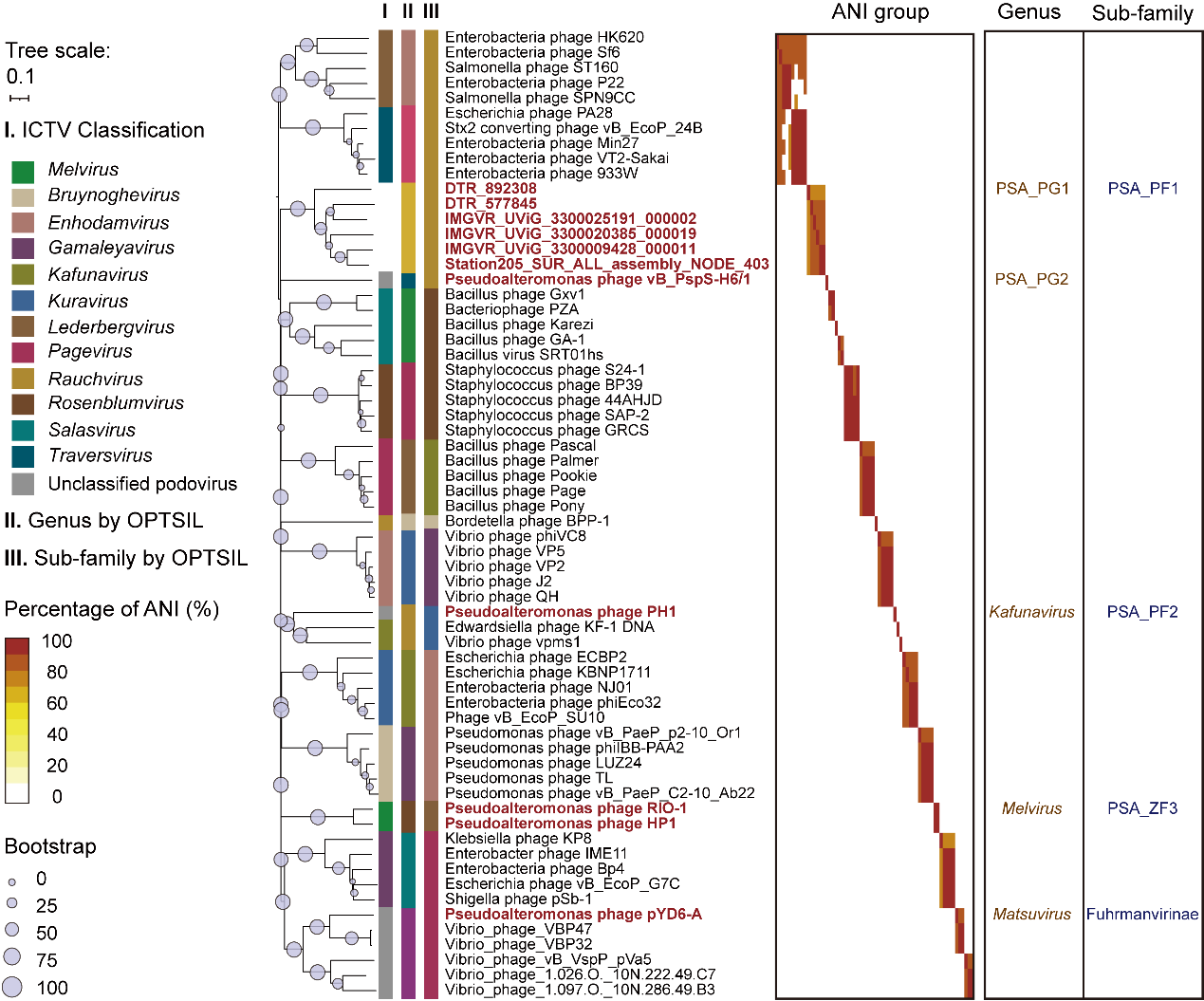


**Fig. S7.** Whole-genome based phylogenic trees and average nucleotide identity (ANI) clustering of 11 Pseudoalteromonas phages of *Podoviridae/Zebellviridae/Schitoviridae* with Their references*.* The genus-leveled viral clusters were assigned by OPTSIL, correcting and verifying by ANI clustering and VIRIDIC. Four subfamilies and proposed subfamilies were assigned, including two belonging to *Podoviridae*, one belonging to *Zobellviridae*, and one belonging to *Schitoviridae*. Five genera and proposed genera were assigned, including three belonging to *Podoviridae*, one belonging to *Zobellviridae*, and one belonging to *Schitoviridae*. The bootstrap of each branch node was indicated by circle proportional to the value. Three series of color boxes behind the tree indicate: I. Genera classified by ICTV; II. Genus-leveled VCs of all these *Pseudoalteromonas* phages classified by OPTSIL; III. Subfamily-leveled viral clusters of all these *Pseudoalteromonas* phages classified by OPTSIL. Legends of tree scale, number of bootstraps, ICTV classification and ANI are indicated on the leftward of the tree.


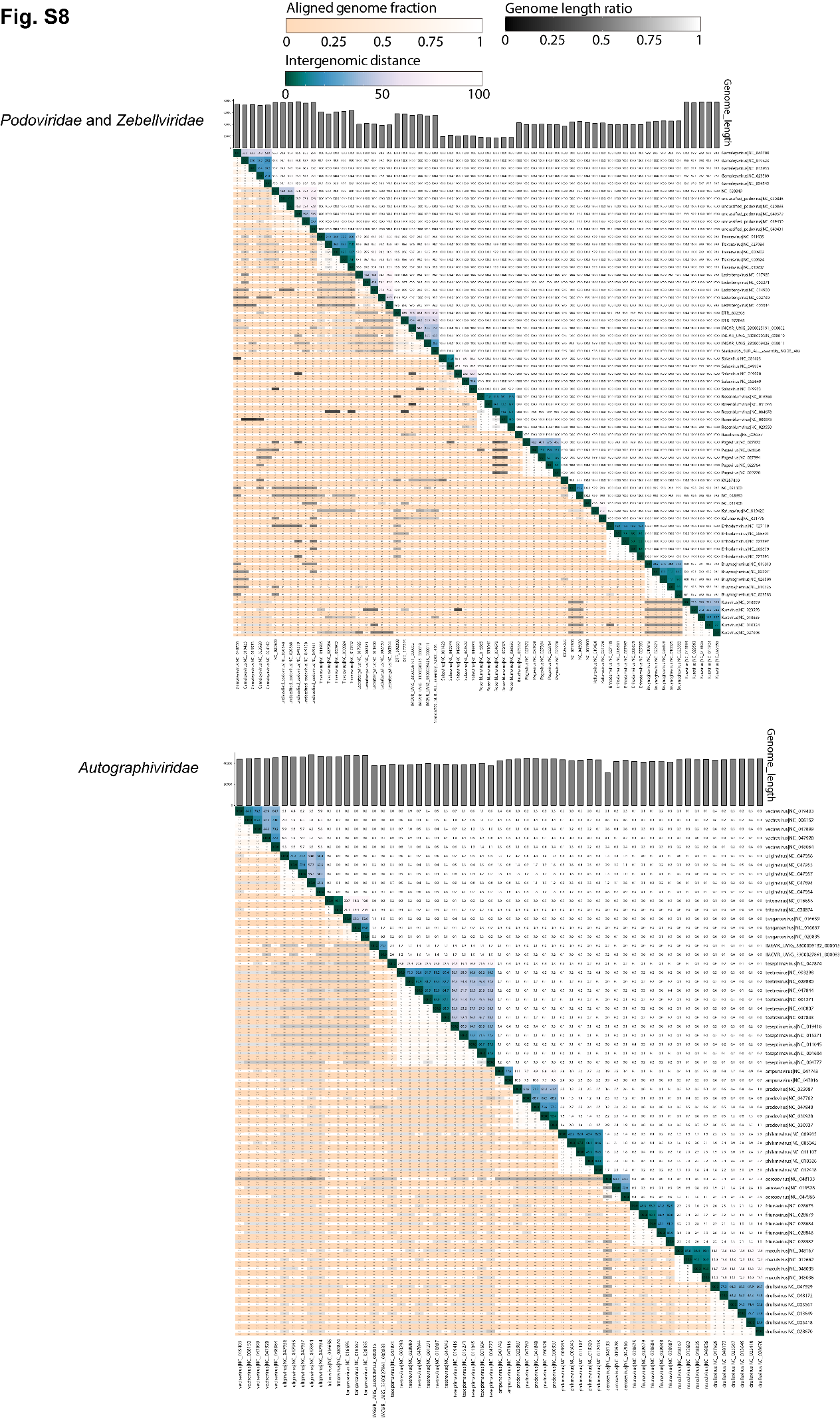


Fig. S8 The heatmaps generated by the VIRIDIC, showing the intergenomic relationships of *Siphoviridae*, *Myoviridae*, *Podoviridae/Zebellviridae/Schitoviridae*, *Autographiviridae* and *Inoviridae*, respectively. The intergenomic average nucleotides identity between transient pairs was shown in the right half of the heatmaps. The darker colors emphasize low values, indicating genome fractions are expected to decrease with increasing the distance between the phages. The boundary in the right half of the heatmaps indicates different genus-leveled VC.


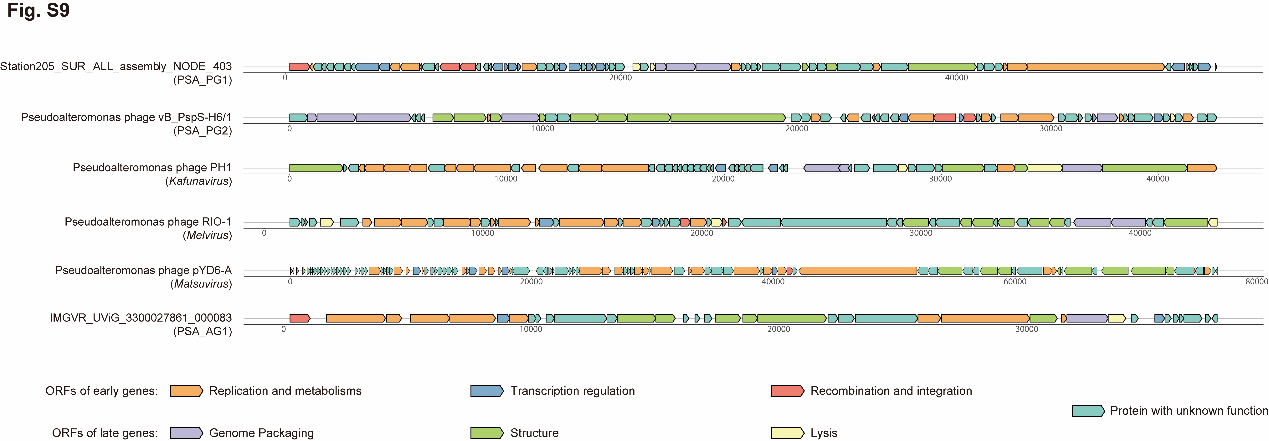


Fig. S9 The gene maps of representative Pseudoalteromonas-associated phage genomes (PSAPGs) in *Siphoviridae*, *Myoviridae*, *Podoviridae/Zebellviridae/Schitoviridae*, *Autographiviridae* and *Inoviridae*, respectively. The six functional modules were classified and indicated by different colors. The potential metabolism auxiliary genes (AMGs) were classified into the ‘Replication and metabolisms’ modules here.


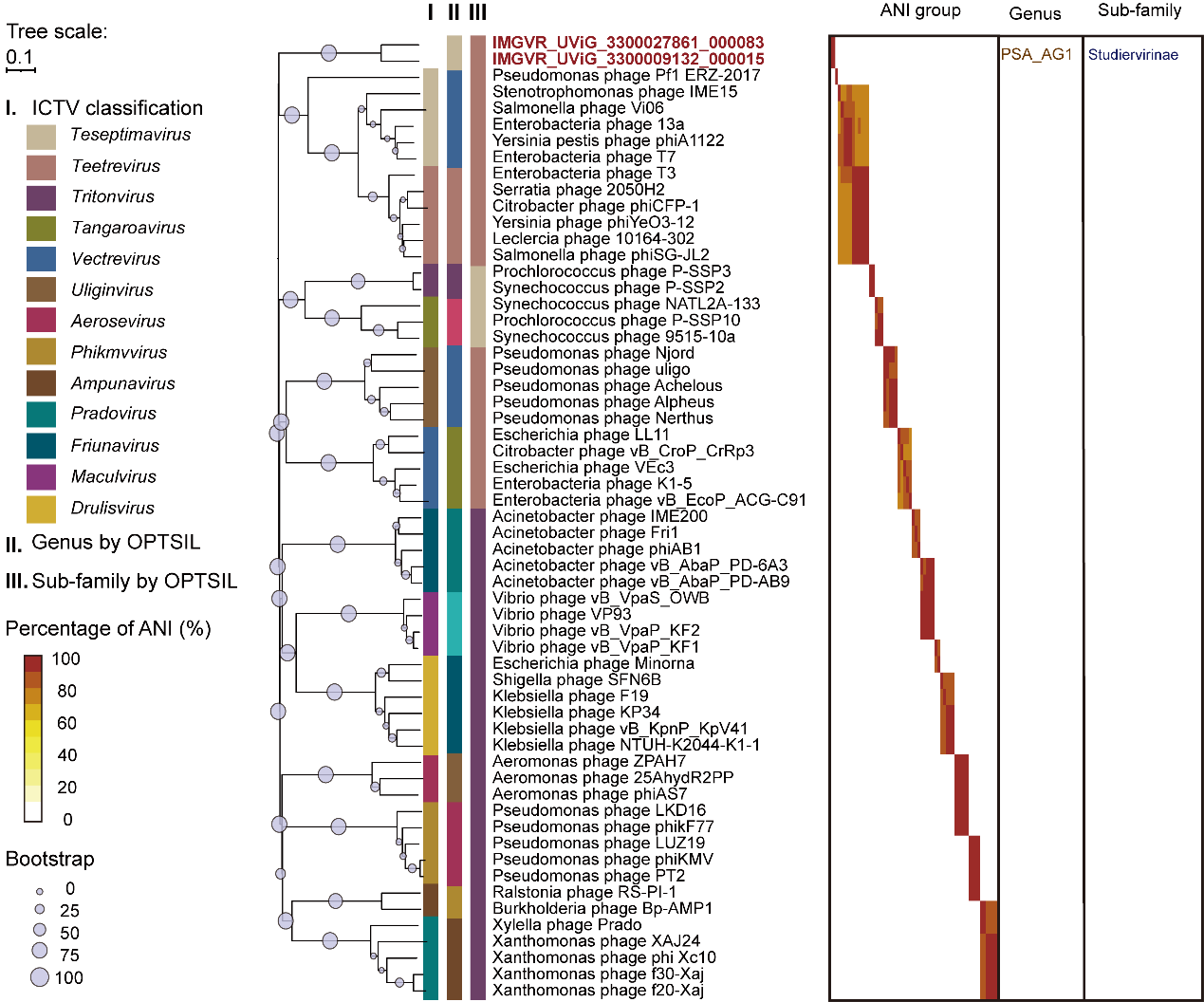


**Fig. S10.** Whole-genome based phylogenic trees and average nucleotide identity (ANI) clustering of two *Pseudoalteromonas* Autographiviral genomes with Autographiviral references*.* The one subfamily-leveled viral clusters belonging to *Autographiviridae* were assigned by OPTSIL, correcting and verifying by ANI clustering and VIRIDIC, including one genera and proposed genera. The bootstrap of each branch node was indicated by circle proportional to the value. Three series of color boxes behind the tree indicate: I. Genera classified by ICTV; II. Genus-leveled VCs of all these *Pseudoalteromonas* phages classified by OPTSIL; III. Subfamily-leveled viral clusters of all these *Pseudoalteromonas* phages classified by OPTSIL. Legends of tree scale, number of bootstraps, ICTV classification and ANI are indicated on the leftward of the tree.


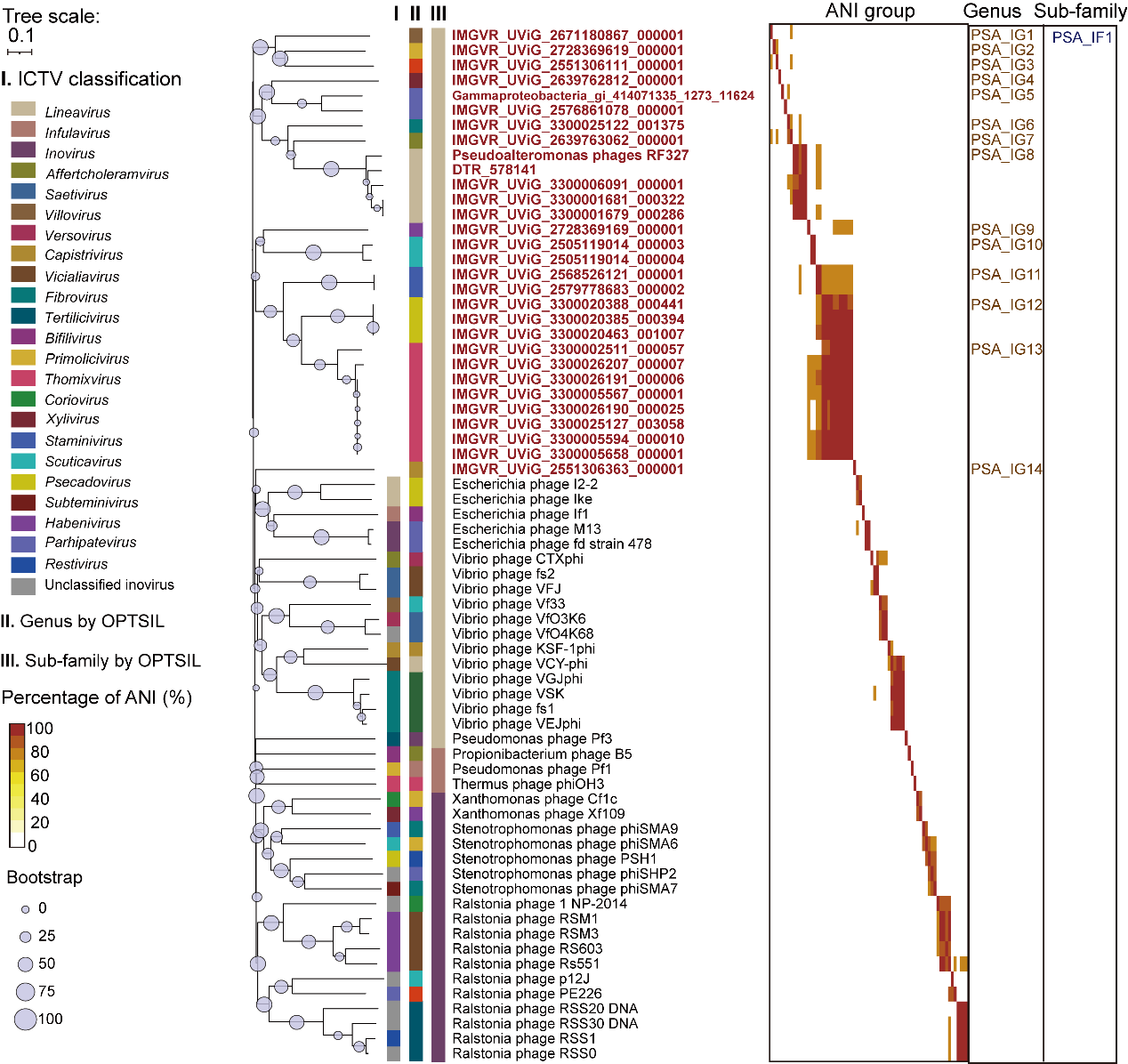


**Fig.** **S11**. Whole-genome based phylogenic trees and average nucleotide identity (ANI) grouping of 32 *Pseudoalteromonas* filamentous phages with reference sequences. The genus-leveled viral clusters were assigned by OPTSIL, correcting and verifying by ANI clustering and VIRIDIC. One proposed subfamily (*IF_1*) was assigned for these filamentous phages, and 14 proposed genera were included in *IF_1*. Nearly all of proposed genera were only consisted of uncultured filamentous phages except RF327. The bootstrap of each branch node was indicated by circle proportional to the value. Three series of color boxes behind the tree indicate: I. Genera classified by ICTV; II. Genus-leveled VCs of all these *Pseudoalteromonas* phages classified by OPTSIL; III. Subfamily-leveled viral clusters of all these *Pseudoalteromonas* phages classified by OPTSIL. Legends of tree scale, number of bootstraps, ICTV classification and ANI are indicated on the leftward of the tree.


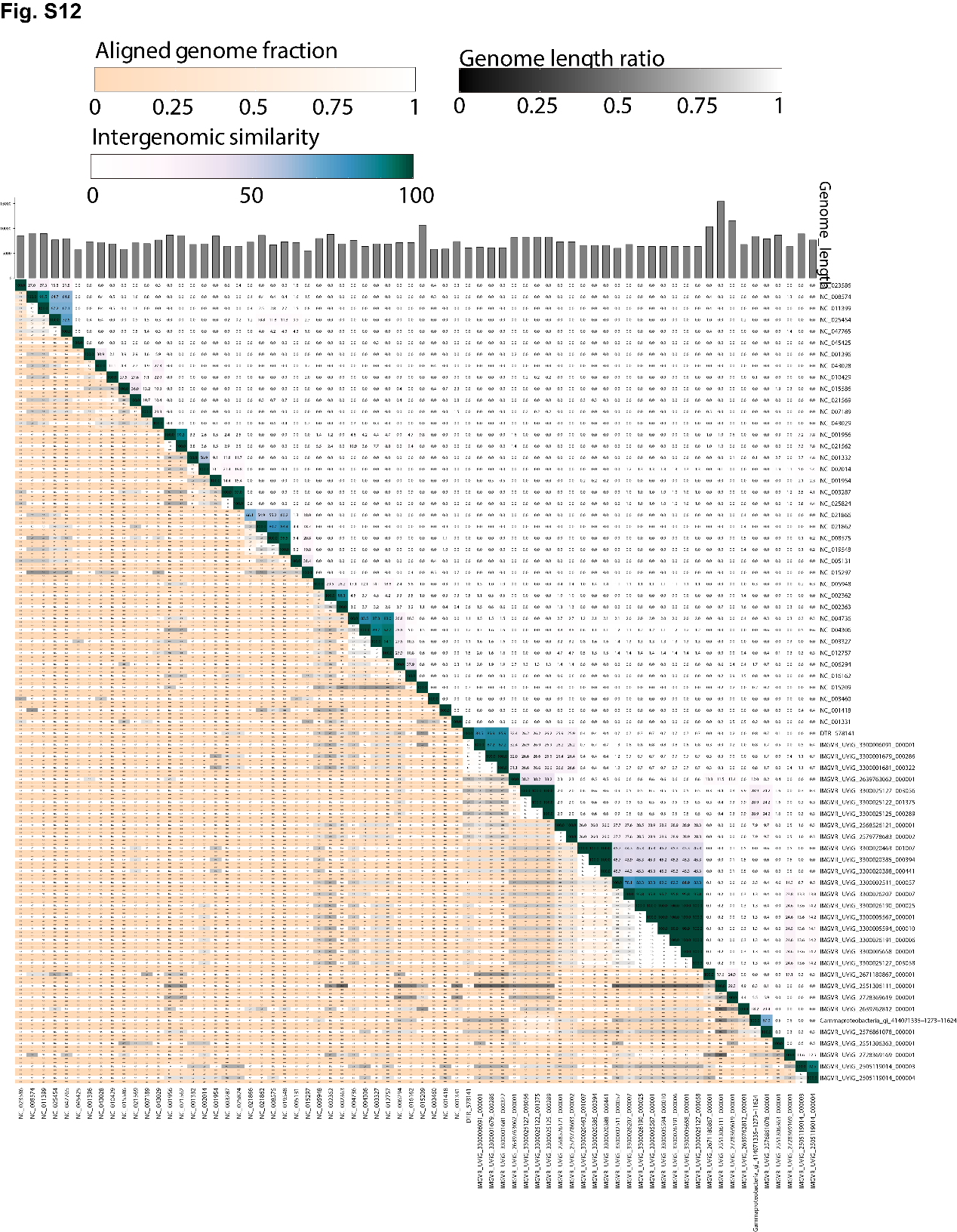


Figs. S12 The heatmaps generated by the VIRIDIC, showing the intergenomic relationships of *Siphoviridae*, *Myoviridae*, *Podoviridae/Zebellviridae/Schitoviridae*, *Autographiviridae* and *Inoviridae*, respectively. The intergenomic average nucleotides identity between transient pairs was shown in the right half of the heatmaps. The darker colors emphasize low values, indicating genome fractions are expected to decrease with increasing the distance between the phages. The boundary in the right half of the heatmaps indicates different genus-leveled VC.


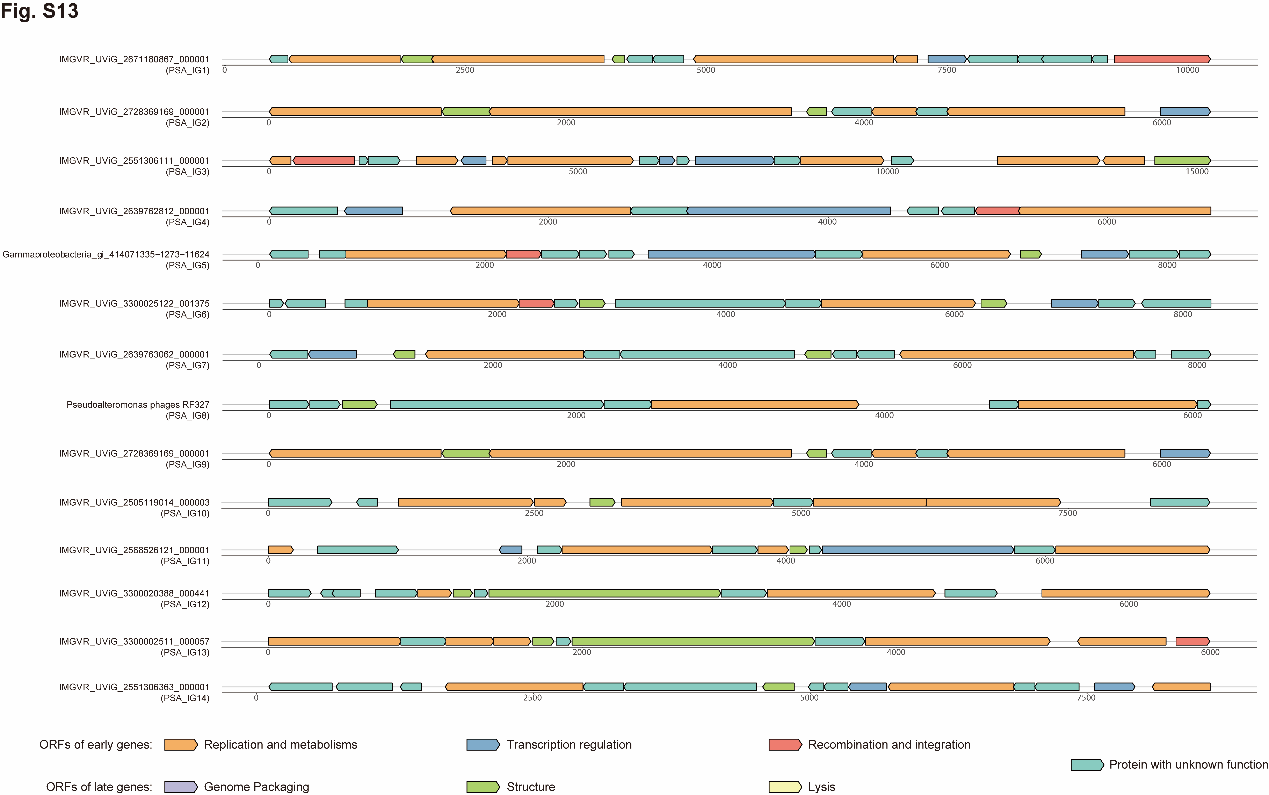


Fig. S13 The gene maps of representative Pseudoalteromonas-associated phage genomes (PSAPGs) in *Siphoviridae*, *Myoviridae*, *Podoviridae/Zebellviridae/Schitoviridae*, *Autographiviridae* and *Inoviridae*, respectively. The six functional modules were classified and indicated by different colors. The potential metabolism auxiliary genes (AMGs) were classified into the ‘Replication and metabolisms’ modules here.


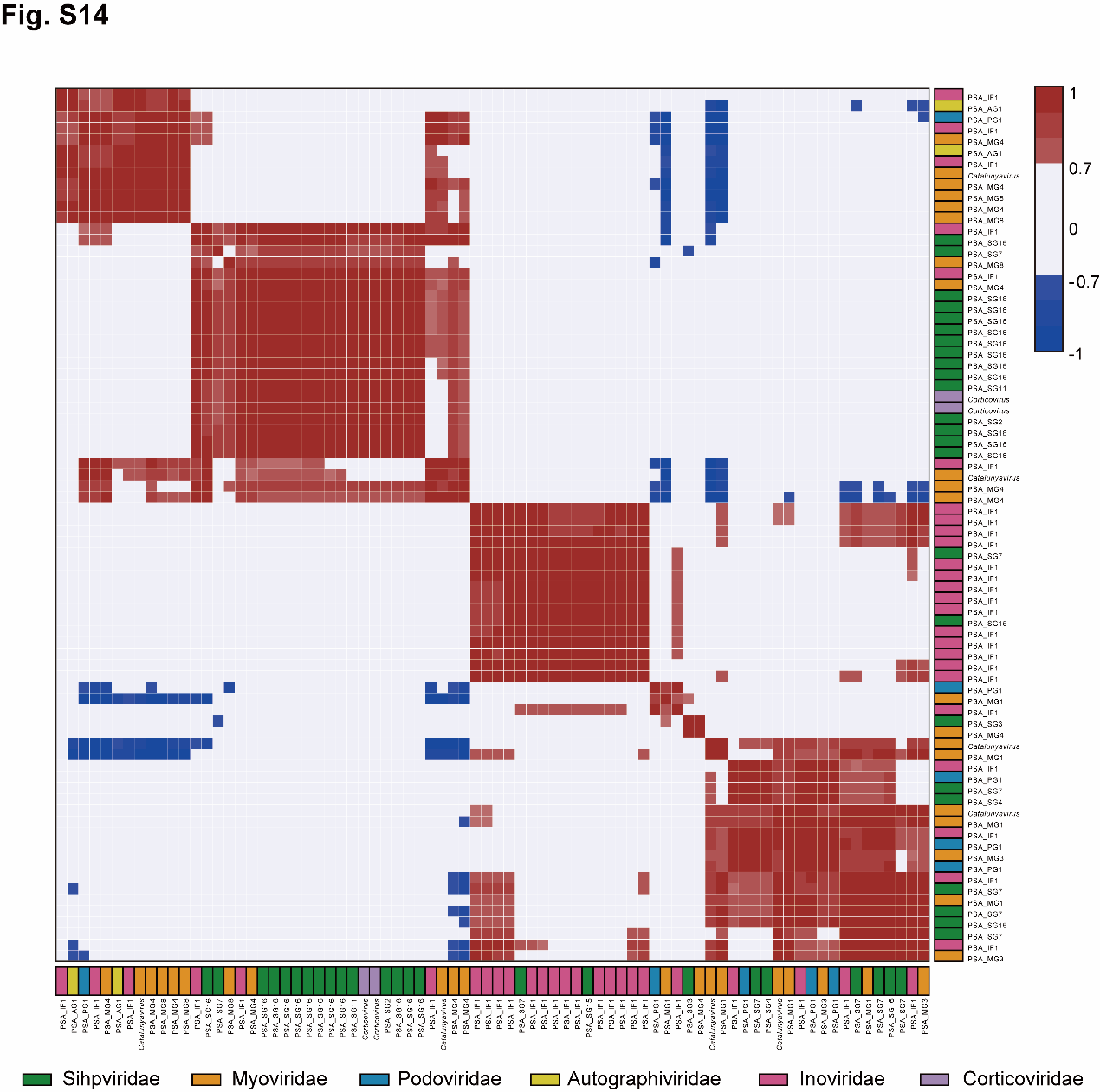


Fig. S14 The Person’s correlation coefficient heatmap of *Pseudoalteromonas* phages based on the relative abundance.
